# Longitudinal MEMRI analysis of brain phenotypes in a mouse model of Niemann-Pick Type C disease

**DOI:** 10.1016/j.neuroimage.2020.116894

**Published:** 2020-05-15

**Authors:** Harikrishna Rallapalli, Benjamin C. Darwin, Estefania Toro-Montoya, Jason P. Lerch, Daniel H. Turnbull

**Affiliations:** a Skirball Institute of Biomolecular Medicine and Department of Radiology, New York University School of Medicine, New York, NY, USA; b Biomedical Imaging & Technology Graduate Program, New York University School of Medicine, USA; c Mouse Imaging Centre, The Hospital for Sick Children, Toronto, Canada; d Department of Medical Biophysics, University of Toronto, Toronto, Canada

## Abstract

Niemann-Pick Type C (NPC) is a rare genetic disorder characterized by progressive cell death in various tissues, particularly in the cerebellar Purkinje cells, with no known cure. Mouse models for human NPC have been generated and characterized histologically, behaviorally, and using longitudinal magnetic resonance imaging (MRI). Previous imaging studies revealed significant brain volume differences between mutant and wild-type animals, but stopped short of making volumetric comparisons of the cerebellar sub-regions. In this study, we present longitudinal manganese-enhanced MRI (MEMRI) data from cohorts of wild-type, heterozygote carrier, and homozygote mutant NPC mice, as well as deformation-based morphometry (DBM) driven brain volume comparisons across genotypes, including the cerebellar cortex, white matter, and nuclei. We also present the first comparisons of MEMRI signal intensities, reflecting brain and cerebellum sub-regional Mn^2+^-uptake over time and across genotypes.

## 1. Introduction

Niemann-Pick type C (NPC) is a rare, autosomal recessive disease characterized by an inability of the body to metabolize and dispose of cholesterol and other lipids (Carstea et al., 1997). NPC is caused mostly by mutations in the *NPC1* gene (approximately 95% of cases), and the remaining cases are caused by mutations in the *NPC2* gene. Manifestations of the disease include neonatal jaundice, splenomegaly, ataxia, and progressive neurodegenerative impairment of motor and intellectual function. Most often, the onset of symptoms occurs in early childhood, leading to death within a decade. Unfortunately, diagnosis is challenging since NPC is rare and disease presentation is highly variable in terms of symptom onset and severity. There is currently no known cure for NPC, although early results employing *NPC1* gene therapy or novel lipid sequestration compounds have shown some therapeutic potential (Papandreou and Gissen, 2016). Accurate models of disease progression are required to rapidly validate putative therapies.

The BALB/cNctr-*Npc1*^*m1N*^/J mouse is the most frequently used model for severe NPC (Morris et al., 1982). Studies of these mice have shown specific, patterned degeneration of the cerebellar Purkinje cells of homozygous mutants. Early disease features include reduced body weight, hunched posture, thin fur coat, and stunted development. Homozygotes also exhibit gait ataxia, hind limb paralysis, and intention tremors at late disease stage. As in the human condition, heterozygotes do not exhibit any of these features and are phenotypically indistinguishable from wild-type (WT) littermates. In addition to histological and behavioral phenotyping of these animals, longitudinal neuroimaging studies have been performed (Totenhagen et al., 2017; Maue et al., 2012). These studies showed gross morphological reductions in the brains of mutant mice when compared to control littermates, though they stopped short of analyzing cerebellar layers and sub-regions. This was due, in part, to insufficient tissue-specific contrast afforded by non-contrast enhanced T2-weighted MRI of the mouse brain.

Manganese-enhanced MRI (MEMRI) has been shown to significantly improve contrast in the developing and adult mouse brain (Wadghiri et al., 2004; Watanabe et al., 2002). Region specific contrast enhancement is apparent in the olfactory bulb, hippocampus, and the cerebellum. Given this focal enhancement, in addition to increases in whole brain signal intensity, it is possible to specifically probe volume differences in brain sub-regions using MEMRI (Szulc et al., 2015).

With large neuroimaging datasets comes the problem of time-efficient and accurate data analysis. Expert delineation of fine brain structures is time consuming, but still remains the gold-standard of anatomical evaluation. However, the number of trained experts is small, and inter- and intra-rater variability limits the reliability of manual measurements. To mitigate these issues, semi-automated or fully-automated image registration workflows have been developed (Friedel et al., 2014). Individual volumes can be registered into a well-defined atlas space and the resultant transformations from the registration chain can be manipulated to produce deformation-based measurements of volume.

In this study, we followed cohorts of BALB/cNctr-*Npc1*^*m1N*^/J homozygous mutant mice (*Npc*^−*/*−^) and their control wildtype (WT) and heterozygous (*Npc*^+/−^) littermates using MEMRI at critical timepoints of disease progression. The imaging data were registered together using a well-established processing pipeline and the resulting transforms were used to make volumetric measurements. Since MEMRI signal intensities have been shown in some cases to reflect brain function (Lin and Koretsky, 1997; Yu et al., 2005), quantitative comparisons of brain sub-region MEMRI signal intensities were also made after correction for volume differences.

## 2. Methods

### 2.1. Animals

All mice used in this study were maintained under protocols approved by the Institutional Animal Care and Use Committee at New York University School of Medicine. Mice carrying a homozygous mutation in the *Npc1* gene were generated by breeding together BALB/cNctr-*Npc1*^*m1N*^/J heterozygous animals obtained from The Jackson Laboratory (Stock No: 003092). Progeny of this cross were genotyped at postnatal day 21, using PCR of tail DNA to identify homozygotes (*Npc*^−*/*−^), heterozygotes (*Npc*^+/−^), and wild-type (*Npc*^+*/*+^), WT) mice.

### 2.2. MEMRI

A 30 mM solution of manganese chloride (MnCl_2_) tetrahydrate (Sigma-Aldrich-221279) in isotonic saline was injected intraperitoneally (IP) 24 h before each imaging session at a dose per weight of 0.5 mmol/kg (62.5 mg MnCl_2_ per kg body weight). This MnCl_2_ dose was similar to the dose documented in our previous work using MEMRI for brain and brain tumor imaging (Suero-Abreu et al., 2014; Rallapalli et al., 2020). At this dose, no chronic adverse effects were observed as a consequence of MnCl_2_ administration in this study.

Mice were imaged on postnatal weeks (W) 3, 6, and 9, where each timepoint was the specified week ± 1 day. MRI was performed using a 7 T, 200 mm diameter horizontal bore magnet (Magnex Scientific) interfaced to a Bruker Biospec Avance II console (Bruker BioSpin MRI) with actively shielded gradients (750 mT/m; BGA9s; Bruker) and using a 25 mm quadrature Litzcage coil (Doty Scientific). Animals were anesthetized using isofluorane at 1.5 L/min compressed air flow rate (3% isofluorane for induction and 1–2% isofluorane for maintenance) delivered through a nose cone. Core body temperature (range: 34–37 °C) and respiration rate were monitored using a MRI-compatible thermocouple and respiratory pillow (SAII; SA Instruments). Custom 3D-printed plastic sleds were produced to secure mice of varying size.

T1-weighted MEMRI sessions were conducted using the following protocol: 1 min, low-resolution pilot; 21 min, 100 μm isotropic resolution, spoiled 3D gradient echo sequence (echo time/repetition time, TE/TR = 3/30 ms; flip angle, FA = 30°; field of view, FOV = 1.8 × 0.9 × 1.6 cm; Matrix size = 180 × 90 × 160 voxels).

### 2.3. Image registration

Image registration allows quantification of anatomical differences between images in a high-throughput manner. We followed the Pydpiper (Friedel et al., 2014) framework in our study. Images were linearly (6 parameter followed by a 12 parameter) and non-linearly registered together using ANTS (Avants et al., 2007) to create an average image of the entire study population. In order to preserve acquisition-specific signal intensity variation for quantitative analyses, population intensity normalization (‘inormalize’) and nonuniformity correction (‘N3’) stages were disabled. At the completion of this registration, all images had been deformed into alignment with each other in an unbiased fashion. This potentiated deformation based morphometry (DBM), or the analysis of the transformations required to register the anatomy of each individual mouse into the final consensus space. From the deformation fields, Jacobian determinants (local deformation magnitudes) were calculated for each transformation, providing statistics for cross-genotype comparison at W3, W6, and W9 (Whittaker et al., 2017). Absolute Jacobians (without removal of overall linear transformations) were used to quantify localized voxel-wise expansion and contractions in volume. Such measurements also allowed for visualization of local growth rate differences between WT and *Npc*^−*/*−^ animals.

### 2.4. Automated segmentation

We employed the MAGeT Brain (Chakravarty et al., 2013) procedure for automated tissue segmentation using the Dorr-Steadman-Ulman-Richards-Qiu-Egan (DSURQE) atlas (182 structures, freely available at: https://wiki.mouseimaging.ca/display/MICePub/Mouse+Brain+Atlases) (Dorr et al., 2008; Steadman et al., 2014; Ullmann et al., 2013; Richards et al., 2011). In brief, our application of MAGeT generated a set of template atlases by non-linearly aligning the DSURQE atlas to a subset of the input images. Then, the input images were aligned to each of these templates. Finally, a voxel-voting procedure selected the most common label for each voxel among the set of segmentations.

### 2.5. Statistical analyses

Statistical analyses were performed in R (R, v3.5.0) with image analysis-specific tools exposed through RMINC (Lerch, 2006). Imaging voxel-wise linear mixed-effects models were used to assess the effects of genotype (WT, *Npc*^+/−^, or *Npc*^−*/*−^) and time (W3, W6, or W9) on Jacobians (Bates et al., 2015). These models also allowed for small variation between subjects, as some animals were imaged longitudinally (i.e. across multiple timepoints) and others cross-sectionally (i.e. one timepoint only). The form of the linear mixed effects-model – applied using the ‘mincLmer’ function - used in our analysis can be written as follows:

$$y_{i}=\beta_{0}+\beta_{1}x_{1i}+S_{i}+\varepsilon$$

Individual voxels were denoted by (i); the interaction between genotype and time (*x*_1*i*_) was a fixed-effects regressor with corresponding fixed-effects coefficient (*β*_1_); we allowed for a fixed-effect offset (*β*_0_); accounted for subtle, subject-specific variation as a random-effect (*S*_*i*_); and assumed normally distributed error (*ε*). The log Jacobian determinant for each voxel (*y*_*i*_) was our response variable. Denominator degrees of freedom were estimated using Sattherwaite’s method through ‘min-cLmerEstimateDF’. Then the ‘mincFDR’ function was used to compute the False Discovery Rate (Benjamini and Hochberg, 1995; Genovese et al., 2002) (FDR) and the ‘thresholds’ function computed statistically significant effect size thresholds after FDR correction at multiple confidence intervals. The voxel-wise *Npc*^−*/*−^ subset of *β*_1_ parameters were mapped for visualization of growth rate deviations from WT.

The DSURQE atlas shares hierarchical definitions with the Allen Brain Institute’s mouse brain atlas (Lein et al., 2007), which made it possible to pull labels for the cortex, hindbrain, hippocampus, hypothalamus, midbrain, olfactory bulb, thalamus, cerebellum, and cerebellar sub-regions programmatically. Region-specific volume and signal cross-genotype comparisons were made using Tukey’s method (Tukey, 1949). The relative signal intensity in each brain region and sub-region were normalized to the whole brain signal:

$$\text{Relative signal intensity}=\frac{{\text{Mean signal per voxel}}_{\text{Region}}}{{\text{Mean signal per voxel}}_{\text{Whole Brain}}}$$

Plots of quantitative volume and signal data include 95% confidence intervals as error bars about the mean of the measurement. Non-overlapping 95% confidence intervals indicate significant differences.

Raw data, R-code, and supplemental analyses are included in Supplementary Data 1.

## 3. Results

### 3.1. MEMRI reveals progressive degeneration and signal intensity differences

**Fig. 1.**
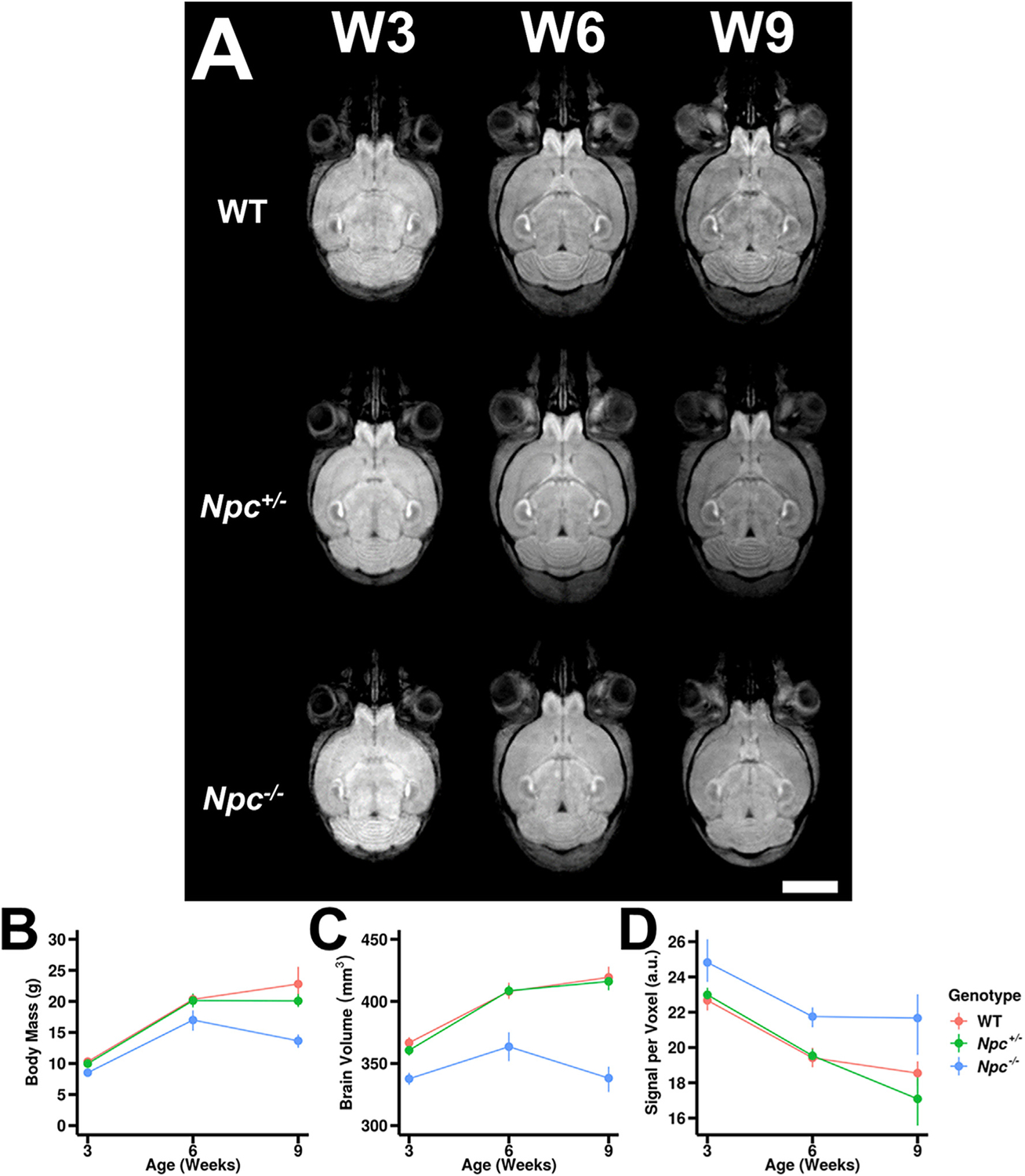
MEMRI of *Npc* wild type, heterozygote and homozygote mouse brains reveal volumetric and signal differences across genotypes. (A) Compared to wild-type (WT) brains, heterozygotes (*Npc*^+/−^) were similar in signal intensity from W3 to W9 and experienced similar growth trends over this period. In contrast, homozygote (*Npc*^−*/*−^) brains were relatively hyperintense across all timepoints compared to controls and experienced noticeable volume reduction from W6 to W9. Scale bar – 3 mm (B) Animal mass trends over the study period show WT (red) and *Npc*^+/−^ (green) animals did not differ significantly in their growth (p = 0.2658), whereas *Npc*^−*/*−^ (blue) mice were significantly underweight (p <0.0001). (C) Brain volume quantitative trends were similar to those of animal mass trends. *Npc*^−*/*−^ animals had significantly smaller whole brain volume than WT by W9 (p <0.0001). (D) Quantitative signal trends revealed that *Npc*^−*/*−^ mouse brains were significantly hyperintense across all timepoints compared to WT and *Npc*^+/−^ (p = 0.0173). Error bars – 95% confidence intervals. N values are shown in Table 1. For complete contrast tables, please see Supplementary Data 1.

Mice were imaged both longitudinally and cross-sectionally between postnatal weeks W3 and W9 (Table 1, Fig. 1A). At W3, MEMRI from representative WT and *Npc* ^+/−^ control mice showed appreciable signal enhancement – particularly apparent in the olfactory bulb, hippocampus, and the cerebellum – consistent with previous MEMRI studies of the developing and adult mouse brain (Szulc et al., 2015; Watanabe et al., 2013). Whole brain signal reduction between W3 and W6 was apparent in *Npc*^+/−^ mice, similar to WT and *Npc* ^+/−^ mice. However, visually apparent reductions in cerebellar contrast were noticeable by W6 and progressively worsened by W9.

Across genotypes, all animals significantly gained weight from W3 to W6. Both WT and *Npc* ^+/−^ control animals also gained weight from W6 to W9, but this growth was not significant. *Npc*^−*/*−^ animals significantly lost weight from W6 to W9, as expected for end-stage progressive NPC. These animals were significantly underweight compared to control littermates across all time points (Fig. 1B).

The visual trends in brain volume were corroborated by quantitative comparisons over time (Fig. 1C). All mice experienced significant brain growth from W3 to W6, although *Npc*^−*/*−^ mice had significantly smaller brain volume than control littermates at both time points. In control animals, there was no statistically significant increase in brain volume from W6 to W9, while *Npc*^−*/*−^ mice experienced significant reductions in brain volume from W6 to W9.

Interestingly, *Npc*^−*/*−^ mouse brains were significantly hyperintense across all time points compared to control littermates (Fig. 1D). For each genotype, mouse brains followed the same trend: statistically significant reduction in signal per voxel from W3 to W6, then relative isointensity from W6 to W9.

### 3.2. Deformation-based morphometry revealed sub-region specific volume and growth rate retardation in Npc^−/−^ mice

**Fig. 2.**
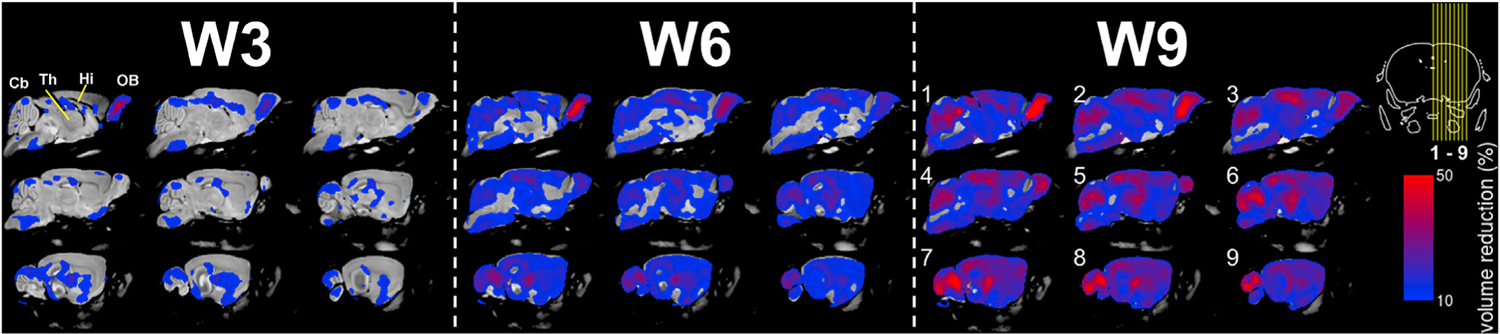
Deformation-based morphometry (DBM) reveals statistically significant voxel-wise contractions in *Npc*^−*/*−^ mouse brains. Particularly large contractions were apparent in the olfactory bulb (OB) as early as W3. Similarly large magnitude contractions were apparent in the cerebellum (Cb), hippocampus (Hi), and thalamus (Th) by W9. Insert: yellow lines indicate which sagittal orientation images and maps were rendered.

Voxel-wise DBM analyses were performed on the data from all of the mice imaged from W3 to W9 (Table 1). These analyses showed significantly smaller volumes in *Npc*^−*/*−^ mouse brains as early as W3 (Fig. 2). Of note, the olfactory bulb was significantly stunted at this early timepoint. Smaller magnitude volume reductions were apparent in several regions, including the pons, hippocampus, thalamus, and hypothalamus. Parts of the anterior cerebellum were also significantly smaller in volume, particularly in the cerebellar hemispheres.

At W6, more widespread volume reductions were apparent in the *Npc*^−*/*−^ mice. The significant effects persisted in the olfactory bulb, hippocampus, thalamus, and hypothalamus. At W6, the posterior cerebellum was also smaller in volume, in addition to the anterior cerebellum, corroborating previous reports that the Purkinje cells degenerate in an anterior-posterior/time-dependent manner (Ko et al., 2005).

At W9, there were few unaffected sub-regions in the *Npc*^−*/*−^ mouse brain. Significant volume reductions were apparent in the cortex, midbrain, and hindbrain in addition to the structures that were significantly affected at W6.

The volumetric data derived from DBM analyses were also used to estimate the growth rates in each genotype, showing that the *Npc*^−/−^ brain was significantly retarded in growth across most sub-regions compared to WT brains (Supplementary Fig. 1).

### 3.3. Fully-automated segmentation enabled quantitative brain region-wise analyses

**Fig. 3.**
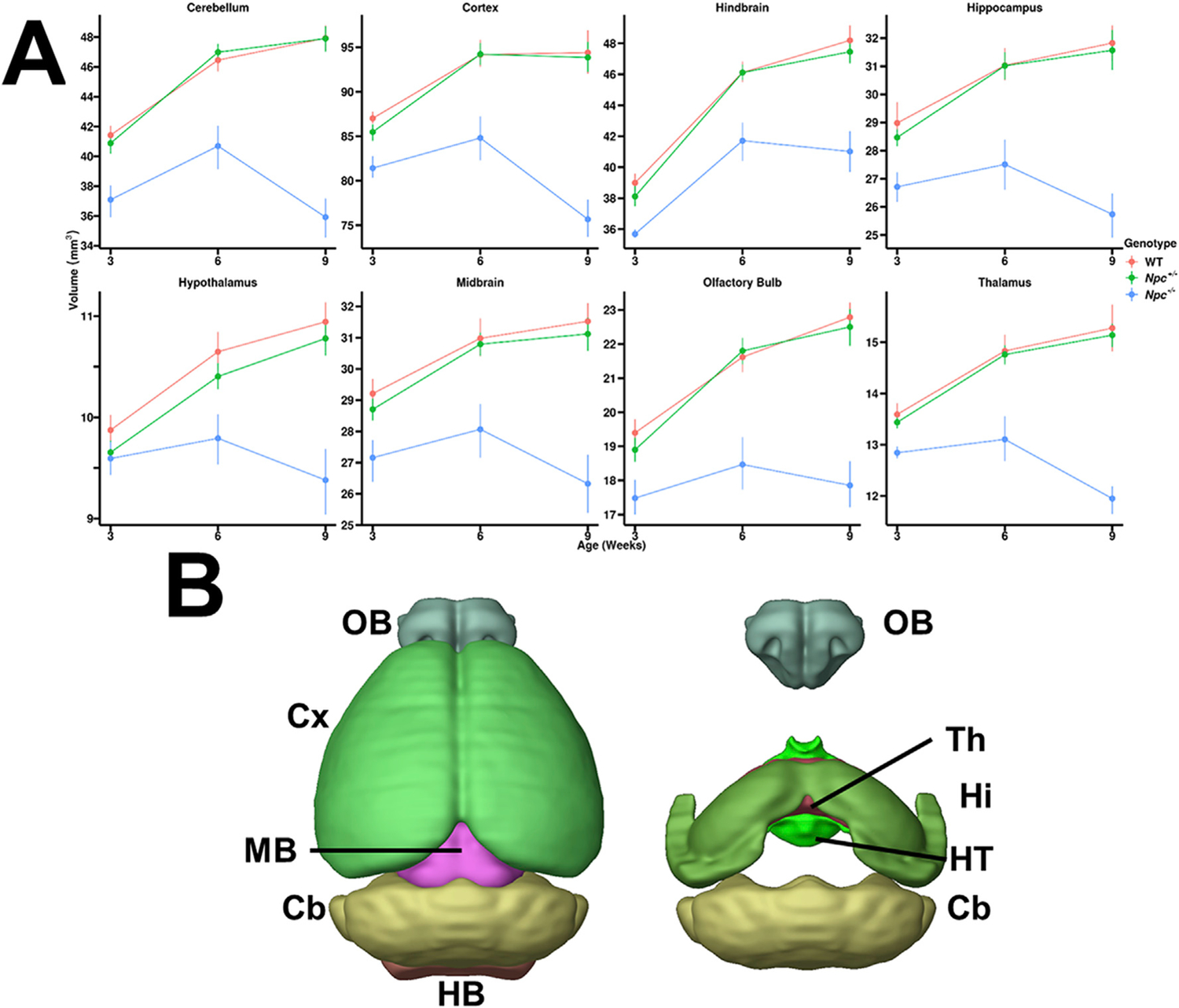
Brain sub-region quantitative volume analyses. (A) Statistically significant volume reductions were observed by W6, with marked degeneration apparent between W6 and W9 across all brain regions in *Npc*^−*/*−^ mice (blue) compared to WT (red) and *Npc*^+/−^ mice (green). Error bars – 95% confidence intervals. (B) Select regions were analyzed, shown in 3D (p-values for WT – *Npc*^−*/*−^ contrasts at W9, N values are given in Table 1): Olfactory Bulb (OB, p <0.0001), Cortex (Cx, p <0.0001), Midbrain (MB, p <0.0001), Cerebellum (Cb, p <0.0001), Hindbrain (HB, p <0.0001), Hypothalamus (HT, p <0.0001), Hippocampus (Hi, p <0.0001), and Thalamus (Th, p <0.0001).

Consistent with the DBM results, quantitative, region-specific analyses after automated segmentation showed statistically significant reductions in the *Npc*^−*/*−^ mice compared to control WT and *Npc*^+/−^ mice (Fig. 3). The trends observed for whole brain volume changes in the *Npc*^−*/*−^ brains (Fig. 1C) were also seen in the brain sub-regions, with variable magnitude and statistical significance in the differences (Fig. 3).

The WT and *Npc*^+/−^ cerebella were similar in volume across all time points. These animals experienced statistically significant growth in the cerebellum from W3 to W6, but no significant growth from W6 to W9. In contrast, *Npc*^−*/*−^ cerebella were significantly reduced in volume across all time points compared to controls. These animals experienced statistically significant growth in the cerebellum from W3 to W6, though the cerebella significantly regressed from W6 to W9. A summary of the quantitative volumetric trends and similar trends in other brain sub-regions is given in Supplementary Table 1.

### 3.4. MEMRI signal comparisons showed brain sub-region specific trends over time

**Fig. 4.**
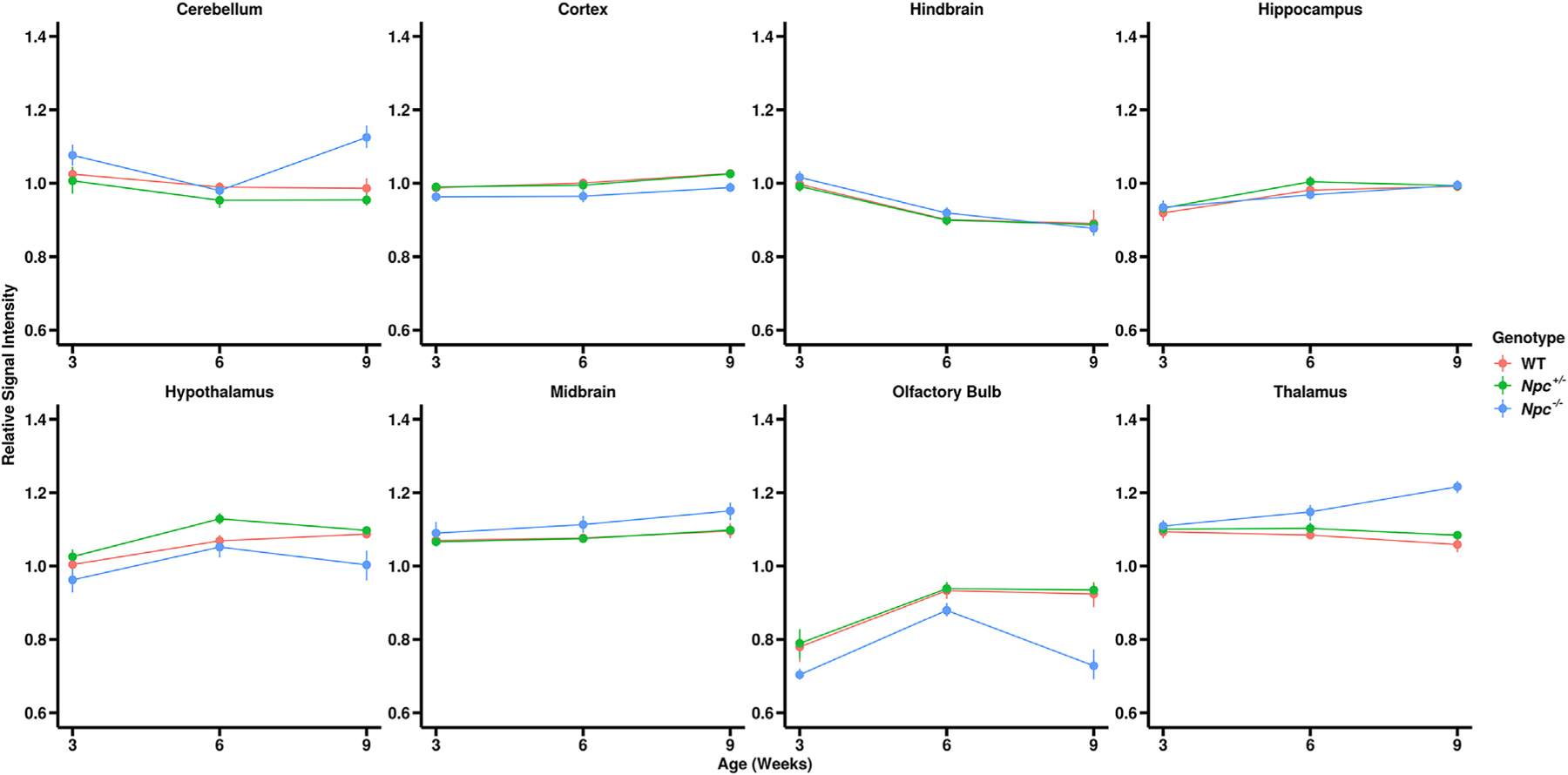
Brain sub-region quantitative relative signal analyses. Relative to whole brain signal intensity, *Npc*^−*/*−^ brain sub-regions (blue) were: relatively hyperintense in the cerebellum (p <0.0001), midbrain (p = 0.0099), and thalamus (p <0.0001); relatively hypointense in the cortex (p = 0.0268), hypothalamus (p = 0.0049), and the olfactory bulb (p <0.0001); and relatively isointense in the hindbrain (p = 0.9955) and hippocampus (p = 1.0000) compared to WT (red) and *Npc* ^+/−^ mice (green). Error bars – 95% confidence intervals. (p-values for WT – *Npc*^−*/*−^ contrasts at W9, N values are given in Table 1).

Relative signal intensity measurements from brain sub-regions were expressed as a percentage of the whole brain signal in each animal (Fig. 4). The WT and *Npc* ^+/−^ cerebella showed no significant differences in relative signal across all time-points. These control animals remained relatively isointense from W3 to W9. Interestingly, *Npc*^+/−^ cerebella showed similar relative signal intensity to WT at W3 and W6, but were significantly hyperintense at W9. A summary of the quantitative signal trends and similar trends in other brain sub-regions is given in Supplementary Table 2.

### 3.5. MEMRI enables longitudinal study of Npc^−/−^ cerebellar sub-regions

**Fig. 5.**
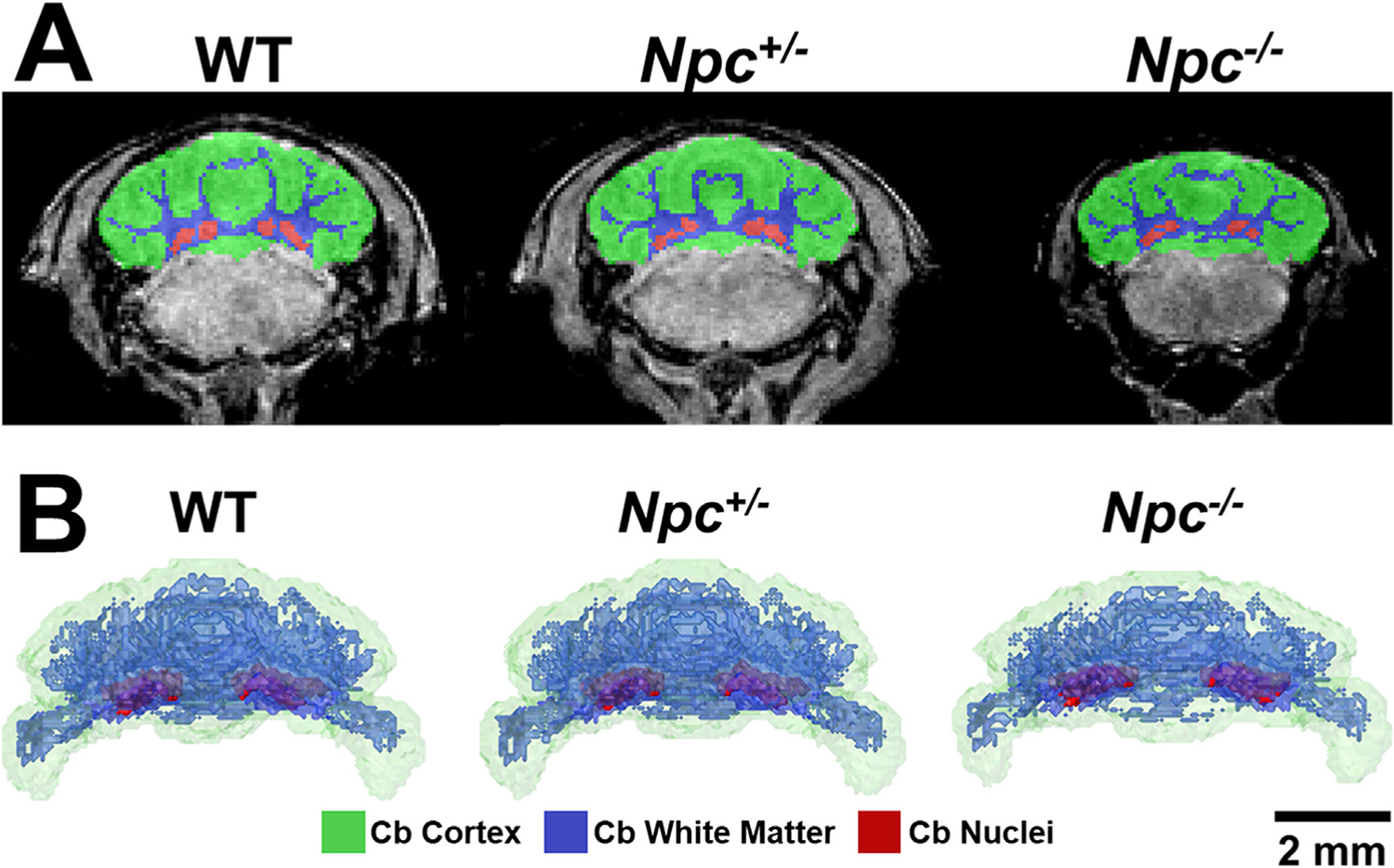
Visualization of cerebellar sub-regions. Segmentation of WT, *Npc*^+/−^, and *Npc*^−*/*−^ cerebella revealed sub-region-specific degeneracy in *Npc*^−*/*−^ brains. (A) At W9, WT and *Npc*^+/−^ cerebellar cortex (green), white matter (blue), and nuclei (red) segmentations appeared similar whereas those of *Npc*^−*/*−^ cerebella were reduced in volume. (B) These relationships were also apparent in 3D volume rendered segmentations. Over time, WT and *Npc*^+/−^ rendered segmentations showed no obvious differences in sub-region appearance. In contrast, the *Npc*^−*/*−^ cerebellar cortex (green), white matter (blue), and nuclei (red) were all reduced in volume compared to control animals and over time.

**Fig. 6.**
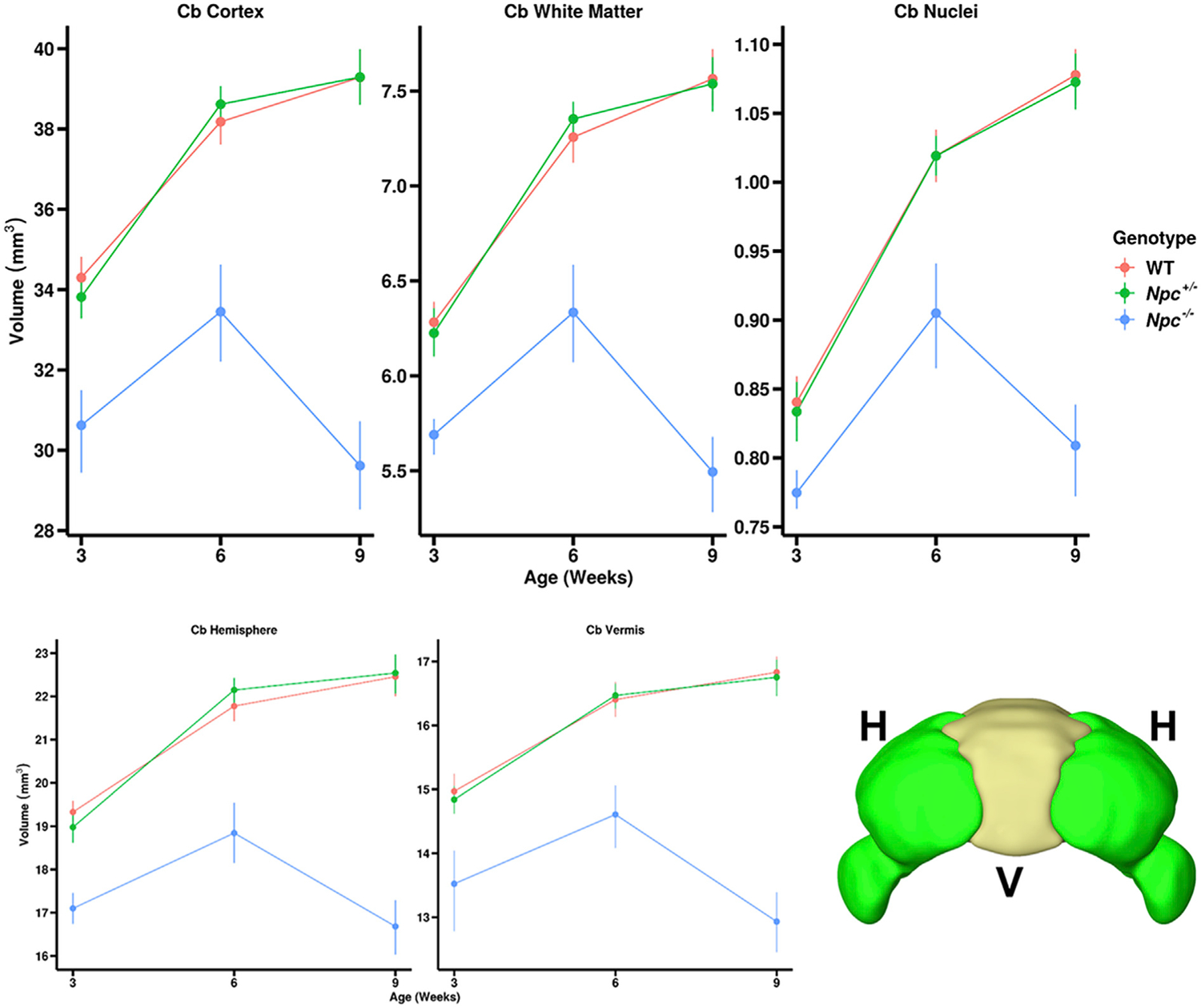
Quantification of cerebellar sub-region volumes over time corroborate visually apparent trends. Across all sub-regions and timepoints, there were no significant differences in volume between WT (red) and *Npc*^+/−^ (green) cerebella, whereas the *Npc*^−*/*−^ (blue) cerebellar cortex (p = 0.0002, <0.0001, <0.0001), white matter (p = 0.0078, <0.0001, <0.0001), and nuclei (p = 0.1283, <0.0001, <0.0001) were smaller in volume than WT over all timepoints. These trends were not different between the cerebellar hemispheres and vermis, suggesting that the *Npc*^−*/*−^ cerebellum degenerates uniformly. Inset shows the delineation between the (central) vermis and the (lateral) hemispheres. Error bars – 95% confidence intervals. (p-values for W3, W6, and W9 WT – *Npc*^−*/*−^ contrasts, N values are given in Table 1).

Since Purkinje cell degeneration is a known phenotype of *Npc*^−/−^ mutant mice, we performed detailed analyses of the cerebellar sub-regions. Automated segmentation of the MEMRI images enabled visualization (Fig. 5) and quantitative analysis (Fig. 6) of the cerebellar cortex (which includes the cell bodies and dendrites of the Purkinje cells), white matter (which includes the Purkinje cell axons) and cerebellar nuclei (CN, the target cells of the Purkinje cell axons) of WT, *Npc*^+/−^, and *Npc*^−/−^ mice. These results showed that the cerebellar sub-regions followed similar trends to the whole cerebella of each genotype. Specifically, we observed significant volume increase of the WT and *Npc*^+/−^ sub-regions between W3 and W6 and no significant growth between W6 and W9, while *Npc*^−*/*−^ sub-regions grew from W3 to W6 but then regressed between W6 and W9. In addition, the *Npc*^−*/*−^ sub-regions were all significantly reduced in volume across all time points compared to the control WT and *Npc*^+/−^ sub-regions. Similarly, comparisons of the cerebellar (central) vermis and (lateral) hemispheres showed significant reductions in volume compared to WT mice across all time points, with no obvious difference between these two sub-regions. A summary of these quantitative volumetric trends is given in Supplementary Table 3.

### 3.6. Cerebellar signal comparisons

**Fig. 7.**
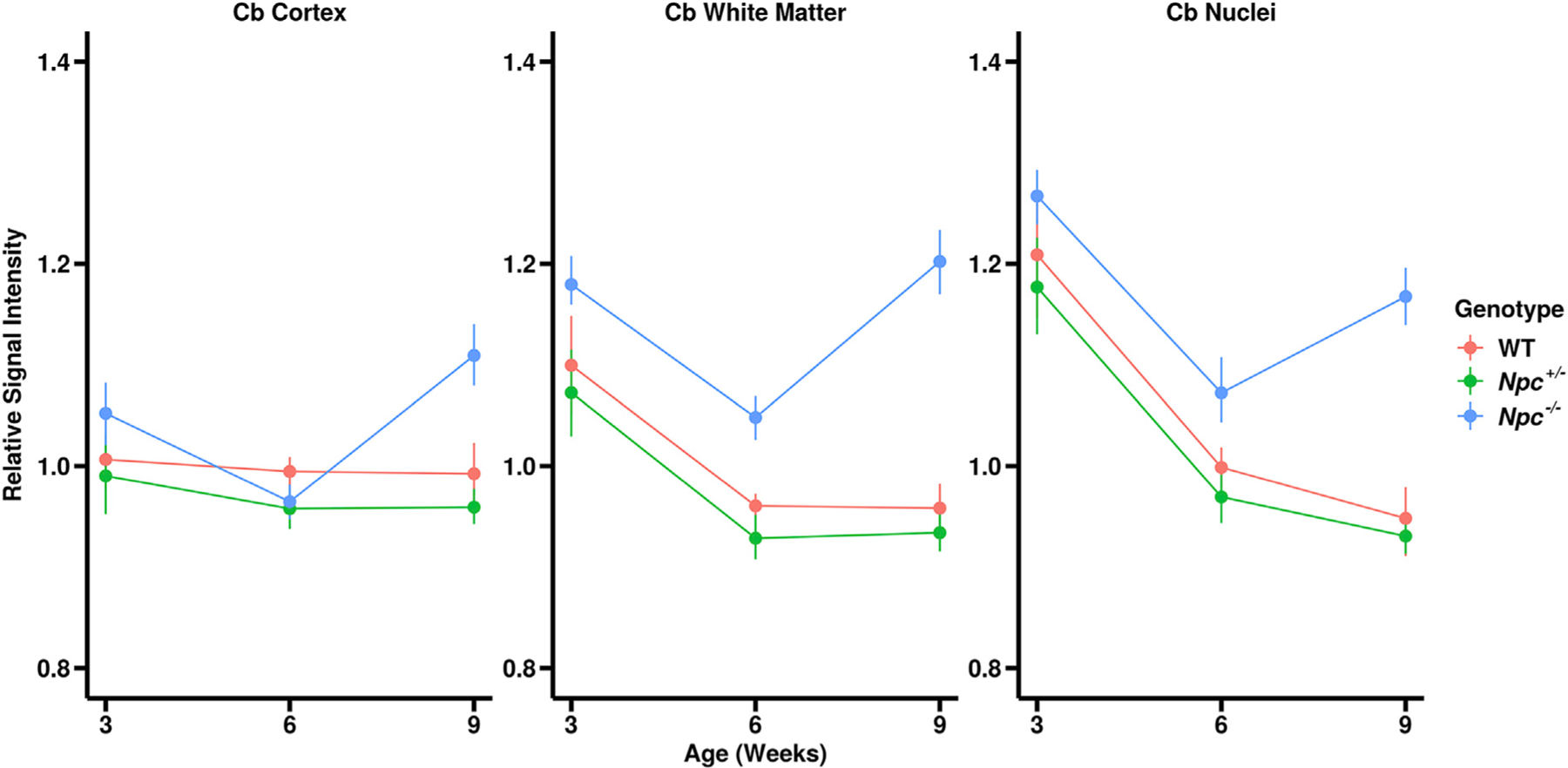
Quantitative signal trends in cerebellar sub-regions over time. As expected, there were no significant differences in relative signal between WT (red) and *Npc*^+/−^ (green) cerebellar sub-regions at any timepoint. Interestingly, the *Npc*^−*/*−^ (blue) cerebellar cortex was hyperintense at W3 and W9 and isointense at W6 compared to WT (p = 0.7557, 0.8197, 0.0002), while the cerebellar white matter (p = 0.1683, 0.0040, <0.0001) and nuclei (p = 0.7431, 0.0951, <0.0001) were hyperintense across all timepoints. Error bars – 95% confidence intervals. (p-values for W3, W6, and W9 WT – *Npc*^−*/*−^ contrasts, N values are given in Table 1).

Comparisons of cerebellar sub-regional relative signals were made over time across genotypes (Fig. 7). At W3, relative signal in the *Npc*^−/−^ cerebellar cortex, white matter and CN were increased – significantly so in the white matter and CN – compared to WT and *Npc*^+/−^ control mice. From W3 to W6, there was reduction in signal across all sub-regions and genotypes. *Npc*^−/−^ mice experienced significant reductions in signal across all three regions, whereas WT and *Npc*^+/−^ mice exhibited significant reductions only in the white matter and CN. At W6, the *Npc*^−/−^ cerebellar cortex had a similar relative intensity compared to the control mice whereas the white matter and CN were hyperintense at W6. From W6 to W9, the relative signal from all three cerebellar sub-regions remained roughly isointense in the control animals. Interestingly, relative signal from all sub-regions significantly increased from W6 to W9 in the *Npc*^−*/*−^ mice. A summary of these quantitative volumetric trends is given in Supplementary Table 4.

## 4. Discussion

In this study, we presented a novel characterization of the BALB/cNctr-*Npc1*^*m1N*^/J mouse model of NPC using MEMRI. We followed the progression of disease longitudinally and cross-sectionally in cohorts of genotype-validated animals at critical time points; performed deformation-based morphometry (voxel-wise) to visualize both subtle and severe degeneracy in the *Npc*^−*/*−^ brain; and performed brain region-wise statistical analyses of volume and signal.

We chose to use MEMRI because of our experience with this approach for studying normal cerebellar development and other mouse models of cerebellar disease. Contrast enhancement provided by MEMRI enables fine definition of brain sub-regions, potentiating focal study of the cerebellar layers known to be affected in NPC. It is also interesting that MEMRI enabled *in vivo* analyses of the cerebellar nuclei, which have recently been reported to be critical for control of cell number and sub-regional volumes during normal cerebellum development (Willett et al., 2019). In addition to the fine-level anatomical detail provided by MEMRI in the current study, previous diffusion and tract-density weighted imaging methods have revealed differences in fractional anisotropy, tract dispersion, and apparent diffusion coefficient in the white matter of children (Trouard et al., 2005) and mice (Totenhagen et al., 2012) carrying mutations in *NPC1*. Further study of diffusion in combination with the cerebellar sub-region specific contrast afforded by MEMRI may be useful in future studies of NPC.

Automated image registration, as used in this study using the well-established Pydpiper framework, enabled rapid, objective quantification of a relatively large 3D imaging dataset. The transformations were pooled and processed to produce deformation maps of *Npc*^−*/*−^ brains compared to control littermates. Use of a well-defined atlas as a target for the registration permitted automated segmentation of the individual images. These steps represent an improvement on the semi-automated approaches utilized by a previous imaging study of this mouse model, and also enabled novel visualization of NPC disease progression (Totenhagen et al., 2017). Compared to this previous study, the estimated brain volumes of the *Npc*^−*/*−^ mice and their control littermates are smaller in our study. Possible reasons for these differences could include smaller body and brain size due to larger litters and/or different diets in our colony, and differences in brain volume estimation due to a different whole brain atlas definition. Nevertheless, the relative magnitudes of longitudinal effects were similar across genotypes in the two studies.

The flexible registration pipeline was tuned to reduce signal resampling and normalization. Because of this, individual-specific signal trends were preserved and quantitative comparisons across genotypes were potentiated. Use of hierarchical anatomical atlases - such as the DSURQE or the Allen Mouse Brain Atlas – enabled selection of brain sub-regions of varying granularity. In addition, the Allen Institute has published gene expression and functional connectivity data in the same space as the reference atlas (Lein et al., 2007). These data could be exploited in future studies of NPC mouse models if the Allen Mouse Brain atlas were aligned with the MRI study average (Yee et al., 2018; Fernandes et al., 2017).

Several brain sub-regions exhibited surprising hyperintensity in *Npc*^−*/*−^ mice, especially at later stages of disease. The cause of this is currently not well understood, but several features must be considered contributors to this effect. It is impossible to ignore the significant whole brain volume reduction in these mice. By nature of our imaging and processing workflow, partial volume effects become more prevalent as the segmented structure shrinks. Disentangling volume effects from the signal effects has proven to be challenging. Although Mn^2+^ dose was scaled to animal mass, *Npc*^−*/*−^ mice more aggressively lose brain volume compared to the magnitude of weight reduction. This may result in increased Mn^2+^ exposure to the brain in these animals compared to controls. Another challenging consequence of significant animal mass differences between control and *Npc*^−*/*−^ animals is systematic image acquisition parameter differences in (e.g. coil loading, automatic signal gain calculations, etc.). We attempted to correct for automatic global signal level adjustment apparent in online reconstructions by programmatically reversing automatic signal gain scaling before performing quantitative analyses *post hoc*. We also experimented with several forms of correction – including N3 bias field correction and population intensity normalization. However, we did not include these post-processing steps in any of the analyses presented in the manuscript. We have included the raw data used to perform these analyses in addition to the data normalized using population intensity normalization, N3 bias field correction, or both in Supplementary Data 1.

In addition to mass and volume effects, differences in cellular-level Mn^2+^ uptake mechanisms in the context of neurodegenerative disease have been reported (Saar and Koretsky, 2019). Hyperintensity in T1-weighted MEMRI has been attributed to astrogliosis in models of inflammation post ischemic stroke (Kawai et al., 2010) and late stage Parkinson’s disease (Olson et al., 2016), and to aberrant Ca^2+^ channel activity in models of multiple sclerosis (Boretius et al., 2008). Further mechanistic studies are required to determine the dominant uptake mechanism in this model of progressive NPC.

Our mice were on a Balb/cNctr background, which is the most widely studied model of progressive NPC. Given the natural heterogeneity in clinical disease presentation and progression, it may be prudent in the future to also study the longitudinal progression of other models of NPC. For example, the *Npc1*^*m1N*^ mutation on a C57BL/6J background has been shown to have more rapid and severe Purkinje cell degeneration than the animals used in this study (Parra et al., 2011). Conversely, the *Npc1*^*nmf164*^ model has been reported to be less aggressive and to more closely reflect the majority of the human alleles than the previously described null alleles (Maue et al., 2012). Assessments of behavioral deficits or regression- e.g. gait mapping, rotorod, or open field testing – have been performed on *Npc1*^*nmf164*^ and other NPC model mice (Maue et al., 2012; Xie et al., 2017), but correlations of imaging with behavior have been limited to a single timepoint (Maue et al., 2012). In future, correlations of longitudinal behavioral and noninvasive imaging data could help predict out-comes in studies of NPC model mice, and add another axis for therapeutic efficacy analysis.

Our cerebellar sub-region analyses suggest that the contrast reduction observed in late stage *Npc*^−*/*−^ mice cerebella are due to both volume and signal level changes of both the white matter and the cerebellar cortex. *Npc*^−*/*−^ mice have been shown to have significantly reduced numbers of Purkinje cells, whose cell bodies and dendrites make up a significant fraction of the cortex, while the white matter is composed mostly of Purkinje cell axons. These results motivate future studies of the cell-specific source of Mn^2+^ uptake in the cerebellum.

## Supplementary Material

1

2

3

Supplementary Table 1

Supplementary Table 3

Supplementary Table 2

Supplementary Figure 1

Supplementary Table 4

## Figures and Tables

**Table 1:** Summary of mice imaged in this study.

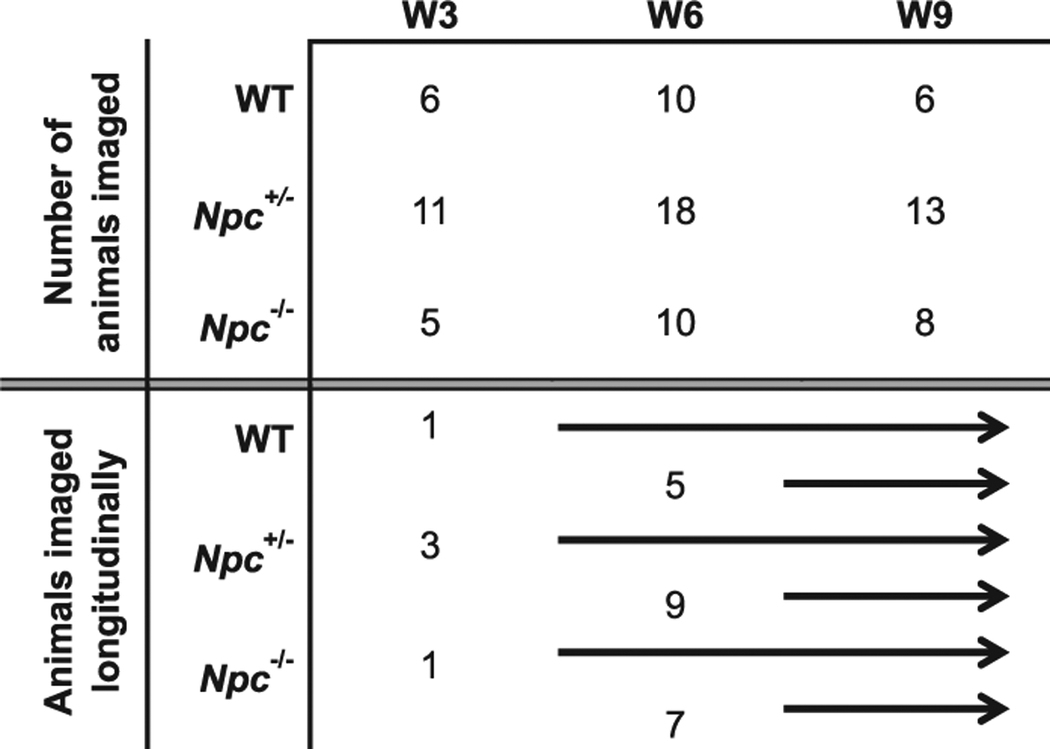

## References

[R1] Avants BB, Epstein CL, Grossman M, Gee JC, 2007 Symmetric Diffeomorphic Image Registration with Cross-Correlation: Evaluating Automated Labeling of Elderly and Neurodegenerative Brain. 10.1016/j.media.2007.06.004. PMC2276735 17659998

[R2] Bates D, Mächler M, Bolker BM, Walker SC, 2015 Fitting linear mixed-effects models using lme4. J. Stat. Software 67, 1–48.

[R3] Benjamini Y, Hochberg Y, 1995 Controlling the false discovery rate: a practical and powerful approach to multiple testing. J. R. Stat. Soc. Ser. B 57, 289–300.

[R4] Boretius S, , 2008 MRI of optic neuritis in a rat model. Neuroimage 41, 323–334. 18394926 10.1016/j.neuroimage.2008.02.021

[R5] Carstea ED, , 1997 Niemann-Pick C1 disease gene: homology to mediators of cholesterol homeostasis. Science 277, 228–231 (80-.). 9211849 10.1126/science.277.5323.228

[R6] Chakravarty MM, , 2013 Performing label-fusion-based segmentation using multiple automatically generated templates. Hum. Brain Mapp 34, 2635–2654. 22611030 10.1002/hbm.22092 PMC4896505

[R7] Dorr AE, Lerch JP, Spring S, Kabani N, Henkelman RM, 2008 High resolution three-dimensional brain atlas using an average magnetic resonance image of 40 adult C57Bl/6J mice. Neuroimage 42, 60–69. 18502665 10.1016/j.neuroimage.2008.03.037

[R8] Fernandes DJ, , 2017 Spatial gene expression analysis of neuroanatomical differences in mouse models. Neuroimage 163, 220–230. 28882630 10.1016/j.neuroimage.2017.08.065 PMC7097887

[R9] Friedel M, , 2014 Pydpiper: a Flexible Toolkit for Constructing Novel RegistrationPipelines. 10.3389/fninf.2014.00067. PMC4115634 25126069

[R10] Genovese CR, Lazar NA, Nichols T, 2002 Thresholding of statistical maps in functional neuroimaging using the false discovery rate. Neuroimage 15, 870–878. 11906227 10.1006/nimg.2001.1037

[R11] Kawai Y, , 2010 In vivo visualization of reactive gliosis using manganese-enhanced magnetic resonance imaging. Neuroimage 49, 3122–3131. 19909819 10.1016/j.neuroimage.2009.11.005 PMC5575780

[R12] Ko DC, , 2005 Cell-autonomous death of cerebellar purkinje neurons with autophagy in niemann-pick type C disease. PLoS Genet. 1, 0081–0095. 10.1371/journal.pgen.0010007 PMC1183526 16103921

[R13] Lein ES, , 2007 Genome-wide atlas of gene expression in the adult mouse brain. Nature 445, 168–176. 17151600 10.1038/nature05453

[R14] Lerch J, 2006 Voxel-wise Morphometry Using RMINC, vols. 1–8.

[R15] Lin Y-JJ, Koretsky AP, 1997 Manganese ion enhances T1-weighted MRI during brain activation: an approach to direct imaging of brain function. Magn. Reson. Med 38, 378–388. 9339438 10.1002/mrm.1910380305

[R16] Maue RA, , 2012 A novel mouse model of Niemann-Pick type C disease carrying a D1005G-Npc1 mutation comparable to commonly observed human mutations. Hum. Mol. Genet 21, 730–750. 22048958 10.1093/hmg/ddr505 PMC3263988

[R17] Morris MD, Bhuvaneswaran C, Shio H, Fowler S, 1982 Lysosome lipid storage disorder in NCTR-BALB/c mice. I. Description of the disease and genetics. Am. J. Pathol 108, 140–149. 6765731 PMC1916074

[R18] Olson KE, , 2016 Manganese-enhanced magnetic resonance imaging for detection of vasoactive intestinal peptide receptor 2 agonist therapy in a model of Parkinson’s disease. Neurotherapeutics 13, 635–646. 27329163 10.1007/s13311-016-0449-z PMC4965412

[R19] Papandreou A, Gissen P, 2016 Diagnostic workup and management of patients with suspected Niemann-Pick type C disease. Therapeut. Adv. Neurol. Disord 9, 216–229. 10.1177/1756285616635964 PMC4811014 27134677

[R20] Parra J, , 2011 Npc1 deficiency in the C57BL/6J genetic background enhances Niemann-Pick disease type C spleen pathology. Biochem. Biophys. Res. Commun 413, 400–406. 21910975 10.1016/j.bbrc.2011.08.096

[R21] Rallapalli H, , 2020 MEMRI-based imaging pipeline for guiding preclinical studies in mouse models of sporadic medulloblastoma. Magn. Reson. Med 83, 214–227. 31403226 10.1002/mrm.27904 PMC6778701

[R22] Richards K, , 2011 Segmentation of the mouse hippocampal formation in magnetic resonance images. Neuroimage 58, 732–740. 21704710 10.1016/j.neuroimage.2011.06.025

[R23] Saar G, Koretsky AP, 2019 Manganese enhanced MRI for use in studying neurodegenerative diseases. Front. Neural Circ 12. 10.3389/fncir.2018.00114 PMC6330305 30666190

[R24] Steadman PE, , 2014 Genetic effects on cerebellar structure across mouse models of autism using a magnetic resonance imaging atlas. Autism Res. 7, 124–137. 24151012 10.1002/aur.1344 PMC4418792

[R25] Suero-Abreu GA, , 2014 In vivo Mn-enhanced MRI for early tumor detection and growth rate analysis in a mouse medulloblastoma model. Neoplasia 16, 993–1006. 25499213 10.1016/j.neo.2014.10.001 PMC4309249

[R26] Szulc KU, , 2015 4D MEMRI atlas of neonatal FVB/N mouse brain development. Neuroimage 118, 49–62. 26037053 10.1016/j.neuroimage.2015.05.029 PMC4554969

[R27] Totenhagen JW, , 2012 In vivo assessment of neurodegeneration in niemann-pick type C mice by quantitative T2 mapping and diffusion tensor imaging. J. Magn. Reson. Imag 35, 528–536. 10.1002/jmri.22837 PMC3652272 22045516

[R28] Totenhagen JW, Bernstein A, Yoshimaru ES, Erickson RP, Trouard TP, 2017 Quantitative magnetic resonance imaging of brain atrophy in a mouse model of Niemann-Pick type C disease. PloS One 12, e0178179. 28542381 10.1371/journal.pone.0178179 PMC5443551

[R29] Trouard TP, Heidenreich RA, Seeger JF, Erickson RP, 2005 Diffusion tensor imaging in Niemann-Pick Type C disease. Pediatr. Neurol 33, 325–330. 16243219 10.1016/j.pediatrneurol.2005.05.004

[R30] Tukey JW, 1949 Comparing individual means in the analysis of variance. Biometrics 5,99. 18151955

[R31] Ullmann JFP, Watson C, Janke AL, Kurniawan ND, Reutens DC, 2013 A segmentation protocol and MRI atlas of the C57BL/6J mouse neocortex. Neuroimage 78, 196–203. 23587687 10.1016/j.neuroimage.2013.04.008

[R32] Wadghiri YZ, , 2004 Manganese-enhanced magnetic resonance imaging (MEMRI) of mouse brain development. NMR Biomed. 17, 613–619. 15761950 10.1002/nbm.932

[R33] Watanabe T, Natt O, Boretius S, Frahm J, Michaelis T, 2002 In vivo 3D MRI staining of mouse brain after subcutaneous application of MnCl2. Magn. Reson. Med 48, 852–859. 12418000 10.1002/mrm.10276

[R34] Watanabe T, Frahm J, Michaelis T, 2013 Cell layers and neuropil: contrast-enhancedMRI of mouse brain in vivo. NMR Biomed. 26, 1870–1878. 24142688 10.1002/nbm.3042

[R35] Whittaker DE, , 2017 Distinct cerebellar foliation anomalies in a CHD7 haploinsufficient mouse model of CHARGE syndrome. Am. J. Med. Genet. Part C Semin. Med. Genet 175. 10.1002/ajmg.c.31595 PMC5765394 29168327

[R36] Willett RT, , 2019 Cerebellar nuclei excitatory neurons regulate developmental scaling of presynaptic Purkinje cell number and organ growth. Elife 8. 10.7554/eLife.50617 PMC6890462 31742552

[R37] Xie C, Gong XM, Luo J, Li BL, Song BL, 2017 AAV9-NPC1 significantly ameliorates Purkinje cell death and behavioral abnormalities in mouse NPC disease. J. Lipid Res 58, 512–518. 28053186 10.1194/jlr.M071274 PMC5335581

[R38] Yee Y, , 2018 Structural covariance of brain region volumes is associated with both structural connectivity and transcriptomic similarity. Neuroimage 179, 357–372. 29782994 10.1016/j.neuroimage.2018.05.028

[R39] Yu X, Wadghiri YZ, Sanes DH, Turnbull DH, 2005 In vivo auditory brain mapping in mice with Mn-enhanced MRI. Nat. Neurosci 8, 961–968. 15924136 10.1038/nn1477 PMC2034206

