# Supplementary material for: Longitudinal MEMRI analysis of brain phenotypes in a mouse model of Niemann-Pick Type C disease
Source: Neuroimage. Author manuscript; Available in PMC 2020 Aug 24. (PMC7443857; doi:10.1016/j.neuroimage.2020.116894)
Supplement: 1 [file NIHMS1609340-supplement-1.zip › mmc1.html]

Longitudinal MEMRI analysis of brain phenotypes in a mouse model of Niemann-Pick Type C disease - Data Supplement 1


Code 

- Show All Code
- Hide All Code

# Longitudinal MEMRI analysis of brain phenotypes in a mouse model of Niemann-Pick Type C disease - Data Supplement 1

#### Hari Rallapalli

# For raw imaging data, please contact the corresponding author (Dr. Daniel H. Turnbull; daniel.turnbull@med.nyu.edu)

# Import data and tidy

## Import necessary libraries, define specialized functions

## Set figure and table settings

```
plottheme <- theme_minimal() + theme(strip.background = element_blank(), panel.grid.major = element_blank(), 
    panel.grid.minor = element_blank(), panel.background = element_rect(fill = "transparent", 
        colour = NA), plot.background = element_rect(fill = "transparent", colour = NA), 
    axis.line = element_line(size = 1.2, color = "black"), axis.ticks = element_line(size = 1.2, 
        color = "black"), axis.ticks.length = unit(0.25, "cm"), axis.text = element_text(face = "bold", 
        size = "13", color = "black"), axis.title = element_text(face = "bold", 
        size = "13", color = "black"), strip.text = element_text(face = "bold", 
        size = "13", color = "black"), legend.position = "right", legend.text = element_text(face = "bold", 
        size = "13", color = "black"), legend.title = element_text(face = "bold", 
        size = "13", color = "black"), legend.background = element_rect(fill = "transparent", 
        colour = NA))
```

## Import datasets

### The primary dataframe ‘gf’ uses ‘analysis.csv’ to fill the primary variables: mouse, genotype, time, sex, and mass. If one is interested in nonuniformity corrected and/or population intensity normalized data, they can be found in ‘uncorrected\_and\_corrected.csv’

### A table of the number of images at each timepoint, stratefied by genotype, is provided below.

```
gf <- read.csv("/media/sf_MINCVM/niemann-pick_mbm_complete/analysis.csv") %>% 
    filter(fwhm == 0.2) %>% select(-fwhm) %>% mutate(timepoint = str_extract(overall_xfm, 
    "W\\d+"), time = as.numeric(str_extract(timepoint, "\\d")), mouse = str_extract(overall_xfm, 
    "NPC\\d+_\\w\\d*")) %>% mutate(genotype = ifelse(str_detect(mouse, "_C\\d"), 
    "wt", ifelse(str_detect(mouse, "_H\\d"), "het", "mut")), genotype = fct_relevel(genotype, 
    "wt", "het", "mut")) %>% select(mouse, timepoint, time, genotype, label_file, 
    log_full_det, log_nlin_det, lsq6_file, nlin_file) %>% mutate(log_full_det = paste0("/media/sf_MINCVM/niemann-pick_mbm_complete/", 
    log_full_det), label_file = paste0("/media/sf_MINCVM/niemann-pick_mbm_complete/", 
    label_file), log_nlin_det = paste0("/media/sf_MINCVM/niemann-pick_mbm_complete/", 
    log_nlin_det), lsq6_file = paste0("/media/sf_MINCVM/niemann-pick_mbm_complete/", 
    lsq6_file), nlin_file = paste0("/media/sf_MINCVM/niemann-pick_mbm_complete/", 
    nlin_file)) %>% mutate(time_corrected = I(time - 3))

anatVol <- mincArray(mincGetVolume("/media/sf_MINCVM/niemann-pick_mbm_complete/NP-reg_nlin/NP-reg-nlin-3.mnc"))
maskVol <- "/media/sf_MINCVM/niemann-pick_mbm_complete/npc_nlin_mask_new.mnc"

tmpf = read.csv("/media/sf_MINCVM/niemann-pick_mbm_complete/2018-11-hari-nuc-data/uncorrected_and_corrected.csv") %>% 
    select(uncorrected_lsq6_file, nuc_file) %>% mutate(uncorrected_lsq6_file = paste0("/media/sf_MINCVM/niemann-pick_mbm_complete/2018-11-hari-nuc-data/", 
    uncorrected_lsq6_file), nuc_file = paste0("/media/sf_MINCVM/niemann-pick_mbm_complete/2018-11-hari-nuc-data/", 
    nuc_file))

gf$uncorrected_lsq6_file = tmpf$uncorrected_lsq6_file
gf$nuc_file = tmpf$nuc_file

rm(tmpf)

tmpf = read.csv("/media/sf_MINCVM/niemann-pick_mbm_complete/2018-11-hari-nuc-data/nuc_and_uncorrected_nlin.csv") %>% 
    select(uncorrected_nlin_file, nuc_nlin_file) %>% mutate(uncorrected_nlin_file = paste0("/media/sf_MINCVM/niemann-pick_mbm_complete/2018-11-hari-nuc-data/", 
    uncorrected_nlin_file), nuc_nlin_file = paste0("/media/sf_MINCVM/niemann-pick_mbm_complete/2018-11-hari-nuc-data/", 
    nuc_nlin_file))

gf$uncorrected_nlin_file = tmpf$uncorrected_nlin_file
gf$nuc_nlin_file = tmpf$nuc_nlin_file


rm(tmpf)

tmpf = read.csv("/media/sf_MINCVM/niemann-pick_mbm_complete/DSURQE_output/filenames.csv", 
    header = FALSE)

gf$label_file_DSURQE = tmpf$V1

rm(tmpf)

tmpf = read.csv("/media/sf_MINCVM/niemann-pick_mbm_complete/NPC_AnimalMass.csv", 
    header = TRUE)

gf$mass = tmpf$mass
gf$sex = tmpf$sex

rm(tmpf)

labeller = c(wt = "WT", het = bquote(bolditalic("Npc"^"+/-")), mut = bquote(bolditalic("Npc"^"-/-")))

write.csv(gf, "/media/sf_MINCVM/niemann-pick_mbm_complete/HR_NPC_PAPER_ANALYSIS_files/NPC_PAPER_ANALYSIS.csv")

gf %>% group_by(time, genotype) %>% summarize(n = n_distinct(mouse)) %>% spread(genotype, 
    n) %>% knitr::kable()
```

| time | wt | het | mut |
| --- | --- | --- | --- |
| 3 | 6 | 11 | 5 |
| 6 | 10 | 18 | 10 |
| 9 | 6 | 13 | 8 |

# Extract segmented volumes and signal information

## Set atlas definitions and create an anatomical hierarchy based on ABI atlas.

```
defs.DSURQE = "/media/sf_MINCVM/Dorr_2008_Steadman_2013_Ullmann_2013_Richards_2011_Qiu_2016_Egan_2015_40micron/mappings/DSURQE_40micron_R_mapping.csv"
atlas.DSURQE = "/media/sf_MINCVM/niemann-pick_mbm_complete/NP-reg-nlin-3-DSURQE_voted.mnc"

AllenDefs <- "/media/sf_MINCVM/Allen_Brain/Allen_hierarchy_definitions.json"
hdefs <- makeMICeDefsHierachical(defs.DSURQE, AllenDefs)
```

```
allvols.DSURQE <- anatGetAll(gf$log_full_det, defs = defs.DSURQE, method = "jacobians", 
    atlas = atlas.DSURQE, parallel = c("local", 8))
allsums.DSURQE <- anatGetAll(gf$uncorrected_lsq6_file, defs = defs.DSURQE, method = "sums", 
    atlas = atlas.DSURQE, parallel = c("local", 8))

combvols.DSURQE <- anatCombineStructures(allvols.DSURQE, defs = defs.DSURQE)
combsums.DSURQE <- anatCombineStructures(allsums.DSURQE, defs = defs.DSURQE)

combmeans.DSURQE <- combsums.DSURQE/combvols.DSURQE

hanat <- addVolumesToHierarchy(hdefs, allvols.DSURQE)
hsums <- addVolumesToHierarchy(hdefs, allsums.DSURQE)
```

## Pull region signal and volume information from the hierarchy

```
WholeBrain.Volume = rowSums(combvols.DSURQE)
WholeBrain.Signal = rowSums(combsums.DSURQE)
WholeBrain.Mean = WholeBrain.Signal/WholeBrain.Volume

cbdf <- gf %>% mutate(
CB.Volume = FindNode(hanat,"Cerebellum")$volumes + FindNode(hanat,"arbor vitae")$volumes,
CB.Signal = FindNode(hsums,"Cerebellum")$volumes + FindNode(hsums,"arbor vitae")$volumes,
CB.Mean = CB.Signal/CB.Volume,

CB.Cortex.Signal = FindNode(hsums,"Cerebellar cortex")$volumes,
CB.Cortex.Volume = FindNode(hanat,"Cerebellar cortex")$volumes,
CB.Cortex.Mean = CB.Cortex.Signal/CB.Cortex.Volume,

CB.Nuclei.Signal = FindNode(hsums,"Cerebellar nuclei")$volumes,
CB.Nuclei.Volume = FindNode(hanat,"Cerebellar nuclei")$volumes,
CB.Nuclei.Mean = CB.Nuclei.Signal/CB.Nuclei.Volume,

CB.WhiteMatter.Signal = FindNode(hsums,"arbor vitae")$volumes,
CB.WhiteMatter.Volume = FindNode(hanat,"arbor vitae")$volumes,
CB.WhiteMatter.Mean = CB.WhiteMatter.Signal/CB.WhiteMatter.Volume,

CB.Vermis.Signal = FindNode(hsums,"Vermal regions")$volumes,
CB.Vermis.Volume = FindNode(hanat,"Vermal regions")$volumes,
CB.Vermis.Mean = CB.Vermis.Signal/CB.Vermis.Volume,

CB.Hemisphere.Signal = FindNode(hsums,"Hemispheric regions")$volumes,
CB.Hemisphere.Volume = FindNode(hanat,"Hemispheric regions")$volumes,
CB.Hemisphere.Mean = CB.Hemisphere.Signal/CB.Hemisphere.Volume,

OlfactoryBulb.Signal = FindNode(hsums,"Main olfactory bulb")$volumes,
OlfactoryBulb.Volume = FindNode(hanat,"Main olfactory bulb")$volumes,
OlfactoryBulb.Mean = OlfactoryBulb.Signal/OlfactoryBulb.Volume,

Cortex.Signal = FindNode(hsums,"Isocortex")$volumes,
Cortex.Volume = FindNode(hanat,"Isocortex")$volumes,
Cortex.Mean = Cortex.Signal/Cortex.Volume,

Thalamus.Signal = FindNode(hsums,"Thalamus")$volumes,
Thalamus.Volume = FindNode(hanat,"Thalamus")$volumes,
Thalamus.Mean = Thalamus.Signal/Thalamus.Volume,

Hypothalamus.Signal = FindNode(hsums,"Hypothalamus")$volumes,
Hypothalamus.Volume = FindNode(hanat,"Hypothalamus")$volumes,
Hypothalamus.Mean = Hypothalamus.Signal/Hypothalamus.Volume,

Hippocampus.Signal = FindNode(hsums,"Hippocampal formation")$volumes,
Hippocampus.Volume = FindNode(hanat,"Hippocampal formation")$volumes,
Hippocampus.Mean = Hippocampus.Signal/Hippocampus.Volume,

Midbrain.Signal = FindNode(hsums,"Midbrain")$volumes,
Midbrain.Volume = FindNode(hanat,"Midbrain")$volumes,
Midbrain.Mean = Midbrain.Signal/Midbrain.Volume,

Hindbrain.Signal = FindNode(hsums,"Hindbrain")$volumes,
Hindbrain.Volume = FindNode(hanat,"Hindbrain")$volumes,
Hindbrain.Mean = Hindbrain.Signal/Hindbrain.Volume,

WholeBrain.Volume = rowSums(combvols.DSURQE),
WholeBrain.Signal = rowSums(combsums.DSURQE),
WholeBrain.Mean = WholeBrain.Signal/WholeBrain.Volume)


CB.Volume = FindNode(hanat,"Cerebellum")$volumes + FindNode(hanat,"arbor vitae")$volumes
CB.Signal = FindNode(hsums,"Cerebellum")$volumes + FindNode(hsums,"arbor vitae")$volumes
CB.Mean = CB.Signal/CB.Volume

CB.Cortex.Signal = FindNode(hsums,"Cerebellar cortex")$volumes
CB.Cortex.Volume = FindNode(hanat,"Cerebellar cortex")$volumes
CB.Cortex.Mean = CB.Cortex.Signal/CB.Cortex.Volume

CB.Nuclei.Signal = FindNode(hsums,"Cerebellar nuclei")$volumes
CB.Nuclei.Volume = FindNode(hanat,"Cerebellar nuclei")$volumes
CB.Nuclei.Mean = CB.Nuclei.Signal/CB.Nuclei.Volume

CB.WhiteMatter.Signal = FindNode(hsums,"arbor vitae")$volumes
CB.WhiteMatter.Volume = FindNode(hanat,"arbor vitae")$volumes
CB.WhiteMatter.Mean = CB.WhiteMatter.Signal/CB.WhiteMatter.Volume

CB.Vermis.Signal = FindNode(hsums,"Vermal regions")$volumes
CB.Vermis.Volume = FindNode(hanat,"Vermal regions")$volumes
CB.Vermis.Mean = CB.Vermis.Signal/CB.Vermis.Volume

CB.Hemisphere.Signal = FindNode(hsums,"Hemispheric regions")$volumes
CB.Hemisphere.Volume = FindNode(hanat,"Hemispheric regions")$volumes
CB.Hemisphere.Mean = CB.Hemisphere.Signal/CB.Hemisphere.Volume

OlfactoryBulb.Signal = FindNode(hsums,"Main olfactory bulb")$volumes
OlfactoryBulb.Volume = FindNode(hanat,"Main olfactory bulb")$volumes
OlfactoryBulb.Mean = OlfactoryBulb.Signal/OlfactoryBulb.Volume

Cortex.Signal = FindNode(hsums,"Isocortex")$volumes
Cortex.Volume = FindNode(hanat,"Isocortex")$volumes
Cortex.Mean = Cortex.Signal/Cortex.Volume

Thalamus.Signal = FindNode(hsums,"Thalamus")$volumes
Thalamus.Volume = FindNode(hanat,"Thalamus")$volumes
Thalamus.Mean = Thalamus.Signal/Thalamus.Volume

Hypothalamus.Signal = FindNode(hsums,"Hypothalamus")$volumes
Hypothalamus.Volume = FindNode(hanat,"Hypothalamus")$volumes
Hypothalamus.Mean = Hypothalamus.Signal/Hypothalamus.Volume

Hippocampus.Signal = FindNode(hsums,"Hippocampal formation")$volumes
Hippocampus.Volume = FindNode(hanat,"Hippocampal formation")$volumes
Hippocampus.Mean = Hippocampus.Signal/Hippocampus.Volume

Midbrain.Signal = FindNode(hsums,"Midbrain")$volumes
Midbrain.Volume = FindNode(hanat,"Midbrain")$volumes
Midbrain.Mean = Midbrain.Signal/Midbrain.Volume

Hindbrain.Signal = FindNode(hsums,"Hindbrain")$volumes
Hindbrain.Volume = FindNode(hanat,"Hindbrain")$volumes
Hindbrain.Mean = Hindbrain.Signal/Hindbrain.Volume

WholeBrain.Volume = rowSums(combvols.DSURQE)
WholeBrain.Signal = rowSums(combsums.DSURQE)
WholeBrain.Mean = WholeBrain.Signal/WholeBrain.Volume
```

## Additional sanity check for timepoint-specific analyses

```
dothis = FALSE
if (dothis) {
    vs_W3 <- mincLmer(log_full_det ~ I(time - 3) * genotype + (1 | mouse), gf, 
        mask = maskVol, parallel = c("local", 8))
    vs_W3 <- mincLmerEstimateDF(vs_W3)
    qvs_W3 <- mincFDR(vs_W3, mask = maskVol)
    thresholds(qvs_W3) %>% knitr::kable()
    
    vs_W6 <- mincLmer(log_full_det ~ I(time - 6) * genotype + (1 | mouse), gf, 
        mask = maskVol, parallel = c("local", 8))
    vs_W6 <- mincLmerEstimateDF(vs_W6)
    qvs_W6 <- mincFDR(vs_W6, mask = maskVol)
    thresholds(qvs_W6) %>% knitr::kable()
    
    vs_W9 <- mincLmer(log_full_det ~ I(time - 9) * genotype + (1 | mouse), gf, 
        mask = maskVol, parallel = c("local", 8))
    vs_W9 <- mincLmerEstimateDF(vs_W9)
    qvs_W9 <- mincFDR(vs_W9, mask = maskVol)
    thresholds(qvs_W9) %>% knitr::kable()
    save(vs_W3, qvs_W3, vs_W6, qvs_W6, vs_W9, qvs_W9, file = "NPC_modeling_vs.RData")
}
load("NPC_modeling_vs.RData")
```

# Linear modeling of volume trends over time

```
Cerebellumlm <- cbdf %>% lmer(CB.Volume ~ genotype * timepoint + (1 | mouse), 
    data = .)


Cerebellum.Volume.Pairwise <- emmeans(Cerebellumlm, ~genotype * timepoint)
pairs(Cerebellum.Volume.Pairwise)
```

```
##  contrast        estimate    SE   df t.ratio p.value
##  wt,W3 - het,W3     0.567 0.781 76.9   0.727 0.9982 
##  wt,W3 - mut,W3     4.399 0.933 76.8   4.716 0.0003 
##  wt,W3 - wt,W6     -4.921 0.773 77.7  -6.363 <.0001 
##  wt,W3 - het,W6    -5.400 0.730 75.5  -7.395 <.0001 
##  wt,W3 - mut,W6     0.689 0.802 74.6   0.859 0.9944 
##  wt,W3 - wt,W9     -6.065 0.840 73.5  -7.222 <.0001 
##  wt,W3 - het,W9    -6.459 0.757 77.1  -8.529 <.0001 
##  wt,W3 - mut,W9     5.242 0.832 76.5   6.302 <.0001 
##  het,W3 - mut,W3    3.832 0.827 77.4   4.634 0.0005 
##  het,W3 - wt,W6    -5.488 0.676 75.4  -8.115 <.0001 
##  het,W3 - het,W6   -5.968 0.553 71.3 -10.795 <.0001 
##  het,W3 - mut,W6    0.122 0.676 75.3   0.180 1.0000 
##  het,W3 - wt,W9    -6.632 0.764 77.9  -8.675 <.0001 
##  het,W3 - het,W9   -7.026 0.579 66.3 -12.126 <.0001 
##  het,W3 - mut,W9    4.675 0.711 77.3   6.576 <.0001 
##  mut,W3 - wt,W6    -9.320 0.847 75.8 -10.998 <.0001 
##  mut,W3 - het,W6   -9.800 0.780 76.5 -12.571 <.0001 
##  mut,W3 - mut,W6   -3.710 0.814 76.9  -4.555 0.0006 
##  mut,W3 - wt,W9   -10.464 0.919 78.0 -11.382 <.0001 
##  mut,W3 - het,W9  -10.858 0.805 77.6 -13.490 <.0001 
##  mut,W3 - mut,W9    0.843 0.838 75.3   1.006 0.9842 
##  wt,W6 - het,W6    -0.479 0.618 72.2  -0.776 0.9972 
##  wt,W6 - mut,W6     5.610 0.701 71.5   7.999 <.0001 
##  wt,W6 - wt,W9     -1.144 0.620 34.6  -1.844 0.6538 
##  wt,W6 - het,W9    -1.538 0.649 75.7  -2.369 0.3162 
##  wt,W6 - mut,W9    10.163 0.735 74.9  13.832 <.0001 
##  het,W6 - mut,W6    6.089 0.618 72.0   9.859 <.0001 
##  het,W6 - wt,W9    -0.664 0.713 78.0  -0.932 0.9904 
##  het,W6 - het,W9   -1.058 0.427 32.6  -2.476 0.2799 
##  het,W6 - mut,W9   10.642 0.655 75.9  16.241 <.0001 
##  mut,W6 - wt,W9    -6.754 0.787 77.7  -8.584 <.0001 
##  mut,W6 - het,W9   -7.148 0.649 75.6 -11.008 <.0001 
##  mut,W6 - mut,W9    4.553 0.550 31.4   8.279 <.0001 
##  wt,W9 - het,W9    -0.394 0.741 77.7  -0.532 0.9998 
##  wt,W9 - mut,W9    11.307 0.817 78.0  13.845 <.0001 
##  het,W9 - mut,W9   11.701 0.685 77.6  17.075 <.0001 
## 
## Degrees-of-freedom method: kenward-roger 
## P value adjustment: tukey method for comparing a family of 9 estimates
```

```
summary(Cerebellumlm)
```

```
## Linear mixed model fit by REML. t-tests use Satterthwaite's method [
## lmerModLmerTest]
## Formula: CB.Volume ~ genotype * timepoint + (1 | mouse)
##    Data: .
## 
## REML criterion at convergence: 304.3
## 
## Scaled residuals: 
##     Min      1Q  Median      3Q     Max 
## -2.5313 -0.4022  0.0696  0.4116  1.9694 
## 
## Random effects:
##  Groups   Name        Variance Std.Dev.
##  mouse    (Intercept) 1.235    1.111   
##  Residual             1.264    1.124   
## Number of obs: 87, groups:  mouse, 55
## 
## Fixed effects:
##                         Estimate Std. Error      df t value Pr(>|t|)    
## (Intercept)              41.4914     0.6246 76.3719  66.430  < 2e-16 ***
## genotypehet              -0.5671     0.7725 77.0222  -0.734    0.465    
## genotypemut              -4.3991     0.9229 76.9140  -4.766 8.69e-06 ***
## timepointW6               4.9211     0.7610 77.7227   6.467 8.09e-09 ***
## timepointW9               6.0646     0.8255 73.9935   7.346 2.21e-10 ***
## genotypehet:timepointW6   1.0465     0.9344 76.5845   1.120    0.266    
## genotypemut:timepointW6  -1.2110     1.1045 77.3954  -1.096    0.276    
## genotypehet:timepointW9   0.9613     1.0023 72.1602   0.959    0.341    
## genotypemut:timepointW9  -6.9074     1.1660 74.8451  -5.924 8.94e-08 ***
## ---
## Signif. codes:  0 '***' 0.001 '**' 0.01 '*' 0.05 '.' 0.1 ' ' 1
## 
## Correlation of Fixed Effects:
##             (Intr) gntyph gntypm tmpnW6 tmpnW9 gntyph:W6 gntypm:W6
## genotypehet -0.809                                                
## genotypemut -0.677  0.547                                         
## timepointW6 -0.764  0.618  0.517                                  
## timepointW9 -0.686  0.555  0.464  0.702                           
## gntypht:tW6  0.622 -0.757 -0.421 -0.814 -0.572                    
## gntypmt:tW6  0.526 -0.425 -0.777 -0.689 -0.484  0.561             
## gntypht:tW9  0.565 -0.688 -0.383 -0.579 -0.824  0.704     0.399   
## gntypmt:tW9  0.486 -0.393 -0.724 -0.497 -0.708  0.405     0.738   
##             gntyph:W9
## genotypehet          
## genotypemut          
## timepointW6          
## timepointW9          
## gntypht:tW6          
## gntypmt:tW6          
## gntypht:tW9          
## gntypmt:tW9  0.583
```

```
OlfactoryBulblm <- cbdf %>% lmer(OlfactoryBulb.Volume ~ genotype * timepoint + 
    (1 | mouse), data = .)

OlfactoryBulb.Volume.Pairwise <- emmeans(OlfactoryBulblm, ~genotype * timepoint)
pairs(OlfactoryBulb.Volume.Pairwise)
```

```
##  contrast        estimate    SE   df t.ratio p.value
##  wt,W3 - het,W3    0.4945 0.440 76.8   1.124 0.9688 
##  wt,W3 - mut,W3    1.8361 0.526 76.7   3.490 0.0216 
##  wt,W3 - wt,W6    -2.3245 0.433 77.4  -5.366 <.0001 
##  wt,W3 - het,W6   -2.4849 0.413 75.2  -6.020 <.0001 
##  wt,W3 - mut,W6    0.7985 0.454 74.1   1.758 0.7085 
##  wt,W3 - wt,W9    -3.2771 0.467 72.4  -7.024 <.0001 
##  wt,W3 - het,W9   -3.3036 0.427 76.9  -7.744 <.0001 
##  wt,W3 - mut,W9    1.2375 0.469 76.2   2.638 0.1888 
##  het,W3 - mut,W3   1.3416 0.466 77.4   2.882 0.1093 
##  het,W3 - wt,W6   -2.8190 0.382 74.9  -7.376 <.0001 
##  het,W3 - het,W6  -2.9794 0.306 69.4  -9.722 <.0001 
##  het,W3 - mut,W6   0.3040 0.382 74.8   0.795 0.9967 
##  het,W3 - wt,W9   -3.7716 0.428 77.9  -8.818 <.0001 
##  het,W3 - het,W9  -3.7981 0.320 64.0 -11.885 <.0001 
##  het,W3 - mut,W9   0.7430 0.400 77.1   1.857 0.6445 
##  mut,W3 - wt,W6   -4.1606 0.479 75.5  -8.691 <.0001 
##  mut,W3 - het,W6  -4.3210 0.440 76.4  -9.825 <.0001 
##  mut,W3 - mut,W6  -1.0376 0.455 76.2  -2.280 0.3670 
##  mut,W3 - wt,W9   -5.1132 0.516 78.0  -9.913 <.0001 
##  mut,W3 - het,W9  -5.1397 0.453 77.5 -11.351 <.0001 
##  mut,W3 - mut,W9  -0.5986 0.467 74.3  -1.282 0.9334 
##  wt,W6 - het,W6   -0.1604 0.350 71.2  -0.458 0.9999 
##  wt,W6 - mut,W6    3.1230 0.398 70.4   7.841 <.0001 
##  wt,W6 - wt,W9    -0.9526 0.332 33.3  -2.868 0.1337 
##  wt,W6 - het,W9   -0.9791 0.367 75.1  -2.671 0.1763 
##  wt,W6 - mut,W9    3.5620 0.415 74.2   8.574 <.0001 
##  het,W6 - mut,W6   3.2834 0.351 70.9   9.367 <.0001 
##  het,W6 - wt,W9   -0.7922 0.400 78.0  -1.983 0.5599 
##  het,W6 - het,W9  -0.8187 0.229 31.6  -3.582 0.0271 
##  het,W6 - mut,W9   3.7224 0.370 75.3  10.064 <.0001 
##  mut,W6 - wt,W9   -4.0756 0.442 77.4  -9.218 <.0001 
##  mut,W6 - het,W9  -4.1021 0.367 74.9 -11.187 <.0001 
##  mut,W6 - mut,W9   0.4390 0.294 30.6   1.495 0.8493 
##  wt,W9 - het,W9   -0.0265 0.414 77.8  -0.064 1.0000 
##  wt,W9 - mut,W9    4.5146 0.458 78.0   9.865 <.0001 
##  het,W9 - mut,W9   4.5411 0.385 77.3  11.788 <.0001 
## 
## Degrees-of-freedom method: kenward-roger 
## P value adjustment: tukey method for comparing a family of 9 estimates
```

```
summary(OlfactoryBulblm)
```

```
## Linear mixed model fit by REML. t-tests use Satterthwaite's method [
## lmerModLmerTest]
## Formula: OlfactoryBulb.Volume ~ genotype * timepoint + (1 | mouse)
##    Data: .
## 
## REML criterion at convergence: 214
## 
## Scaled residuals: 
##      Min       1Q   Median       3Q      Max 
## -1.97828 -0.42865 -0.07384  0.41950  1.89743 
## 
## Random effects:
##  Groups   Name        Variance Std.Dev.
##  mouse    (Intercept) 0.4552   0.6747  
##  Residual             0.3583   0.5986  
## Number of obs: 87, groups:  mouse, 55
## 
## Fixed effects:
##                         Estimate Std. Error      df t value Pr(>|t|)    
## (Intercept)              19.3069     0.3524 76.0793  54.789  < 2e-16 ***
## genotypehet              -0.4945     0.4352 76.8301  -1.136 0.259396    
## genotypemut              -1.8361     0.5201 76.7515  -3.530 0.000705 ***
## timepointW6               2.3245     0.4254 77.4108   5.464 5.46e-07 ***
## timepointW9               3.2771     0.4581 72.7378   7.154 5.49e-10 ***
## genotypehet:timepointW6   0.6549     0.5206 75.7689   1.258 0.212248    
## genotypemut:timepointW6  -1.2869     0.6166 76.8915  -2.087 0.040191 *  
## genotypehet:timepointW9   0.5210     0.5549 70.4515   0.939 0.350982    
## genotypemut:timepointW9  -2.6785     0.6478 73.6705  -4.135 9.30e-05 ***
## ---
## Signif. codes:  0 '***' 0.001 '**' 0.01 '*' 0.05 '.' 0.1 ' ' 1
## 
## Correlation of Fixed Effects:
##             (Intr) gntyph gntypm tmpnW6 tmpnW9 gntyph:W6 gntypm:W6
## genotypehet -0.810                                                
## genotypemut -0.678  0.549                                         
## timepointW6 -0.756  0.612  0.513                                  
## timepointW9 -0.683  0.553  0.463  0.724                           
## gntypht:tW6  0.618 -0.748 -0.419 -0.817 -0.592                    
## gntypmt:tW6  0.522 -0.423 -0.770 -0.690 -0.499  0.564             
## gntypht:tW9  0.564 -0.683 -0.382 -0.598 -0.826  0.725     0.412   
## gntypmt:tW9  0.483 -0.391 -0.719 -0.512 -0.707  0.418     0.758   
##             gntyph:W9
## genotypehet          
## genotypemut          
## timepointW6          
## timepointW9          
## gntypht:tW6          
## gntypmt:tW6          
## gntypht:tW9          
## gntypmt:tW9  0.584
```

```
Cortexlm <- cbdf %>% lmer(Cortex.Volume ~ genotype * timepoint + (1 | mouse), 
    data = .)

Cortex.Volume.Pairwise <- emmeans(Cortexlm, ~genotype * timepoint)
pairs(Cortex.Volume.Pairwise)
```

```
##  contrast        estimate    SE   df t.ratio p.value
##  wt,W3 - het,W3    1.7591 1.488 77.5  1.182  0.9579 
##  wt,W3 - mut,W3    5.5288 1.776 77.4  3.113  0.0615 
##  wt,W3 - wt,W6    -7.2354 1.491 77.8 -4.853  0.0002 
##  wt,W3 - het,W6   -7.3440 1.385 76.8 -5.304  <.0001 
##  wt,W3 - mut,W6    2.1217 1.518 76.4  1.398  0.8952 
##  wt,W3 - wt,W9    -7.1667 1.650 75.0 -4.344  0.0014 
##  wt,W3 - het,W9   -6.9130 1.447 77.6 -4.779  0.0003 
##  wt,W3 - mut,W9   11.0282 1.584 77.3  6.961  <.0001 
##  het,W3 - mut,W3   3.7697 1.580 77.7  2.386  0.3065 
##  het,W3 - wt,W6   -8.9944 1.283 76.8 -7.011  <.0001 
##  het,W3 - het,W6  -9.1031 1.088 73.9 -8.365  <.0001 
##  het,W3 - mut,W6   0.3627 1.283 76.8  0.283  1.0000 
##  het,W3 - wt,W9   -8.9258 1.482 77.9 -6.023  <.0001 
##  het,W3 - het,W9  -8.6720 1.154 70.2 -7.513  <.0001 
##  het,W3 - mut,W9   9.2691 1.361 77.8  6.810  <.0001 
##  mut,W3 - wt,W6  -12.7641 1.608 77.0 -7.937  <.0001 
##  mut,W3 - het,W6 -12.8728 1.483 77.3 -8.678  <.0001 
##  mut,W3 - mut,W6  -3.4070 1.578 77.3 -2.159  0.4421 
##  mut,W3 - wt,W9  -12.6955 1.771 78.0 -7.168  <.0001 
##  mut,W3 - het,W9 -12.4418 1.541 77.8 -8.072  <.0001 
##  mut,W3 - mut,W9   5.4994 1.635 76.3  3.364  0.0312 
##  wt,W6 - het,W6   -0.1087 1.162 75.1 -0.094  1.0000 
##  wt,W6 - mut,W6    9.3571 1.317 74.8  7.102  <.0001 
##  wt,W6 - wt,W9     0.0686 1.325 38.6  0.052  1.0000 
##  wt,W6 - het,W9    0.3224 1.235 77.1  0.261  1.0000 
##  wt,W6 - mut,W9   18.2636 1.394 76.7 13.104  <.0001 
##  het,W6 - mut,W6   9.4658 1.162 75.1  8.149  <.0001 
##  het,W6 - wt,W9    0.1773 1.378 78.0  0.129  1.0000 
##  het,W6 - het,W9   0.4310 0.919 35.6  0.469  0.9999 
##  het,W6 - mut,W9  18.3722 1.247 77.2 14.728  <.0001 
##  mut,W6 - wt,W9   -9.2885 1.512 77.9 -6.144  <.0001 
##  mut,W6 - het,W9  -9.0347 1.235 77.0 -7.316  <.0001 
##  mut,W6 - mut,W9   8.9065 1.187 34.0  7.501  <.0001 
##  wt,W9 - het,W9    0.2537 1.441 77.7  0.176  1.0000 
##  wt,W9 - mut,W9   18.1949 1.579 78.0 11.524  <.0001 
##  het,W9 - mut,W9  17.9412 1.316 77.9 13.633  <.0001 
## 
## Degrees-of-freedom method: kenward-roger 
## P value adjustment: tukey method for comparing a family of 9 estimates
```

```
summary(Cortexlm)
```

```
## Linear mixed model fit by REML. t-tests use Satterthwaite's method [
## lmerModLmerTest]
## Formula: Cortex.Volume ~ genotype * timepoint + (1 | mouse)
##    Data: .
## 
## REML criterion at convergence: 406.7
## 
## Scaled residuals: 
##     Min      1Q  Median      3Q     Max 
## -2.0564 -0.4801 -0.0291  0.4396  2.1212 
## 
## Random effects:
##  Groups   Name        Variance Std.Dev.
##  mouse    (Intercept) 2.678    1.637   
##  Residual             6.007    2.451   
## Number of obs: 87, groups:  mouse, 55
## 
## Fixed effects:
##                         Estimate Std. Error      df t value Pr(>|t|)    
## (Intercept)               86.955      1.189  77.233  73.150  < 2e-16 ***
## genotypehet               -1.759      1.475  77.567  -1.193  0.23658    
## genotypemut               -5.529      1.761  77.481  -3.140  0.00239 ** 
## timepointW6                7.235      1.474  77.822   4.907 4.97e-06 ***
## timepointW9                7.167      1.626  75.381   4.408 3.41e-05 ***
## genotypehet:timepointW6    1.868      1.823  77.123   1.025  0.30878    
## genotypemut:timepointW6   -3.828      2.145  77.644  -1.785  0.07825 .  
## genotypehet:timepointW9    1.505      1.983  74.180   0.759  0.45024    
## genotypemut:timepointW9  -12.666      2.290  75.994  -5.531 4.32e-07 ***
## ---
## Signif. codes:  0 '***' 0.001 '**' 0.01 '*' 0.05 '.' 0.1 ' ' 1
## 
## Correlation of Fixed Effects:
##             (Intr) gntyph gntypm tmpnW6 tmpnW9 gntyph:W6 gntypm:W6
## genotypehet -0.806                                                
## genotypemut -0.675  0.544                                         
## timepointW6 -0.778  0.627  0.525                                  
## timepointW9 -0.693  0.559  0.468  0.645                           
## gntypht:tW6  0.629 -0.774 -0.425 -0.809 -0.522                    
## gntypmt:tW6  0.535 -0.431 -0.792 -0.687 -0.443  0.556             
## gntypht:tW9  0.568 -0.699 -0.384 -0.529 -0.820  0.650     0.364   
## gntypmt:tW9  0.492 -0.397 -0.733 -0.458 -0.710  0.370     0.684   
##             gntyph:W9
## genotypehet          
## genotypemut          
## timepointW6          
## timepointW9          
## gntypht:tW6          
## gntypmt:tW6          
## gntypht:tW9          
## gntypmt:tW9  0.582
```

```
Hippocampuslm <- cbdf %>% lmer(Hippocampus.Volume ~ genotype * timepoint + (1 | 
    mouse), data = .)

Hippocampus.Volume.Pairwise <- emmeans(Hippocampuslm, ~genotype * timepoint)
pairs(Hippocampus.Volume.Pairwise)
```

```
##  contrast        estimate    SE   df t.ratio p.value
##  wt,W3 - het,W3   0.64283 0.534 76.7  1.203  0.9535 
##  wt,W3 - mut,W3   2.21638 0.639 76.7  3.468  0.0230 
##  wt,W3 - wt,W6   -2.17303 0.524 77.1 -4.149  0.0026 
##  wt,W3 - het,W6  -2.16946 0.502 75.0 -4.321  0.0015 
##  wt,W3 - mut,W6   1.36122 0.553 73.9  2.462  0.2674 
##  wt,W3 - wt,W9   -2.72905 0.561 71.7 -4.860  0.0002 
##  wt,W3 - het,W9  -2.88373 0.518 76.8 -5.568  <.0001 
##  wt,W3 - mut,W9   2.94564 0.570 76.0  5.167  0.0001 
##  het,W3 - mut,W3  1.57356 0.565 77.4  2.786  0.1365 
##  het,W3 - wt,W6  -2.81586 0.465 74.7 -6.057  <.0001 
##  het,W3 - het,W6 -2.81229 0.368 68.0 -7.638  <.0001 
##  het,W3 - mut,W6  0.71840 0.465 74.5  1.545  0.8305 
##  het,W3 - wt,W9  -3.37188 0.517 77.9 -6.519  <.0001 
##  het,W3 - het,W9 -3.52656 0.383 62.6 -9.211  <.0001 
##  het,W3 - mut,W9  2.30281 0.486 77.0  4.743  0.0003 
##  mut,W3 - wt,W6  -4.38942 0.582 75.4 -7.540  <.0001 
##  mut,W3 - het,W6 -4.38584 0.534 76.3 -8.208  <.0001 
##  mut,W3 - mut,W6 -0.85516 0.549 75.6 -1.557  0.8246 
##  mut,W3 - wt,W9  -4.94544 0.625 78.0 -7.916  <.0001 
##  mut,W3 - het,W9 -5.10011 0.549 77.5 -9.286  <.0001 
##  mut,W3 - mut,W9  0.72925 0.563 73.6  1.296  0.9294 
##  wt,W6 - het,W6   0.00357 0.427 70.7  0.008  1.0000 
##  wt,W6 - mut,W6   3.53426 0.486 69.8  7.273  <.0001 
##  wt,W6 - wt,W9   -0.55602 0.392 32.7 -1.420  0.8819 
##  wt,W6 - het,W9  -0.71069 0.446 74.7 -1.594  0.8049 
##  wt,W6 - mut,W9   5.11867 0.506 73.8 10.125  <.0001 
##  het,W6 - mut,W6  3.53068 0.427 70.4  8.259  <.0001 
##  het,W6 - wt,W9  -0.55960 0.484 78.0 -1.157  0.9629 
##  het,W6 - het,W9 -0.71427 0.269 31.1 -2.654  0.2058 
##  het,W6 - mut,W9  5.11509 0.450 75.0 11.376  <.0001 
##  mut,W6 - wt,W9  -4.09028 0.536 77.3 -7.628  <.0001 
##  mut,W6 - het,W9 -4.24495 0.446 74.5 -9.519  <.0001 
##  mut,W6 - mut,W9  1.58441 0.346 30.2  4.585  0.0021 
##  wt,W9 - het,W9  -0.15467 0.500 77.8 -0.309  1.0000 
##  wt,W9 - mut,W9   5.67469 0.554 77.9 10.243  <.0001 
##  het,W9 - mut,W9  5.82936 0.467 77.1 12.476  <.0001 
## 
## Degrees-of-freedom method: kenward-roger 
## P value adjustment: tukey method for comparing a family of 9 estimates
```

```
summary(Hippocampuslm)
```

```
## Linear mixed model fit by REML. t-tests use Satterthwaite's method [
## lmerModLmerTest]
## Formula: Hippocampus.Volume ~ genotype * timepoint + (1 | mouse)
##    Data: .
## 
## REML criterion at convergence: 243.8
## 
## Scaled residuals: 
##      Min       1Q   Median       3Q      Max 
## -2.57998 -0.41941  0.06812  0.40032  1.97820 
## 
## Random effects:
##  Groups   Name        Variance Std.Dev.
##  mouse    (Intercept) 0.7239   0.8508  
##  Residual             0.4946   0.7033  
## Number of obs: 87, groups:  mouse, 55
## 
## Fixed effects:
##                         Estimate Std. Error      df t value Pr(>|t|)    
## (Intercept)              28.9207     0.4282 76.0811  67.544  < 2e-16 ***
## genotypehet              -0.6428     0.5284 76.8221  -1.217 0.227483    
## genotypemut              -2.2164     0.6315 76.7776  -3.510 0.000754 ***
## timepointW6               2.1730     0.5138 77.1362   4.229 6.39e-05 ***
## timepointW9               2.7291     0.5510 72.1554   4.953 4.65e-06 ***
## genotypehet:timepointW6   0.6393     0.6275 75.2325   1.019 0.311619    
## genotypemut:timepointW6  -1.3179     0.7442 76.5023  -1.771 0.080565 .  
## genotypehet:timepointW9   0.7975     0.6665 69.7086   1.197 0.235550    
## genotypemut:timepointW9  -3.4583     0.7796 73.0599  -4.436 3.18e-05 ***
## ---
## Signif. codes:  0 '***' 0.001 '**' 0.01 '*' 0.05 '.' 0.1 ' ' 1
## 
## Correlation of Fixed Effects:
##             (Intr) gntyph gntypm tmpnW6 tmpnW9 gntyph:W6 gntypm:W6
## genotypehet -0.810                                                
## genotypemut -0.678  0.549                                         
## timepointW6 -0.752  0.609  0.510                                  
## timepointW9 -0.681  0.551  0.461  0.736                           
## gntypht:tW6  0.616 -0.743 -0.417 -0.819 -0.602                    
## gntypmt:tW6  0.519 -0.421 -0.765 -0.690 -0.508  0.565             
## gntypht:tW9  0.563 -0.679 -0.381 -0.608 -0.827  0.735     0.420   
## gntypmt:tW9  0.481 -0.390 -0.716 -0.520 -0.707  0.426     0.769   
##             gntyph:W9
## genotypehet          
## genotypemut          
## timepointW6          
## timepointW9          
## gntypht:tW6          
## gntypmt:tW6          
## gntypht:tW9          
## gntypmt:tW9  0.584
```

```
Hindbrainlm <- cbdf %>% lmer(Hindbrain.Volume ~ genotype * timepoint + (1 | 
    mouse), data = .)

Hindbrain.Volume.Pairwise <- emmeans(Hindbrainlm, ~genotype * timepoint)
pairs(Hindbrain.Volume.Pairwise)
```

```
##  contrast        estimate    SE   df t.ratio p.value
##  wt,W3 - het,W3    0.9449 0.704 77.5   1.342 0.9153 
##  wt,W3 - mut,W3    3.4124 0.841 77.4   4.059 0.0036 
##  wt,W3 - wt,W6    -7.0210 0.706 77.8  -9.945 <.0001 
##  wt,W3 - het,W6   -6.9498 0.655 76.8 -10.605 <.0001 
##  wt,W3 - mut,W6   -2.6266 0.718 76.5  -3.657 0.0131 
##  wt,W3 - wt,W9    -8.9423 0.782 75.0 -11.442 <.0001 
##  wt,W3 - het,W9   -8.3169 0.685 77.7 -12.144 <.0001 
##  wt,W3 - mut,W9   -2.0091 0.750 77.4  -2.679 0.1728 
##  het,W3 - mut,W3   2.4675 0.748 77.7   3.299 0.0372 
##  het,W3 - wt,W6   -7.9659 0.607 76.9 -13.118 <.0001 
##  het,W3 - het,W6  -7.8947 0.516 74.0 -15.312 <.0001 
##  het,W3 - mut,W6  -3.5715 0.607 76.8  -5.882 <.0001 
##  het,W3 - wt,W9   -9.8872 0.702 77.9 -14.088 <.0001 
##  het,W3 - het,W9  -9.2618 0.547 70.3 -16.929 <.0001 
##  het,W3 - mut,W9  -2.9540 0.644 77.8  -4.584 0.0006 
##  mut,W3 - wt,W6  -10.4334 0.761 77.0 -13.706 <.0001 
##  mut,W3 - het,W6 -10.3622 0.702 77.3 -14.759 <.0001 
##  mut,W3 - mut,W6  -6.0390 0.747 77.3  -8.082 <.0001 
##  mut,W3 - wt,W9  -12.3547 0.839 78.0 -14.732 <.0001 
##  mut,W3 - het,W9 -11.7293 0.730 77.8 -16.074 <.0001 
##  mut,W3 - mut,W9  -5.4214 0.774 76.3  -7.001 <.0001 
##  wt,W6 - het,W6    0.0712 0.550 75.2   0.130 1.0000 
##  wt,W6 - mut,W6    4.3944 0.623 74.9   7.049 <.0001 
##  wt,W6 - wt,W9    -1.9213 0.630 38.7  -3.052 0.0860 
##  wt,W6 - het,W9   -1.2959 0.585 77.1  -2.217 0.4054 
##  wt,W6 - mut,W9    5.0119 0.660 76.7   7.598 <.0001 
##  het,W6 - mut,W6   4.3232 0.550 75.2   7.865 <.0001 
##  het,W6 - wt,W9   -1.9925 0.653 78.0  -3.053 0.0717 
##  het,W6 - het,W9  -1.3671 0.437 35.7  -3.129 0.0742 
##  het,W6 - mut,W9   4.9407 0.590 77.2   8.367 <.0001 
##  mut,W6 - wt,W9   -6.3157 0.716 77.9  -8.823 <.0001 
##  mut,W6 - het,W9  -5.6903 0.585 77.1  -9.735 <.0001 
##  mut,W6 - mut,W9   0.6176 0.564 34.1   1.094 0.9712 
##  wt,W9 - het,W9    0.6254 0.682 77.7   0.917 0.9914 
##  wt,W9 - mut,W9    6.9332 0.748 78.0   9.273 <.0001 
##  het,W9 - mut,W9   6.3078 0.623 77.9  10.124 <.0001 
## 
## Degrees-of-freedom method: kenward-roger 
## P value adjustment: tukey method for comparing a family of 9 estimates
```

```
summary(Hindbrainlm)
```

```
## Linear mixed model fit by REML. t-tests use Satterthwaite's method [
## lmerModLmerTest]
## Formula: Hindbrain.Volume ~ genotype * timepoint + (1 | mouse)
##    Data: .
## 
## REML criterion at convergence: 290
## 
## Scaled residuals: 
##      Min       1Q   Median       3Q      Max 
## -2.46937 -0.41193 -0.05978  0.52779  2.14751 
## 
## Random effects:
##  Groups   Name        Variance Std.Dev.
##  mouse    (Intercept) 0.5862   0.7657  
##  Residual             1.3576   1.1651  
## Number of obs: 87, groups:  mouse, 55
## 
## Fixed effects:
##                         Estimate Std. Error      df t value Pr(>|t|)    
## (Intercept)              39.0882     0.5627 77.3420  69.469  < 2e-16 ***
## genotypehet              -0.9449     0.6981 77.6299  -1.354 0.179804    
## genotypemut              -3.4124     0.8334 77.5559  -4.094 0.000103 ***
## timepointW6               7.0210     0.6983 77.8355  10.055 1.01e-15 ***
## timepointW9               8.9423     0.7703 75.6743  11.609  < 2e-16 ***
## genotypehet:timepointW6   0.8737     0.8635 77.2135   1.012 0.314780    
## genotypemut:timepointW6  -0.9820     1.0160 77.6776  -0.966 0.336806    
## genotypehet:timepointW9   0.3196     0.9398 74.6048   0.340 0.734791    
## genotypemut:timepointW9  -3.5208     1.0850 76.2169  -3.245 0.001745 ** 
## ---
## Signif. codes:  0 '***' 0.001 '**' 0.01 '*' 0.05 '.' 0.1 ' ' 1
## 
## Correlation of Fixed Effects:
##             (Intr) gntyph gntypm tmpnW6 tmpnW9 gntyph:W6 gntypm:W6
## genotypehet -0.806                                                
## genotypemut -0.675  0.544                                         
## timepointW6 -0.778  0.627  0.525                                  
## timepointW9 -0.694  0.559  0.468  0.643                           
## gntypht:tW6  0.629 -0.774 -0.425 -0.809 -0.520                    
## gntypmt:tW6  0.535 -0.431 -0.792 -0.687 -0.442  0.556             
## gntypht:tW9  0.569 -0.700 -0.384 -0.527 -0.820  0.648     0.362   
## gntypmt:tW9  0.492 -0.397 -0.734 -0.457 -0.710  0.369     0.683   
##             gntyph:W9
## genotypehet          
## genotypemut          
## timepointW6          
## timepointW9          
## gntypht:tW6          
## gntypmt:tW6          
## gntypht:tW9          
## gntypmt:tW9  0.582
```

```
WholeBrainlm <- cbdf %>% lmer(WholeBrain.Volume ~ genotype * timepoint + (1 | 
    mouse), data = .)

WholeBrain.Volume.Pairwise <- emmeans(WholeBrainlm, ~genotype * timepoint)
pairs(WholeBrain.Volume.Pairwise)
```

```
##  contrast        estimate   SE   df t.ratio p.value
##  wt,W3 - het,W3     7.048 6.09 77.0   1.157 0.9629 
##  wt,W3 - mut,W3    29.391 7.28 76.9   4.039 0.0038 
##  wt,W3 - wt,W6    -41.218 6.05 77.8  -6.809 <.0001 
##  wt,W3 - het,W6   -41.629 5.69 75.8  -7.315 <.0001 
##  wt,W3 - mut,W6     2.979 6.25 75.0   0.477 0.9999 
##  wt,W3 - wt,W9    -50.072 6.60 73.9  -7.581 <.0001 
##  wt,W3 - het,W9   -49.524 5.91 77.2  -8.376 <.0001 
##  wt,W3 - mut,W9    26.802 6.49 76.7   4.130 0.0028 
##  het,W3 - mut,W3   22.343 6.46 77.5   3.460 0.0235 
##  het,W3 - wt,W6   -48.267 5.27 75.7  -9.158 <.0001 
##  het,W3 - het,W6  -48.677 4.35 72.2 -11.187 <.0001 
##  het,W3 - mut,W6   -4.069 5.27 75.6  -0.772 0.9973 
##  het,W3 - wt,W9   -57.121 5.99 77.9  -9.534 <.0001 
##  het,W3 - het,W9  -56.572 4.57 67.4 -12.368 <.0001 
##  het,W3 - mut,W9   19.754 5.55 77.5   3.557 0.0176 
##  mut,W3 - wt,W6   -70.609 6.61 76.0 -10.689 <.0001 
##  mut,W3 - het,W6  -71.019 6.08 76.7 -11.680 <.0001 
##  mut,W3 - mut,W6  -26.411 6.38 77.1  -4.137 0.0027 
##  mut,W3 - wt,W9   -79.463 7.19 78.0 -11.046 <.0001 
##  mut,W3 - het,W9  -78.914 6.29 77.6 -12.549 <.0001 
##  mut,W3 - mut,W9   -2.588 6.58 75.7  -0.393 1.0000 
##  wt,W6 - het,W6    -0.410 4.80 72.8  -0.085 1.0000 
##  wt,W6 - mut,W6    44.198 5.45 72.2   8.107 <.0001 
##  wt,W6 - wt,W9     -8.854 4.98 35.4  -1.779 0.6947 
##  wt,W6 - het,W9    -8.305 5.06 76.1  -1.640 0.7795 
##  wt,W6 - mut,W9    68.021 5.73 75.4  11.880 <.0001 
##  het,W6 - mut,W6   44.608 4.80 72.6   9.288 <.0001 
##  het,W6 - wt,W9    -8.444 5.58 78.0  -1.512 0.8465 
##  het,W6 - het,W9   -7.895 3.44 33.2  -2.298 0.3715 
##  het,W6 - mut,W9   68.431 5.11 76.2  13.388 <.0001 
##  mut,W6 - wt,W9   -53.052 6.15 77.8  -8.624 <.0001 
##  mut,W6 - het,W9  -52.503 5.06 76.0 -10.369 <.0001 
##  mut,W6 - mut,W9   23.823 4.42 31.9   5.386 0.0002 
##  wt,W9 - het,W9     0.549 5.81 77.7   0.094 1.0000 
##  wt,W9 - mut,W9    76.875 6.40 78.0  12.021 <.0001 
##  het,W9 - mut,W9   76.326 5.36 77.7  14.248 <.0001 
## 
## Degrees-of-freedom method: kenward-roger 
## P value adjustment: tukey method for comparing a family of 9 estimates
```

```
summary(WholeBrainlm)
```

```
## Linear mixed model fit by REML. t-tests use Satterthwaite's method [
## lmerModLmerTest]
## Formula: WholeBrain.Volume ~ genotype * timepoint + (1 | mouse)
##    Data: .
## 
## REML criterion at convergence: 625.2
## 
## Scaled residuals: 
##      Min       1Q   Median       3Q      Max 
## -2.69643 -0.40774  0.01484  0.42720  2.05626 
## 
## Random effects:
##  Groups   Name        Variance Std.Dev.
##  mouse    (Intercept) 68.22    8.260   
##  Residual             82.11    9.061   
## Number of obs: 87, groups:  mouse, 55
## 
## Fixed effects:
##                         Estimate Std. Error      df t value Pr(>|t|)    
## (Intercept)              366.815      4.872  76.612  75.289  < 2e-16 ***
## genotypehet               -7.048      6.031  77.174  -1.169 0.246099    
## genotypemut              -29.391      7.204  77.065  -4.080 0.000109 ***
## timepointW6               41.218      5.964  77.811   6.911 1.17e-09 ***
## timepointW9               50.072      6.496  74.617   7.708 4.41e-11 ***
## genotypehet:timepointW6    7.458      7.336  76.896   1.017 0.312495    
## genotypemut:timepointW6  -14.807      8.662  77.560  -1.709 0.091373 .  
## genotypehet:timepointW9    6.500      7.897  73.042   0.823 0.413152    
## genotypemut:timepointW9  -47.484      9.170  75.388  -5.178 1.80e-06 ***
## ---
## Signif. codes:  0 '***' 0.001 '**' 0.01 '*' 0.05 '.' 0.1 ' ' 1
## 
## Correlation of Fixed Effects:
##             (Intr) gntyph gntypm tmpnW6 tmpnW9 gntyph:W6 gntypm:W6
## genotypehet -0.808                                                
## genotypemut -0.676  0.546                                         
## timepointW6 -0.767  0.620  0.519                                  
## timepointW9 -0.688  0.556  0.465  0.690                           
## gntypht:tW6  0.624 -0.761 -0.422 -0.813 -0.561                    
## gntypmt:tW6  0.528 -0.427 -0.781 -0.689 -0.475  0.560             
## gntypht:tW9  0.566 -0.691 -0.383 -0.567 -0.823  0.692     0.391   
## gntypmt:tW9  0.487 -0.394 -0.726 -0.489 -0.708  0.397     0.726   
##             gntyph:W9
## genotypehet          
## genotypemut          
## timepointW6          
## timepointW9          
## gntypht:tW6          
## gntypmt:tW6          
## gntypht:tW9          
## gntypmt:tW9  0.583
```

```
Midbrainlm <- cbdf %>% lmer(Midbrain.Volume ~ genotype * timepoint + (1 | mouse), 
    data = .)

Midbrain.Volume.Pairwise <- emmeans(Midbrainlm, ~genotype * timepoint)
pairs(Midbrain.Volume.Pairwise)
```

```
##  contrast        estimate    SE   df t.ratio p.value
##  wt,W3 - het,W3     0.609 0.499 77.2  1.219  0.9498 
##  wt,W3 - mut,W3     2.269 0.596 77.0  3.806  0.0082 
##  wt,W3 - wt,W6     -1.716 0.498 77.8 -3.447  0.0244 
##  wt,W3 - het,W6    -1.511 0.466 76.1 -3.244  0.0435 
##  wt,W3 - mut,W6     1.190 0.511 75.5  2.329  0.3388 
##  wt,W3 - wt,W9     -2.082 0.546 74.4 -3.812  0.0082 
##  wt,W3 - het,W9    -1.862 0.485 77.4 -3.841  0.0073 
##  wt,W3 - mut,W9     2.837 0.532 76.9  5.335  <.0001 
##  het,W3 - mut,W3    1.661 0.530 77.5  3.136  0.0579 
##  het,W3 - wt,W6    -2.325 0.431 76.1 -5.389  <.0001 
##  het,W3 - het,W6   -2.119 0.360 73.0 -5.886  <.0001 
##  het,W3 - mut,W6    0.581 0.431 76.1  1.347  0.9134 
##  het,W3 - wt,W9    -2.690 0.493 77.8 -5.452  <.0001 
##  het,W3 - het,W9   -2.471 0.380 68.6 -6.506  <.0001 
##  het,W3 - mut,W9    2.228 0.456 77.6  4.889  0.0002 
##  mut,W3 - wt,W6    -3.985 0.541 76.3 -7.371  <.0001 
##  mut,W3 - het,W6   -3.780 0.498 76.9 -7.589  <.0001 
##  mut,W3 - mut,W6   -1.080 0.526 77.3 -2.053  0.5121 
##  mut,W3 - wt,W9    -4.351 0.591 78.0 -7.357  <.0001 
##  mut,W3 - het,W9   -4.132 0.516 77.7 -8.006  <.0001 
##  mut,W3 - mut,W9    0.567 0.543 76.0  1.045  0.9799 
##  wt,W6 - het,W6     0.205 0.392 73.6  0.524  0.9998 
##  wt,W6 - mut,W6     2.906 0.445 73.1  6.531  <.0001 
##  wt,W6 - wt,W9     -0.366 0.422 36.5 -0.868  0.9934 
##  wt,W6 - het,W9    -0.147 0.415 76.5 -0.353  1.0000 
##  wt,W6 - mut,W9     4.553 0.469 75.9  9.716  <.0001 
##  het,W6 - mut,W6    2.700 0.392 73.5  6.888  <.0001 
##  het,W6 - wt,W9    -0.571 0.460 78.0 -1.243  0.9441 
##  het,W6 - het,W9   -0.352 0.292 34.0 -1.207  0.9495 
##  het,W6 - mut,W9    4.347 0.419 76.6 10.382  <.0001 
##  mut,W6 - wt,W9    -3.272 0.505 77.8 -6.474  <.0001 
##  mut,W6 - het,W9   -3.052 0.415 76.4 -7.360  <.0001 
##  mut,W6 - mut,W9    1.647 0.376 32.6  4.382  0.0032 
##  wt,W9 - het,W9     0.219 0.479 77.7  0.458  0.9999 
##  wt,W9 - mut,W9     4.919 0.526 78.0  9.345  <.0001 
##  het,W9 - mut,W9    4.699 0.440 77.8 10.680  <.0001 
## 
## Degrees-of-freedom method: kenward-roger 
## P value adjustment: tukey method for comparing a family of 9 estimates
```

```
summary(Midbrainlm)
```

```
## Linear mixed model fit by REML. t-tests use Satterthwaite's method [
## lmerModLmerTest]
## Formula: Midbrain.Volume ~ genotype * timepoint + (1 | mouse)
##    Data: .
## 
## REML criterion at convergence: 235.5
## 
## Scaled residuals: 
##      Min       1Q   Median       3Q      Max 
## -2.57662 -0.38989  0.04054  0.48380  1.76549 
## 
## Random effects:
##  Groups   Name        Variance Std.Dev.
##  mouse    (Intercept) 0.4003   0.6327  
##  Residual             0.5960   0.7720  
## Number of obs: 87, groups:  mouse, 55
## 
## Fixed effects:
##                         Estimate Std. Error      df t value Pr(>|t|)    
## (Intercept)              29.2695     0.3991 76.8745  73.335  < 2e-16 ***
## genotypehet              -0.6087     0.4945 77.3401  -1.231 0.222072    
## genotypemut              -2.2693     0.5906 77.2369  -3.843 0.000248 ***
## timepointW6               1.7160     0.4912 77.8555   3.494 0.000789 ***
## timepointW9               2.0818     0.5376 75.1140   3.872 0.000228 ***
## genotypehet:timepointW6   0.4033     0.6054 77.1035   0.666 0.507264    
## genotypemut:timepointW6  -0.6364     0.7139 77.6590  -0.891 0.375422    
## genotypehet:timepointW9   0.3894     0.6544 73.7609   0.595 0.553686    
## genotypemut:timepointW9  -2.6493     0.7582 75.8034  -3.494 0.000798 ***
## ---
## Signif. codes:  0 '***' 0.001 '**' 0.01 '*' 0.05 '.' 0.1 ' ' 1
## 
## Correlation of Fixed Effects:
##             (Intr) gntyph gntypm tmpnW6 tmpnW9 gntyph:W6 gntypm:W6
## genotypehet -0.807                                                
## genotypemut -0.676  0.546                                         
## timepointW6 -0.772  0.623  0.522                                  
## timepointW9 -0.690  0.557  0.466  0.673                           
## gntypht:tW6  0.626 -0.766 -0.423 -0.811 -0.546                    
## gntypmt:tW6  0.531 -0.429 -0.785 -0.688 -0.463  0.558             
## gntypht:tW9  0.567 -0.694 -0.383 -0.553 -0.822  0.677     0.381   
## gntypmt:tW9  0.489 -0.395 -0.729 -0.477 -0.709  0.387     0.711   
##             gntyph:W9
## genotypehet          
## genotypemut          
## timepointW6          
## timepointW9          
## gntypht:tW6          
## gntypmt:tW6          
## gntypht:tW9          
## gntypmt:tW9  0.582
```

```
Thalamuslm <- cbdf %>% lmer(Thalamus.Volume ~ genotype * timepoint + (1 | mouse), 
    data = .)

Thalamus.Volume.Pairwise <- emmeans(Thalamuslm, ~genotype * timepoint)
pairs(Thalamus.Volume.Pairwise)
```

```
##  contrast        estimate    SE   df t.ratio p.value
##  wt,W3 - het,W3    0.2343 0.229 76.9   1.025 0.9822 
##  wt,W3 - mut,W3    0.8183 0.273 76.8   2.995 0.0831 
##  wt,W3 - wt,W6    -1.2017 0.227 77.7  -5.305 <.0001 
##  wt,W3 - het,W6   -1.1460 0.214 75.5  -5.358 <.0001 
##  wt,W3 - mut,W6    0.5202 0.235 74.6   2.214 0.4078 
##  wt,W3 - wt,W9    -1.5539 0.246 73.5  -6.316 <.0001 
##  wt,W3 - het,W9   -1.5147 0.222 77.1  -6.829 <.0001 
##  wt,W3 - mut,W9    1.5950 0.244 76.5   6.548 <.0001 
##  het,W3 - mut,W3   0.5840 0.242 77.4   2.411 0.2928 
##  het,W3 - wt,W6   -1.4360 0.198 75.4  -7.250 <.0001 
##  het,W3 - het,W6  -1.3803 0.162 71.4  -8.522 <.0001 
##  het,W3 - mut,W6   0.2859 0.198 75.3   1.443 0.8771 
##  het,W3 - wt,W9   -1.7882 0.224 77.9  -7.985 <.0001 
##  het,W3 - het,W9  -1.7490 0.170 66.3 -10.302 <.0001 
##  het,W3 - mut,W9   1.3607 0.208 77.3   6.535 <.0001 
##  mut,W3 - wt,W6   -2.0201 0.248 75.8  -8.139 <.0001 
##  mut,W3 - het,W6  -1.9643 0.228 76.5  -8.603 <.0001 
##  mut,W3 - mut,W6  -0.2981 0.239 76.9  -1.250 0.9424 
##  mut,W3 - wt,W9   -2.3722 0.269 78.0  -8.809 <.0001 
##  mut,W3 - het,W9  -2.3330 0.236 77.6  -9.896 <.0001 
##  mut,W3 - mut,W9   0.7767 0.246 75.3   3.163 0.0542 
##  wt,W6 - het,W6    0.0557 0.181 72.2   0.308 1.0000 
##  wt,W6 - mut,W6    1.7219 0.205 71.5   8.384 <.0001 
##  wt,W6 - wt,W9    -0.3522 0.182 34.6  -1.937 0.5940 
##  wt,W6 - het,W9   -0.3130 0.190 75.7  -1.646 0.7763 
##  wt,W6 - mut,W9    2.7967 0.215 74.9  12.997 <.0001 
##  het,W6 - mut,W6   1.6662 0.181 72.0   9.212 <.0001 
##  het,W6 - wt,W9   -0.4079 0.209 78.0  -1.953 0.5799 
##  het,W6 - het,W9  -0.3687 0.125 32.6  -2.942 0.1153 
##  het,W6 - mut,W9   2.7410 0.192 75.9  14.282 <.0001 
##  mut,W6 - wt,W9   -2.0741 0.230 77.7  -9.001 <.0001 
##  mut,W6 - het,W9  -2.0349 0.190 75.6 -10.700 <.0001 
##  mut,W6 - mut,W9   1.0748 0.161 31.4   6.666 <.0001 
##  wt,W9 - het,W9    0.0392 0.217 77.7   0.181 1.0000 
##  wt,W9 - mut,W9    3.1489 0.239 78.0  13.163 <.0001 
##  het,W9 - mut,W9   3.1097 0.201 77.6  15.493 <.0001 
## 
## Degrees-of-freedom method: kenward-roger 
## P value adjustment: tukey method for comparing a family of 9 estimates
```

```
summary(Thalamuslm)
```

```
## Linear mixed model fit by REML. t-tests use Satterthwaite's method [
## lmerModLmerTest]
## Formula: Thalamus.Volume ~ genotype * timepoint + (1 | mouse)
##    Data: .
## 
## REML criterion at convergence: 112.7
## 
## Scaled residuals: 
##      Min       1Q   Median       3Q      Max 
## -2.15730 -0.45947  0.00447  0.32022  2.17791 
## 
## Random effects:
##  Groups   Name        Variance Std.Dev.
##  mouse    (Intercept) 0.1056   0.3249  
##  Residual             0.1087   0.3297  
## Number of obs: 87, groups:  mouse, 55
## 
## Fixed effects:
##                         Estimate Std. Error      df t value Pr(>|t|)    
## (Intercept)              13.6273     0.1829 76.2098  74.493  < 2e-16 ***
## genotypehet              -0.2343     0.2263 76.9243  -1.036  0.30359    
## genotypemut              -0.8183     0.2703 76.8045  -3.027  0.00336 ** 
## timepointW6               1.2017     0.2229 77.6975   5.391 7.31e-07 ***
## timepointW9               1.5539     0.2419 73.6134   6.425 1.16e-08 ***
## genotypehet:timepointW6   0.1786     0.2737 76.4498   0.652  0.51607    
## genotypemut:timepointW6  -0.9036     0.3236 77.3390  -2.793  0.00658 ** 
## genotypehet:timepointW9   0.1951     0.2937 71.6205   0.664  0.50852    
## genotypemut:timepointW9  -2.3306     0.3416 74.5445  -6.822 2.04e-09 ***
## ---
## Signif. codes:  0 '***' 0.001 '**' 0.01 '*' 0.05 '.' 0.1 ' ' 1
## 
## Correlation of Fixed Effects:
##             (Intr) gntyph gntypm tmpnW6 tmpnW9 gntyph:W6 gntypm:W6
## genotypehet -0.809                                                
## genotypemut -0.677  0.547                                         
## timepointW6 -0.764  0.618  0.517                                  
## timepointW9 -0.686  0.555  0.464  0.702                           
## gntypht:tW6  0.622 -0.757 -0.421 -0.814 -0.572                    
## gntypmt:tW6  0.526 -0.426 -0.778 -0.689 -0.484  0.561             
## gntypht:tW9  0.565 -0.688 -0.383 -0.578 -0.824  0.704     0.398   
## gntypmt:tW9  0.486 -0.393 -0.724 -0.497 -0.708  0.405     0.738   
##             gntyph:W9
## genotypehet          
## genotypemut          
## timepointW6          
## timepointW9          
## gntypht:tW6          
## gntypmt:tW6          
## gntypht:tW9          
## gntypmt:tW9  0.583
```

```
Hypothalamuslm <- cbdf %>% lmer(Hypothalamus.Volume ~ genotype * timepoint + 
    (1 | mouse), data = .)

Hypothalamus.Volume.Pairwise <- emmeans(Hypothalamuslm, ~genotype * timepoint)
pairs(Hypothalamus.Volume.Pairwise)
```

```
##  contrast        estimate     SE   df t.ratio p.value
##  wt,W3 - het,W3    0.2632 0.1605 77.5  1.640  0.7799 
##  wt,W3 - mut,W3    0.3286 0.1916 77.4  1.715  0.7356 
##  wt,W3 - wt,W6    -0.7590 0.1608 77.8 -4.721  0.0003 
##  wt,W3 - het,W6   -0.5239 0.1494 76.7 -3.507  0.0206 
##  wt,W3 - mut,W6    0.0982 0.1638 76.3  0.599  0.9996 
##  wt,W3 - wt,W9    -1.0125 0.1778 74.9 -5.696  <.0001 
##  wt,W3 - het,W9   -0.9074 0.1560 77.6 -5.815  <.0001 
##  wt,W3 - mut,W9    0.5045 0.1709 77.3  2.952  0.0924 
##  het,W3 - mut,W3   0.0654 0.1704 77.7  0.384  1.0000 
##  het,W3 - wt,W6   -1.0222 0.1384 76.8 -7.384  <.0001 
##  het,W3 - het,W6  -0.7871 0.1172 73.9 -6.713  <.0001 
##  het,W3 - mut,W6  -0.1650 0.1384 76.7 -1.192  0.9558 
##  het,W3 - wt,W9   -1.2757 0.1598 77.9 -7.985  <.0001 
##  het,W3 - het,W9  -1.1706 0.1243 70.0 -9.417  <.0001 
##  het,W3 - mut,W9   0.2414 0.1468 77.8  1.644  0.7775 
##  mut,W3 - wt,W6   -1.0876 0.1735 76.9 -6.267  <.0001 
##  mut,W3 - het,W6  -0.8525 0.1600 77.3 -5.327  <.0001 
##  mut,W3 - mut,W6  -0.2304 0.1701 77.3 -1.355  0.9109 
##  mut,W3 - wt,W9   -1.3411 0.1910 78.0 -7.022  <.0001 
##  mut,W3 - het,W9  -1.2360 0.1663 77.8 -7.434  <.0001 
##  mut,W3 - mut,W9   0.1759 0.1762 76.3  0.998  0.9850 
##  wt,W6 - het,W6    0.2351 0.1254 75.0  1.875  0.6328 
##  wt,W6 - mut,W6    0.8571 0.1422 74.6  6.027  <.0001 
##  wt,W6 - wt,W9    -0.2535 0.1423 38.4 -1.782  0.6931 
##  wt,W6 - het,W9   -0.1484 0.1332 77.0 -1.114  0.9704 
##  wt,W6 - mut,W9    1.2635 0.1504 76.6  8.402  <.0001 
##  het,W6 - mut,W6   0.6221 0.1254 74.9  4.961  0.0001 
##  het,W6 - wt,W9   -0.4886 0.1486 78.0 -3.288  0.0384 
##  het,W6 - het,W9  -0.3834 0.0987 35.5 -3.886  0.0113 
##  het,W6 - mut,W9   1.0285 0.1346 77.1  7.642  <.0001 
##  mut,W6 - wt,W9   -1.1106 0.1630 77.9 -6.812  <.0001 
##  mut,W6 - het,W9  -1.0055 0.1332 77.0 -7.547  <.0001 
##  mut,W6 - mut,W9   0.4064 0.1274 33.9  3.190  0.0660 
##  wt,W9 - het,W9    0.1051 0.1553 77.7  0.677  0.9989 
##  wt,W9 - mut,W9    1.5170 0.1702 78.0  8.912  <.0001 
##  het,W9 - mut,W9   1.4119 0.1419 77.9  9.948  <.0001 
## 
## Degrees-of-freedom method: kenward-roger 
## P value adjustment: tukey method for comparing a family of 9 estimates
```

```
summary(Hypothalamuslm)
```

```
## Linear mixed model fit by REML. t-tests use Satterthwaite's method [
## lmerModLmerTest]
## Formula: Hypothalamus.Volume ~ genotype * timepoint + (1 | mouse)
##    Data: .
## 
## REML criterion at convergence: 59.2
## 
## Scaled residuals: 
##      Min       1Q   Median       3Q      Max 
## -2.93671 -0.47613 -0.09864  0.51046  2.27365 
## 
## Random effects:
##  Groups   Name        Variance Std.Dev.
##  mouse    (Intercept) 0.03217  0.1794  
##  Residual             0.06907  0.2628  
## Number of obs: 87, groups:  mouse, 55
## 
## Fixed effects:
##                         Estimate Std. Error       df t value Pr(>|t|)    
## (Intercept)              9.89352    0.12825 77.28765  77.145  < 2e-16 ***
## genotypehet             -0.26319    0.15908 77.59602  -1.654   0.1021    
## genotypemut             -0.32860    0.18993 77.51815  -1.730   0.0876 .  
## timepointW6              0.75898    0.15896 77.84990   4.775 8.29e-06 ***
## timepointW9              1.01248    0.17515 75.66095   5.781 1.57e-07 ***
## genotypehet:timepointW6  0.02812    0.19648 77.23192   0.143   0.8866    
## genotypemut:timepointW6 -0.52854    0.23126 77.69291  -2.285   0.0250 *  
## genotypehet:timepointW9  0.15807    0.21362 74.57832   0.740   0.4617    
## genotypemut:timepointW9 -1.18843    0.24675 76.21451  -4.816 7.27e-06 ***
## ---
## Signif. codes:  0 '***' 0.001 '**' 0.01 '*' 0.05 '.' 0.1 ' ' 1
## 
## Correlation of Fixed Effects:
##             (Intr) gntyph gntypm tmpnW6 tmpnW9 gntyph:W6 gntypm:W6
## genotypehet -0.806                                                
## genotypemut -0.675  0.544                                         
## timepointW6 -0.777  0.627  0.525                                  
## timepointW9 -0.693  0.559  0.468  0.648                           
## gntypht:tW6  0.629 -0.773 -0.425 -0.809 -0.524                    
## gntypmt:tW6  0.534 -0.431 -0.791 -0.687 -0.445  0.556             
## gntypht:tW9  0.568 -0.699 -0.384 -0.531 -0.820  0.652     0.365   
## gntypmt:tW9  0.492 -0.397 -0.733 -0.460 -0.710  0.372     0.687   
##             gntyph:W9
## genotypehet          
## genotypemut          
## timepointW6          
## timepointW9          
## gntypht:tW6          
## gntypmt:tW6          
## gntypht:tW9          
## gntypmt:tW9  0.582
```

# Linear modeling of signal trends over time

```
Cerebellumlm.Signal <- cbdf %>% lmer(CB.Mean/WholeBrain.Mean ~ genotype * timepoint + 
    (1 | mouse), data = .)


Cerebellum.SignalPairwise <- emmeans(Cerebellumlm.Signal, ~genotype * timepoint)
pairs(Cerebellum.SignalPairwise)
```

```
##  contrast        estimate     SE   df t.ratio p.value
##  wt,W3 - het,W3   0.01863 0.0219 78.0  0.849  0.9948 
##  wt,W3 - mut,W3  -0.05117 0.0262 78.0 -1.956  0.5776 
##  wt,W3 - wt,W6    0.03517 0.0222 76.7  1.583  0.8109 
##  wt,W3 - het,W6   0.07109 0.0203 78.0  3.499  0.0209 
##  wt,W3 - mut,W6   0.04488 0.0222 78.0  2.018  0.5356 
##  wt,W3 - wt,W9    0.03829 0.0249 75.5  1.535  0.8352 
##  wt,W3 - het,W9   0.06997 0.0213 78.0  3.280  0.0392 
##  wt,W3 - mut,W9  -0.09992 0.0233 78.0 -4.286  0.0016 
##  het,W3 - mut,W3 -0.06981 0.0233 78.0 -2.991  0.0837 
##  het,W3 - wt,W6   0.01654 0.0188 78.0  0.878  0.9935 
##  het,W3 - het,W6  0.05246 0.0165 74.2  3.178  0.0523 
##  het,W3 - mut,W6  0.02625 0.0188 78.0  1.394  0.8970 
##  het,W3 - wt,W9   0.01966 0.0220 78.0  0.892  0.9928 
##  het,W3 - het,W9  0.05134 0.0177 72.4  2.901  0.1055 
##  het,W3 - mut,W9 -0.11855 0.0201 78.0 -5.899  <.0001 
##  mut,W3 - wt,W6   0.08635 0.0236 78.0  3.656  0.0130 
##  mut,W3 - het,W6  0.12227 0.0218 78.0  5.602  <.0001 
##  mut,W3 - mut,W6  0.09605 0.0236 76.4  4.070  0.0035 
##  mut,W3 - wt,W9   0.08946 0.0263 78.0  3.408  0.0273 
##  mut,W3 - het,W9  0.12114 0.0228 78.0  5.320  <.0001 
##  mut,W3 - mut,W9 -0.04874 0.0246 75.9 -1.982  0.5603 
##  wt,W6 - het,W6   0.03592 0.0169 78.0  2.123  0.4660 
##  wt,W6 - mut,W6   0.00970 0.0192 78.0  0.506  0.9999 
##  wt,W6 - wt,W9    0.00311 0.0221 45.8  0.141  1.0000 
##  wt,W6 - het,W9   0.03480 0.0181 78.0  1.919  0.6028 
##  wt,W6 - mut,W9  -0.13509 0.0204 78.0 -6.615  <.0001 
##  het,W6 - mut,W6 -0.02622 0.0169 78.0 -1.549  0.8284 
##  het,W6 - wt,W9  -0.03281 0.0204 78.0 -1.605  0.7991 
##  het,W6 - het,W9 -0.00113 0.0155 41.6 -0.073  1.0000 
##  het,W6 - mut,W9 -0.17101 0.0183 78.0 -9.336  <.0001 
##  mut,W6 - wt,W9  -0.00659 0.0223 78.0 -0.295  1.0000 
##  mut,W6 - het,W9  0.02509 0.0181 78.0  1.384  0.9005 
##  mut,W6 - mut,W9 -0.14479 0.0202 39.4 -7.164  <.0001 
##  wt,W9 - het,W9   0.03168 0.0214 78.0  1.477  0.8626 
##  wt,W9 - mut,W9  -0.13820 0.0234 78.0 -5.902  <.0001 
##  het,W9 - mut,W9 -0.16988 0.0194 78.0 -8.740  <.0001 
## 
## Degrees-of-freedom method: kenward-roger 
## P value adjustment: tukey method for comparing a family of 9 estimates
```

```
summary(Cerebellumlm.Signal)
```

```
## Linear mixed model fit by REML. t-tests use Satterthwaite's method [
## lmerModLmerTest]
## Formula: CB.Mean/WholeBrain.Mean ~ genotype * timepoint + (1 | mouse)
##    Data: .
## 
## REML criterion at convergence: -250.6
## 
## Scaled residuals: 
##     Min      1Q  Median      3Q     Max 
## -2.6896 -0.6589  0.1038  0.4968  2.7635 
## 
## Random effects:
##  Groups   Name        Variance  Std.Dev.
##  mouse    (Intercept) 3.112e-05 0.005579
##  Residual             1.799e-03 0.042417
## Number of obs: 87, groups:  mouse, 55
## 
## Fixed effects:
##                         Estimate Std. Error       df t value Pr(>|t|)    
## (Intercept)              1.02467    0.01746 77.99841  58.670   <2e-16 ***
## genotypehet             -0.01863    0.02171 77.99944  -0.858   0.3935    
## genotypemut              0.05117    0.02590 77.99917   1.976   0.0517 .  
## timepointW6             -0.03517    0.02207 76.79582  -1.594   0.1151    
## timepointW9             -0.03829    0.02466 75.69898  -1.552   0.1248    
## genotypehet:timepointW6 -0.01729    0.02746 76.11519  -0.630   0.5308    
## genotypemut:timepointW6 -0.06088    0.03217 76.68682  -1.893   0.0622 .  
## genotypehet:timepointW9 -0.01305    0.03023 74.87531  -0.432   0.6673    
## genotypemut:timepointW9  0.08703    0.03466 75.88820   2.511   0.0142 *  
## ---
## Signif. codes:  0 '***' 0.001 '**' 0.01 '*' 0.05 '.' 0.1 ' ' 1
## 
## Correlation of Fixed Effects:
##             (Intr) gntyph gntypm tmpnW6 tmpnW9 gntyph:W6 gntypm:W6
## genotypehet -0.804                                                
## genotypemut -0.674  0.542                                         
## timepointW6 -0.790  0.636  0.533                                  
## timepointW9 -0.706  0.568  0.476  0.564                           
## gntypht:tW6  0.635 -0.789 -0.428 -0.804 -0.453                    
## gntypmt:tW6  0.542 -0.436 -0.804 -0.686 -0.387  0.551             
## gntypht:tW9  0.576 -0.716 -0.388 -0.460 -0.816  0.571     0.315   
## gntypmt:tW9  0.502 -0.404 -0.745 -0.401 -0.712  0.322     0.605   
##             gntyph:W9
## genotypehet          
## genotypemut          
## timepointW6          
## timepointW9          
## gntypht:tW6          
## gntypmt:tW6          
## gntypht:tW9          
## gntypmt:tW9  0.580
```

```
OlfactoryBulblm.Signal <- cbdf %>% lmer(OlfactoryBulb.Mean/WholeBrain.Mean ~ 
    genotype * timepoint + (1 | mouse), data = .)
```

```
## boundary (singular) fit: see ?isSingular
```

```
OlfactoryBulb.SignalPairwise <- emmeans(OlfactoryBulblm.Signal, ~genotype * 
    timepoint)
pairs(OlfactoryBulb.SignalPairwise)
```

```
##  contrast        estimate     SE   df t.ratio p.value
##  wt,W3 - het,W3  -0.00987 0.0267 78.0 -0.369  1.0000 
##  wt,W3 - mut,W3   0.09756 0.0319 78.0  3.058  0.0707 
##  wt,W3 - wt,W6   -0.15400 0.0271 76.5 -5.681  <.0001 
##  wt,W3 - het,W6  -0.15909 0.0248 78.0 -6.422  <.0001 
##  wt,W3 - mut,W6  -0.10001 0.0271 78.0 -3.689  0.0117 
##  wt,W3 - wt,W9   -0.14460 0.0304 75.5 -4.751  0.0003 
##  wt,W3 - het,W9  -0.15531 0.0260 78.0 -5.971  <.0001 
##  wt,W3 - mut,W9   0.07862 0.0284 78.0  2.766  0.1426 
##  het,W3 - mut,W3  0.10743 0.0285 78.0  3.774  0.0090 
##  het,W3 - wt,W6  -0.14414 0.0230 78.0 -6.276  <.0001 
##  het,W3 - het,W6 -0.14922 0.0202 74.1 -7.404  <.0001 
##  het,W3 - mut,W6 -0.09014 0.0230 78.0 -3.925  0.0055 
##  het,W3 - wt,W9  -0.13473 0.0269 78.0 -5.012  0.0001 
##  het,W3 - het,W9 -0.14544 0.0216 72.5 -6.730  <.0001 
##  het,W3 - mut,W9  0.08849 0.0245 78.0  3.611  0.0149 
##  mut,W3 - wt,W6  -0.25156 0.0288 78.0 -8.735  <.0001 
##  mut,W3 - het,W6 -0.25665 0.0266 78.0 -9.644  <.0001 
##  mut,W3 - mut,W6 -0.19757 0.0288 76.3 -6.860  <.0001 
##  mut,W3 - wt,W9  -0.24216 0.0320 78.0 -7.565  <.0001 
##  mut,W3 - het,W9 -0.25287 0.0278 78.0 -9.107  <.0001 
##  mut,W3 - mut,W9 -0.01894 0.0300 75.8 -0.631  0.9994 
##  wt,W6 - het,W6  -0.00509 0.0206 78.0 -0.247  1.0000 
##  wt,W6 - mut,W6   0.05399 0.0234 78.0  2.309  0.3496 
##  wt,W6 - wt,W9    0.00940 0.0272 46.3  0.346  1.0000 
##  wt,W6 - het,W9  -0.00131 0.0221 78.0 -0.059  1.0000 
##  wt,W6 - mut,W9   0.23262 0.0249 78.0  9.344  <.0001 
##  het,W6 - mut,W6  0.05908 0.0206 78.0  2.864  0.1139 
##  het,W6 - wt,W9   0.01449 0.0249 78.0  0.581  0.9997 
##  het,W6 - het,W9  0.00378 0.0191 42.0  0.198  1.0000 
##  het,W6 - mut,W9  0.23771 0.0223 78.0 10.646  <.0001 
##  mut,W6 - wt,W9  -0.04459 0.0272 78.0 -1.637  0.7814 
##  mut,W6 - het,W9 -0.05530 0.0221 78.0 -2.502  0.2470 
##  mut,W6 - mut,W9  0.17863 0.0248 39.8  7.196  <.0001 
##  wt,W9 - het,W9  -0.01071 0.0261 78.0 -0.410  1.0000 
##  wt,W9 - mut,W9   0.22322 0.0285 78.0  7.820  <.0001 
##  het,W9 - mut,W9  0.23393 0.0237 78.0  9.873  <.0001 
## 
## Degrees-of-freedom method: kenward-roger 
## P value adjustment: tukey method for comparing a family of 9 estimates
```

```
summary(OlfactoryBulblm.Signal)
```

```
## Linear mixed model fit by REML. t-tests use Satterthwaite's method [
## lmerModLmerTest]
## Formula: OlfactoryBulb.Mean/WholeBrain.Mean ~ genotype * timepoint + (1 |  
##     mouse)
##    Data: .
## 
## REML criterion at convergence: -219.7
## 
## Scaled residuals: 
##     Min      1Q  Median      3Q     Max 
## -3.6514 -0.5282  0.0369  0.6022  2.1109 
## 
## Random effects:
##  Groups   Name        Variance Std.Dev.
##  mouse    (Intercept) 0.00000  0.00000 
##  Residual             0.00272  0.05215 
## Number of obs: 87, groups:  mouse, 55
## 
## Fixed effects:
##                          Estimate Std. Error        df t value Pr(>|t|)
## (Intercept)              0.779581   0.021291 78.000000  36.616  < 2e-16
## genotypehet              0.009868   0.026468 78.000000   0.373  0.71029
## genotypemut             -0.097558   0.031580 78.000000  -3.089  0.00278
## timepointW6              0.154003   0.026931 78.000000   5.718 1.89e-07
## timepointW9              0.144603   0.030110 78.000000   4.803 7.43e-06
## genotypehet:timepointW6 -0.004781   0.033521 78.000000  -0.143  0.88695
## genotypemut:timepointW6  0.043564   0.039259 78.000000   1.110  0.27055
## genotypehet:timepointW9  0.000842   0.036920 78.000000   0.023  0.98186
## genotypemut:timepointW9 -0.125666   0.042315 78.000000  -2.970  0.00396
##                            
## (Intercept)             ***
## genotypehet                
## genotypemut             ** 
## timepointW6             ***
## timepointW9             ***
## genotypehet:timepointW6    
## genotypemut:timepointW6    
## genotypehet:timepointW9    
## genotypemut:timepointW9 ** 
## ---
## Signif. codes:  0 '***' 0.001 '**' 0.01 '*' 0.05 '.' 0.1 ' ' 1
## 
## Correlation of Fixed Effects:
##             (Intr) gntyph gntypm tmpnW6 tmpnW9 gntyph:W6 gntypm:W6
## genotypehet -0.804                                                
## genotypemut -0.674  0.542                                         
## timepointW6 -0.791  0.636  0.533                                  
## timepointW9 -0.707  0.569  0.477  0.559                           
## gntypht:tW6  0.635 -0.790 -0.428 -0.803 -0.449                    
## gntypmt:tW6  0.542 -0.436 -0.804 -0.686 -0.383  0.551             
## gntypht:tW9  0.577 -0.717 -0.389 -0.456 -0.816  0.566     0.313   
## gntypmt:tW9  0.503 -0.405 -0.746 -0.398 -0.712  0.320     0.600   
##             gntyph:W9
## genotypehet          
## genotypemut          
## timepointW6          
## timepointW9          
## gntypht:tW6          
## gntypmt:tW6          
## gntypht:tW9          
## gntypmt:tW9  0.580   
## convergence code: 0
## boundary (singular) fit: see ?isSingular
```

```
Cortexlm.Signal <- cbdf %>% lmer(Cortex.Mean/WholeBrain.Mean ~ genotype * timepoint + 
    (1 | mouse), data = .)

Cortex.SignalPairwise <- emmeans(Cortexlm.Signal, ~genotype * timepoint)
pairs(Cortex.SignalPairwise)
```

```
##  contrast        estimate      SE   df t.ratio p.value
##  wt,W3 - het,W3  -0.00283 0.01009 77.9 -0.281  1.0000 
##  wt,W3 - mut,W3   0.02655 0.01203 77.8  2.206  0.4122 
##  wt,W3 - wt,W6   -0.01176 0.01016 77.5 -1.157  0.9630 
##  wt,W3 - het,W6  -0.00555 0.00936 77.6 -0.593  0.9996 
##  wt,W3 - mut,W6   0.02409 0.01025 77.5  2.350  0.3261 
##  wt,W3 - wt,W9   -0.03806 0.01135 75.4 -3.353  0.0323 
##  wt,W3 - het,W9  -0.03691 0.00982 77.9 -3.760  0.0094 
##  wt,W3 - mut,W9  -0.00130 0.01074 77.8 -0.121  1.0000 
##  het,W3 - mut,W3  0.02938 0.01073 77.9  2.740  0.1513 
##  het,W3 - wt,W6  -0.00892 0.00868 77.7 -1.028  0.9819 
##  het,W3 - het,W6 -0.00271 0.00749 74.4 -0.362  1.0000 
##  het,W3 - mut,W6  0.02693 0.00868 77.6  3.103  0.0631 
##  het,W3 - wt,W9  -0.03522 0.01012 77.9 -3.481  0.0221 
##  het,W3 - het,W9 -0.03407 0.00800 71.6 -4.260  0.0019 
##  het,W3 - mut,W9  0.00154 0.00924 77.9  0.166  1.0000 
##  mut,W3 - wt,W6  -0.03831 0.01088 77.7 -3.521  0.0196 
##  mut,W3 - het,W6 -0.03210 0.01005 77.8 -3.195  0.0494 
##  mut,W3 - mut,W6 -0.00245 0.01078 77.1 -0.228  1.0000 
##  mut,W3 - wt,W9  -0.06460 0.01206 78.0 -5.356  <.0001 
##  mut,W3 - het,W9 -0.06346 0.01047 78.0 -6.061  <.0001 
##  mut,W3 - mut,W9 -0.02784 0.01121 76.3 -2.484  0.2562 
##  wt,W6 - het,W6   0.00621 0.00782 77.0  0.794  0.9967 
##  wt,W6 - mut,W6   0.03585 0.00887 76.9  4.042  0.0038 
##  wt,W6 - wt,W9   -0.02630 0.00959 41.9 -2.741  0.1647 
##  wt,W6 - het,W9  -0.02515 0.00836 77.7 -3.008  0.0804 
##  wt,W6 - mut,W9   0.01046 0.00942 77.6  1.110  0.9710 
##  het,W6 - mut,W6  0.02964 0.00782 77.0  3.789  0.0086 
##  het,W6 - wt,W9  -0.03251 0.00940 78.0 -3.459  0.0235 
##  het,W6 - het,W9 -0.03136 0.00669 38.2 -4.686  0.0011 
##  het,W6 - mut,W9  0.00425 0.00845 77.7  0.503  0.9999 
##  mut,W6 - wt,W9  -0.06215 0.01028 78.0 -6.044  <.0001 
##  mut,W6 - het,W9 -0.06100 0.00836 77.7 -7.296  <.0001 
##  mut,W6 - mut,W9 -0.02539 0.00867 36.3 -2.929  0.1149 
##  wt,W9 - het,W9   0.00115 0.00985 77.9  0.117  1.0000 
##  wt,W9 - mut,W9   0.03676 0.01077 78.0  3.414  0.0268 
##  het,W9 - mut,W9  0.03561 0.00895 78.0  3.980  0.0046 
## 
## Degrees-of-freedom method: kenward-roger 
## P value adjustment: tukey method for comparing a family of 9 estimates
```

```
summary(Cortexlm.Signal)
```

```
## Linear mixed model fit by REML. t-tests use Satterthwaite's method [
## lmerModLmerTest]
## Formula: Cortex.Mean/WholeBrain.Mean ~ genotype * timepoint + (1 | mouse)
##    Data: .
## 
## REML criterion at convergence: -371.7
## 
## Scaled residuals: 
##      Min       1Q   Median       3Q      Max 
## -2.18553 -0.74529  0.06817  0.64640  2.13855 
## 
## Random effects:
##  Groups   Name        Variance  Std.Dev.
##  mouse    (Intercept) 6.675e-05 0.00817 
##  Residual             3.250e-04 0.01803 
## Number of obs: 87, groups:  mouse, 55
## 
## Fixed effects:
##                          Estimate Std. Error        df t value Pr(>|t|)
## (Intercept)              0.988521   0.008050 77.673838 122.794   <2e-16
## genotypehet              0.002833   0.010001 77.835025   0.283   0.7777
## genotypemut             -0.026549   0.011935 77.790559  -2.224   0.0290
## timepointW6              0.011757   0.010079 77.372857   1.167   0.2470
## timepointW9              0.038056   0.011204 74.751679   3.397   0.0011
## genotypehet:timepointW6 -0.009043   0.012506 76.383810  -0.723   0.4718
## genotypemut:timepointW6 -0.009303   0.014681 77.129537  -0.634   0.5282
## genotypehet:timepointW9 -0.003982   0.013702 73.396935  -0.291   0.7721
## genotypemut:timepointW9 -0.010211   0.015760 75.325159  -0.648   0.5190
##                            
## (Intercept)             ***
## genotypehet                
## genotypemut             *  
## timepointW6                
## timepointW9             ** 
## genotypehet:timepointW6    
## genotypemut:timepointW6    
## genotypehet:timepointW9    
## genotypemut:timepointW9    
## ---
## Signif. codes:  0 '***' 0.001 '**' 0.01 '*' 0.05 '.' 0.1 ' ' 1
## 
## Correlation of Fixed Effects:
##             (Intr) gntyph gntypm tmpnW6 tmpnW9 gntyph:W6 gntypm:W6
## genotypehet -0.805                                                
## genotypemut -0.675  0.543                                         
## timepointW6 -0.785  0.631  0.529                                  
## timepointW9 -0.699  0.562  0.471  0.606                           
## gntypht:tW6  0.632 -0.782 -0.426 -0.806 -0.488                    
## gntypmt:tW6  0.539 -0.434 -0.798 -0.687 -0.416  0.553             
## gntypht:tW9  0.571 -0.707 -0.385 -0.495 -0.818  0.612     0.340   
## gntypmt:tW9  0.497 -0.400 -0.739 -0.431 -0.711  0.347     0.646   
##             gntyph:W9
## genotypehet          
## genotypemut          
## timepointW6          
## timepointW9          
## gntypht:tW6          
## gntypmt:tW6          
## gntypht:tW9          
## gntypmt:tW9  0.581
```

```
Hippocampuslm.Signal <- cbdf %>% lmer(Hippocampus.Mean/WholeBrain.Mean ~ genotype * 
    timepoint + (1 | mouse), data = .)

Hippocampus.SignalPairwise <- emmeans(Hippocampuslm.Signal, ~genotype * timepoint)
pairs(Hippocampus.SignalPairwise)
```

```
##  contrast         estimate      SE   df t.ratio p.value
##  wt,W3 - het,W3  -1.42e-02 0.01240 77.9 -1.142  0.9657 
##  wt,W3 - mut,W3  -1.42e-02 0.01479 77.9 -0.962  0.9882 
##  wt,W3 - wt,W6   -6.08e-02 0.01250 77.5 -4.865  0.0002 
##  wt,W3 - het,W6  -8.34e-02 0.01150 77.7 -7.246  <.0001 
##  wt,W3 - mut,W6  -4.87e-02 0.01260 77.6 -3.868  0.0067 
##  wt,W3 - wt,W9   -6.99e-02 0.01397 75.4 -5.003  0.0001 
##  wt,W3 - het,W9  -7.23e-02 0.01206 77.9 -5.994  <.0001 
##  wt,W3 - mut,W9  -7.46e-02 0.01319 77.8 -5.651  <.0001 
##  het,W3 - mut,W3 -6.54e-05 0.01318 78.0 -0.005  1.0000 
##  het,W3 - wt,W6  -4.67e-02 0.01066 77.7 -4.375  0.0012 
##  het,W3 - het,W6 -6.92e-02 0.00922 74.4 -7.503  <.0001 
##  het,W3 - mut,W6 -3.46e-02 0.01066 77.7 -3.242  0.0436 
##  het,W3 - wt,W9  -5.57e-02 0.01244 78.0 -4.477  0.0008 
##  het,W3 - het,W9 -5.82e-02 0.00985 71.7 -5.904  <.0001 
##  het,W3 - mut,W9 -6.04e-02 0.01136 78.0 -5.316  <.0001 
##  mut,W3 - wt,W6  -4.66e-02 0.01337 77.7 -3.485  0.0219 
##  mut,W3 - het,W6 -6.91e-02 0.01235 77.8 -5.600  <.0001 
##  mut,W3 - mut,W6 -3.45e-02 0.01326 77.1 -2.602  0.2028 
##  mut,W3 - wt,W9  -5.56e-02 0.01483 78.0 -3.753  0.0096 
##  mut,W3 - het,W9 -5.81e-02 0.01287 78.0 -4.514  0.0007 
##  mut,W3 - mut,W9 -6.03e-02 0.01379 76.3 -4.376  0.0012 
##  wt,W6 - het,W6  -2.25e-02 0.00961 77.2 -2.346  0.3285 
##  wt,W6 - mut,W6   1.21e-02 0.01089 77.1  1.110  0.9711 
##  wt,W6 - wt,W9   -9.06e-03 0.01186 42.2 -0.764  0.9973 
##  wt,W6 - het,W9  -1.15e-02 0.01027 77.8 -1.119  0.9695 
##  wt,W6 - mut,W9  -1.38e-02 0.01158 77.6 -1.188  0.9568 
##  het,W6 - mut,W6  3.46e-02 0.00961 77.1  3.604  0.0153 
##  het,W6 - wt,W9   1.35e-02 0.01155 78.0  1.167  0.9609 
##  het,W6 - het,W9  1.10e-02 0.00828 38.5  1.334  0.9144 
##  het,W6 - mut,W9  8.79e-03 0.01038 77.8  0.847  0.9949 
##  mut,W6 - wt,W9  -2.11e-02 0.01264 78.0 -1.673  0.7607 
##  mut,W6 - het,W9 -2.36e-02 0.01027 77.7 -2.296  0.3573 
##  mut,W6 - mut,W9 -2.58e-02 0.01073 36.6 -2.409  0.3096 
##  wt,W9 - het,W9  -2.44e-03 0.01211 77.9 -0.202  1.0000 
##  wt,W9 - mut,W9  -4.69e-03 0.01324 78.0 -0.355  1.0000 
##  het,W9 - mut,W9 -2.25e-03 0.01100 78.0 -0.205  1.0000 
## 
## Degrees-of-freedom method: kenward-roger 
## P value adjustment: tukey method for comparing a family of 9 estimates
```

```
summary(Hippocampuslm.Signal)
```

```
## Linear mixed model fit by REML. t-tests use Satterthwaite's method [
## lmerModLmerTest]
## Formula: Hippocampus.Mean/WholeBrain.Mean ~ genotype * timepoint + (1 |  
##     mouse)
##    Data: .
## 
## REML criterion at convergence: -339.5
## 
## Scaled residuals: 
##     Min      1Q  Median      3Q     Max 
## -2.0216 -0.6036 -0.1112  0.5111  2.5699 
## 
## Random effects:
##  Groups   Name        Variance  Std.Dev.
##  mouse    (Intercept) 9.236e-05 0.009611
##  Residual             4.985e-04 0.022328
## Number of obs: 87, groups:  mouse, 55
## 
## Fixed effects:
##                          Estimate Std. Error        df t value Pr(>|t|)
## (Intercept)              0.919806   0.009892 77.749090  92.982  < 2e-16
## genotypehet              0.014158   0.012291 77.875341   1.152    0.253
## genotypemut              0.014223   0.014667 77.840471   0.970    0.335
## timepointW6              0.060810   0.012395 77.356472   4.906 5.04e-06
## timepointW9              0.069870   0.013788 74.998210   5.067 2.81e-06
## genotypehet:timepointW6  0.008389   0.015385 76.434720   0.545    0.587
## genotypemut:timepointW6 -0.026310   0.018057 77.132676  -1.457    0.149
## genotypehet:timepointW9 -0.011717   0.016867 73.754813  -0.695    0.489
## genotypemut:timepointW9 -0.009531   0.019393 75.505114  -0.491    0.625
##                            
## (Intercept)             ***
## genotypehet                
## genotypemut                
## timepointW6             ***
## timepointW9             ***
## genotypehet:timepointW6    
## genotypemut:timepointW6    
## genotypehet:timepointW9    
## genotypemut:timepointW9    
## ---
## Signif. codes:  0 '***' 0.001 '**' 0.01 '*' 0.05 '.' 0.1 ' ' 1
## 
## Correlation of Fixed Effects:
##             (Intr) gntyph gntypm tmpnW6 tmpnW9 gntyph:W6 gntypm:W6
## genotypehet -0.805                                                
## genotypemut -0.674  0.543                                         
## timepointW6 -0.785  0.632  0.530                                  
## timepointW9 -0.699  0.563  0.472  0.602                           
## gntypht:tW6  0.633 -0.783 -0.427 -0.806 -0.485                    
## gntypmt:tW6  0.539 -0.434 -0.799 -0.686 -0.413  0.553             
## gntypht:tW9  0.572 -0.708 -0.386 -0.492 -0.817  0.608     0.338   
## gntypmt:tW9  0.497 -0.400 -0.739 -0.428 -0.711  0.345     0.642   
##             gntyph:W9
## genotypehet          
## genotypemut          
## timepointW6          
## timepointW9          
## gntypht:tW6          
## gntypmt:tW6          
## gntypht:tW9          
## gntypmt:tW9  0.581
```

```
Hindbrainlm.Signal <- cbdf %>% lmer(Hindbrain.Mean/WholeBrain.Mean ~ genotype * 
    timepoint + (1 | mouse), data = .)

Hindbrain.SignalPairwise <- emmeans(Hindbrainlm.Signal, ~genotype * timepoint)
pairs(Hindbrain.SignalPairwise)
```

```
##  contrast        estimate     SE   df t.ratio p.value
##  wt,W3 - het,W3   0.00537 0.0149 78.0  0.360  1.0000 
##  wt,W3 - mut,W3  -0.01928 0.0178 78.0 -1.084  0.9749 
##  wt,W3 - wt,W6    0.09572 0.0151 76.7  6.339  <.0001 
##  wt,W3 - het,W6   0.09686 0.0138 78.0  7.013  <.0001 
##  wt,W3 - mut,W6   0.07739 0.0151 78.0  5.120  0.0001 
##  wt,W3 - wt,W9    0.10606 0.0169 75.5  6.259  <.0001 
##  wt,W3 - het,W9   0.10920 0.0145 78.0  7.530  <.0001 
##  wt,W3 - mut,W9   0.11929 0.0158 78.0  7.527  <.0001 
##  het,W3 - mut,W3 -0.02465 0.0159 78.0 -1.554  0.8261 
##  het,W3 - wt,W6   0.09034 0.0128 78.0  7.056  <.0001 
##  het,W3 - het,W6  0.09149 0.0112 74.2  8.158  <.0001 
##  het,W3 - mut,W6  0.07202 0.0128 78.0  5.625  <.0001 
##  het,W3 - wt,W9   0.10069 0.0150 78.0  6.719  <.0001 
##  het,W3 - het,W9  0.10383 0.0120 72.4  8.638  <.0001 
##  het,W3 - mut,W9  0.11392 0.0137 78.0  8.339  <.0001 
##  mut,W3 - wt,W6   0.11499 0.0161 78.0  7.162  <.0001 
##  mut,W3 - het,W6  0.11614 0.0148 78.0  7.828  <.0001 
##  mut,W3 - mut,W6  0.09666 0.0160 76.5  6.028  <.0001 
##  mut,W3 - wt,W9   0.12534 0.0178 78.0  7.024  <.0001 
##  mut,W3 - het,W9  0.12848 0.0155 78.0  8.300  <.0001 
##  mut,W3 - mut,W9  0.13856 0.0167 75.9  8.293  <.0001 
##  wt,W6 - het,W6   0.00115 0.0115 78.0  0.100  1.0000 
##  wt,W6 - mut,W6  -0.01833 0.0130 78.0 -1.405  0.8925 
##  wt,W6 - wt,W9    0.01035 0.0150 45.6  0.689  0.9987 
##  wt,W6 - het,W9   0.01348 0.0123 78.0  1.094  0.9735 
##  wt,W6 - mut,W9   0.02357 0.0139 78.0  1.698  0.7460 
##  het,W6 - mut,W6 -0.01947 0.0115 78.0 -1.693  0.7492 
##  het,W6 - wt,W9   0.00920 0.0139 78.0  0.662  0.9991 
##  het,W6 - het,W9  0.01234 0.0105 41.5  1.171  0.9583 
##  het,W6 - mut,W9  0.02243 0.0125 78.0  1.801  0.6815 
##  mut,W6 - wt,W9   0.02867 0.0152 78.0  1.888  0.6241 
##  mut,W6 - het,W9  0.03181 0.0123 78.0  2.581  0.2115 
##  mut,W6 - mut,W9  0.04190 0.0137 39.2  3.058  0.0845 
##  wt,W9 - het,W9   0.00314 0.0146 78.0  0.215  1.0000 
##  wt,W9 - mut,W9   0.01323 0.0159 78.0  0.831  0.9955 
##  het,W9 - mut,W9  0.01009 0.0132 78.0  0.763  0.9975 
## 
## Degrees-of-freedom method: kenward-roger 
## P value adjustment: tukey method for comparing a family of 9 estimates
```

```
summary(Hindbrainlm.Signal)
```

```
## Linear mixed model fit by REML. t-tests use Satterthwaite's method [
## lmerModLmerTest]
## Formula: Hindbrain.Mean/WholeBrain.Mean ~ genotype * timepoint + (1 |  
##     mouse)
##    Data: .
## 
## REML criterion at convergence: -310.8
## 
## Scaled residuals: 
##      Min       1Q   Median       3Q      Max 
## -1.65162 -0.78187 -0.08674  0.65335  2.85692 
## 
## Random effects:
##  Groups   Name        Variance  Std.Dev.
##  mouse    (Intercept) 1.986e-05 0.004456
##  Residual             8.262e-04 0.028743
## Number of obs: 87, groups:  mouse, 55
## 
## Fixed effects:
##                          Estimate Std. Error        df t value Pr(>|t|)
## (Intercept)              0.996603   0.011874 77.996979  83.935  < 2e-16
## genotypehet             -0.005372   0.014760 77.998903  -0.364    0.717
## genotypemut              0.019275   0.017611 77.998403   1.094    0.277
## timepointW6             -0.095716   0.014997 76.874542  -6.382 1.20e-08
## timepointW9             -0.106063   0.016758 75.774179  -6.329 1.58e-08
## genotypehet:timepointW6  0.004226   0.018660 76.210273   0.226    0.821
## genotypemut:timepointW6 -0.000947   0.021861 76.763576  -0.043    0.966
## genotypehet:timepointW9  0.002234   0.020542 74.968611   0.109    0.914
## genotypemut:timepointW9 -0.032502   0.023553 75.965982  -1.380    0.172
##                            
## (Intercept)             ***
## genotypehet                
## genotypemut                
## timepointW6             ***
## timepointW9             ***
## genotypehet:timepointW6    
## genotypemut:timepointW6    
## genotypehet:timepointW9    
## genotypemut:timepointW9    
## ---
## Signif. codes:  0 '***' 0.001 '**' 0.01 '*' 0.05 '.' 0.1 ' ' 1
## 
## Correlation of Fixed Effects:
##             (Intr) gntyph gntypm tmpnW6 tmpnW9 gntyph:W6 gntypm:W6
## genotypehet -0.804                                                
## genotypemut -0.674  0.542                                         
## timepointW6 -0.790  0.635  0.533                                  
## timepointW9 -0.706  0.568  0.476  0.565                           
## gntypht:tW6  0.635 -0.789 -0.428 -0.804 -0.454                    
## gntypmt:tW6  0.542 -0.436 -0.804 -0.686 -0.388  0.551             
## gntypht:tW9  0.576 -0.715 -0.388 -0.461 -0.816  0.572     0.316   
## gntypmt:tW9  0.502 -0.404 -0.745 -0.402 -0.712  0.323     0.607   
##             gntyph:W9
## genotypehet          
## genotypemut          
## timepointW6          
## timepointW9          
## gntypht:tW6          
## gntypmt:tW6          
## gntypht:tW9          
## gntypmt:tW9  0.580
```

```
Midbrainlm.Signal <- cbdf %>% lmer(Midbrain.Mean/WholeBrain.Mean ~ genotype * 
    timepoint + (1 | mouse), data = .)

Midbrain.SignalPairwise <- emmeans(Midbrainlm.Signal, ~genotype * timepoint)
pairs(Midbrain.SignalPairwise)
```

```
##  contrast         estimate      SE   df t.ratio p.value
##  wt,W3 - het,W3   0.003450 0.01280 77.2  0.269  1.0000 
##  wt,W3 - mut,W3  -0.032325 0.01529 77.1 -2.114  0.4717 
##  wt,W3 - wt,W6   -0.012141 0.01277 77.8 -0.951  0.9890 
##  wt,W3 - het,W6  -0.011224 0.01194 76.2 -0.940  0.9898 
##  wt,W3 - mut,W6  -0.047009 0.01310 75.5 -3.588  0.0162 
##  wt,W3 - wt,W9   -0.031796 0.01402 74.4 -2.268  0.3743 
##  wt,W3 - het,W9  -0.032138 0.01244 77.4 -2.584  0.2103 
##  wt,W3 - mut,W9  -0.082351 0.01364 77.0 -6.039  <.0001 
##  het,W3 - mut,W3 -0.035774 0.01358 77.6 -2.634  0.1899 
##  het,W3 - wt,W6  -0.015591 0.01106 76.2 -1.410  0.8908 
##  het,W3 - het,W6 -0.014674 0.00924 73.1 -1.588  0.8082 
##  het,W3 - mut,W6 -0.050459 0.01106 76.1 -4.562  0.0006 
##  het,W3 - wt,W9  -0.035245 0.01266 77.8 -2.784  0.1371 
##  het,W3 - het,W9 -0.035588 0.00975 68.7 -3.650  0.0141 
##  het,W3 - mut,W9 -0.085801 0.01169 77.6 -7.340  <.0001 
##  mut,W3 - wt,W6   0.020183 0.01386 76.4  1.456  0.8719 
##  mut,W3 - het,W6  0.021100 0.01277 76.9  1.652  0.7730 
##  mut,W3 - mut,W6 -0.014684 0.01349 77.3 -1.089  0.9742 
##  mut,W3 - wt,W9   0.000529 0.01517 78.0  0.035  1.0000 
##  mut,W3 - het,W9  0.000186 0.01324 77.7  0.014  1.0000 
##  mut,W3 - mut,W9 -0.050027 0.01393 76.0 -3.590  0.0161 
##  wt,W6 - het,W6   0.000917 0.01005 73.7  0.091  1.0000 
##  wt,W6 - mut,W6  -0.034868 0.01141 73.2 -3.057  0.0718 
##  wt,W6 - wt,W9   -0.019654 0.01085 36.6 -1.812  0.6741 
##  wt,W6 - het,W9  -0.019997 0.01063 76.5 -1.880  0.6290 
##  wt,W6 - mut,W9  -0.070210 0.01202 75.9 -5.843  <.0001 
##  het,W6 - mut,W6 -0.035784 0.01005 73.6 -3.560  0.0178 
##  het,W6 - wt,W9  -0.020571 0.01179 78.0 -1.745  0.7171 
##  het,W6 - het,W9 -0.020914 0.00750 34.1 -2.788  0.1561 
##  het,W6 - mut,W9 -0.071127 0.01074 76.7 -6.623  <.0001 
##  mut,W6 - wt,W9   0.015213 0.01296 77.8  1.174  0.9597 
##  mut,W6 - het,W9  0.014871 0.01063 76.4  1.398  0.8951 
##  mut,W6 - mut,W9 -0.035342 0.00967 32.7 -3.655  0.0220 
##  wt,W9 - het,W9  -0.000343 0.01229 77.7 -0.028  1.0000 
##  wt,W9 - mut,W9  -0.050556 0.01350 78.0 -3.744  0.0099 
##  het,W9 - mut,W9 -0.050213 0.01129 77.8 -4.449  0.0009 
## 
## Degrees-of-freedom method: kenward-roger 
## P value adjustment: tukey method for comparing a family of 9 estimates
```

```
summary(Midbrainlm.Signal)
```

```
## Linear mixed model fit by REML. t-tests use Satterthwaite's method [
## lmerModLmerTest]
## Formula: 
## Midbrain.Mean/WholeBrain.Mean ~ genotype * timepoint + (1 | mouse)
##    Data: .
## 
## REML criterion at convergence: -335.9
## 
## Scaled residuals: 
##      Min       1Q   Median       3Q      Max 
## -2.28966 -0.56976  0.05702  0.40532  1.77051 
## 
## Random effects:
##  Groups   Name        Variance  Std.Dev.
##  mouse    (Intercept) 0.0002600 0.01612 
##  Residual             0.0003946 0.01986 
## Number of obs: 87, groups:  mouse, 55
## 
## Fixed effects:
##                          Estimate Std. Error        df t value Pr(>|t|)
## (Intercept)              1.065466   0.010235 76.591781 104.096   <2e-16
## genotypehet             -0.003450   0.012682 77.174202  -0.272    0.786
## genotypemut              0.032325   0.015146 77.043627   2.134    0.036
## timepointW6              0.012141   0.012602 77.817045   0.963    0.338
## timepointW9              0.031796   0.013798 74.382658   2.304    0.024
## genotypehet:timepointW6  0.002533   0.015535 76.869776   0.163    0.871
## genotypemut:timepointW6  0.002543   0.018317 77.569841   0.139    0.890
## genotypehet:timepointW9  0.003792   0.016798 72.712016   0.226    0.822
## genotypemut:timepointW9  0.018231   0.019460 75.241945   0.937    0.352
##                            
## (Intercept)             ***
## genotypehet                
## genotypemut             *  
## timepointW6                
## timepointW9             *  
## genotypehet:timepointW6    
## genotypemut:timepointW6    
## genotypehet:timepointW9    
## genotypemut:timepointW9    
## ---
## Signif. codes:  0 '***' 0.001 '**' 0.01 '*' 0.05 '.' 0.1 ' ' 1
## 
## Correlation of Fixed Effects:
##             (Intr) gntyph gntypm tmpnW6 tmpnW9 gntyph:W6 gntypm:W6
## genotypehet -0.807                                                
## genotypemut -0.676  0.545                                         
## timepointW6 -0.772  0.623  0.522                                  
## timepointW9 -0.690  0.557  0.466  0.672                           
## gntypht:tW6  0.626 -0.767 -0.423 -0.811 -0.545                    
## gntypmt:tW6  0.531 -0.429 -0.786 -0.688 -0.462  0.558             
## gntypht:tW9  0.567 -0.694 -0.383 -0.552 -0.821  0.675     0.380   
## gntypmt:tW9  0.489 -0.395 -0.729 -0.476 -0.709  0.387     0.710   
##             gntyph:W9
## genotypehet          
## genotypemut          
## timepointW6          
## timepointW9          
## gntypht:tW6          
## gntypmt:tW6          
## gntypht:tW9          
## gntypmt:tW9  0.582
```

```
Thalamuslm.Signal <- cbdf %>% lmer(Thalamus.Mean/WholeBrain.Mean ~ genotype * 
    timepoint + (1 | mouse), data = .)

Thalamus.SignalPairwise <- emmeans(Thalamuslm.Signal, ~genotype * timepoint)
pairs(Thalamus.SignalPairwise)
```

```
##  contrast         estimate      SE   df t.ratio p.value
##  wt,W3 - het,W3  -0.013433 0.01322 77.0  -1.016 0.9832 
##  wt,W3 - mut,W3  -0.021881 0.01580 76.9  -1.385 0.9001 
##  wt,W3 - wt,W6    0.005567 0.01314 77.8   0.424 1.0000 
##  wt,W3 - het,W6  -0.010471 0.01236 75.7  -0.848 0.9949 
##  wt,W3 - mut,W6  -0.058037 0.01357 74.9  -4.278 0.0017 
##  wt,W3 - wt,W9    0.031635 0.01433 73.9   2.207 0.4121 
##  wt,W3 - het,W9   0.005353 0.01284 77.2   0.417 1.0000 
##  wt,W3 - mut,W9  -0.121877 0.01409 76.7  -8.652 <.0001 
##  het,W3 - mut,W3 -0.008449 0.01402 77.5  -0.603 0.9995 
##  het,W3 - wt,W6   0.019000 0.01144 75.7   1.660 0.7680 
##  het,W3 - het,W6  0.002962 0.00944 72.2   0.314 1.0000 
##  het,W3 - mut,W6 -0.044604 0.01144 75.6  -3.898 0.0062 
##  het,W3 - wt,W9   0.045068 0.01300 77.9   3.466 0.0231 
##  het,W3 - het,W9  0.018785 0.00992 67.3   1.893 0.6209 
##  het,W3 - mut,W9 -0.108444 0.01206 77.4  -8.995 <.0001 
##  mut,W3 - wt,W6   0.027449 0.01434 76.0   1.914 0.6064 
##  mut,W3 - het,W6  0.011410 0.01320 76.7   0.864 0.9942 
##  mut,W3 - mut,W6 -0.036156 0.01386 77.1  -2.609 0.1998 
##  mut,W3 - wt,W9   0.053517 0.01561 78.0   3.427 0.0258 
##  mut,W3 - het,W9  0.027234 0.01365 77.6   1.995 0.5515 
##  mut,W3 - mut,W9 -0.099996 0.01428 75.7  -7.002 <.0001 
##  wt,W6 - het,W6  -0.016038 0.01043 72.8  -1.538 0.8337 
##  wt,W6 - mut,W6  -0.063604 0.01184 72.2  -5.373 <.0001 
##  wt,W6 - wt,W9    0.026068 0.01079 35.4   2.416 0.3067 
##  wt,W6 - het,W9  -0.000215 0.01099 76.0  -0.020 1.0000 
##  wt,W6 - mut,W9  -0.127444 0.01243 75.3 -10.252 <.0001 
##  het,W6 - mut,W6 -0.047566 0.01043 72.6  -4.561 0.0007 
##  het,W6 - wt,W9   0.042106 0.01212 78.0   3.474 0.0225 
##  het,W6 - het,W9  0.015824 0.00745 33.2   2.125 0.4746 
##  het,W6 - mut,W9 -0.111406 0.01110 76.2 -10.040 <.0001 
##  mut,W6 - wt,W9   0.089672 0.01335 77.7   6.716 <.0001 
##  mut,W6 - het,W9  0.063390 0.01099 76.0   5.767 <.0001 
##  mut,W6 - mut,W9 -0.063840 0.00959 31.9  -6.659 <.0001 
##  wt,W9 - het,W9  -0.026282 0.01261 77.7  -2.085 0.4912 
##  wt,W9 - mut,W9  -0.153512 0.01388 78.0 -11.059 <.0001 
##  het,W9 - mut,W9 -0.127230 0.01163 77.7 -10.941 <.0001 
## 
## Degrees-of-freedom method: kenward-roger 
## P value adjustment: tukey method for comparing a family of 9 estimates
```

```
summary(Thalamuslm.Signal)
```

```
## Linear mixed model fit by REML. t-tests use Satterthwaite's method [
## lmerModLmerTest]
## Formula: 
## Thalamus.Mean/WholeBrain.Mean ~ genotype * timepoint + (1 | mouse)
##    Data: .
## 
## REML criterion at convergence: -331.5
## 
## Scaled residuals: 
##      Min       1Q   Median       3Q      Max 
## -2.29104 -0.55029 -0.01046  0.36199  1.98607 
## 
## Random effects:
##  Groups   Name        Variance  Std.Dev.
##  mouse    (Intercept) 0.0003231 0.01798 
##  Residual             0.0003857 0.01964 
## Number of obs: 87, groups:  mouse, 55
## 
## Fixed effects:
##                          Estimate Std. Error        df t value Pr(>|t|)
## (Intercept)              1.090608   0.010577 75.378890 103.111  < 2e-16
## genotypehet              0.013433   0.013092 76.429891   1.026   0.3081
## genotypemut              0.021881   0.015639 76.226170   1.399   0.1658
## timepointW6             -0.005567   0.012945 77.634457  -0.430   0.6683
## timepointW9             -0.031635   0.014098 71.757158  -2.244   0.0279
## genotypehet:timepointW6  0.002606   0.015922 75.902065   0.164   0.8704
## genotypemut:timepointW6  0.041723   0.018800 77.155765   2.219   0.0294
## genotypehet:timepointW9  0.012850   0.017136 69.010497   0.750   0.4559
## genotypemut:timepointW9  0.131631   0.019900 73.133425   6.615 5.32e-09
##                            
## (Intercept)             ***
## genotypehet                
## genotypemut                
## timepointW6                
## timepointW9             *  
## genotypehet:timepointW6    
## genotypemut:timepointW6 *  
## genotypehet:timepointW9    
## genotypemut:timepointW9 ***
## ---
## Signif. codes:  0 '***' 0.001 '**' 0.01 '*' 0.05 '.' 0.1 ' ' 1
## 
## Correlation of Fixed Effects:
##             (Intr) gntyph gntypm tmpnW6 tmpnW9 gntyph:W6 gntypm:W6
## genotypehet -0.808                                                
## genotypemut -0.676  0.546                                         
## timepointW6 -0.767  0.620  0.519                                  
## timepointW9 -0.688  0.556  0.465  0.690                           
## gntypht:tW6  0.624 -0.761 -0.422 -0.813 -0.561                    
## gntypmt:tW6  0.528 -0.427 -0.781 -0.689 -0.475  0.560             
## gntypht:tW9  0.566 -0.691 -0.383 -0.568 -0.823  0.693     0.391   
## gntypmt:tW9  0.487 -0.394 -0.726 -0.489 -0.708  0.398     0.727   
##             gntyph:W9
## genotypehet          
## genotypemut          
## timepointW6          
## timepointW9          
## gntypht:tW6          
## gntypmt:tW6          
## gntypht:tW9          
## gntypmt:tW9  0.583
```

```
Hypothalamuslm.Signal <- cbdf %>% lmer(Hypothalamus.Mean/WholeBrain.Mean ~ genotype * 
    timepoint + (1 | mouse), data = .)

Hypothalamus.SignalPairwise <- emmeans(Hypothalamuslm.Signal, ~genotype * timepoint)
pairs(Hypothalamus.SignalPairwise)
```

```
##  contrast        estimate     SE   df t.ratio p.value
##  wt,W3 - het,W3  -0.02632 0.0186 77.2 -1.414  0.8891 
##  wt,W3 - mut,W3   0.03634 0.0222 77.1  1.635  0.7826 
##  wt,W3 - wt,W6   -0.06611 0.0186 77.8 -3.557  0.0176 
##  wt,W3 - het,W6  -0.12364 0.0174 76.3 -7.125  <.0001 
##  wt,W3 - mut,W6  -0.05169 0.0190 75.7 -2.715  0.1600 
##  wt,W3 - wt,W9   -0.08301 0.0204 74.6 -4.062  0.0036 
##  wt,W3 - het,W9  -0.09424 0.0181 77.5 -5.211  0.0001 
##  wt,W3 - mut,W9  -0.00519 0.0198 77.0 -0.262  1.0000 
##  het,W3 - mut,W3  0.06266 0.0198 77.6  3.172  0.0526 
##  het,W3 - wt,W6  -0.03979 0.0161 76.3 -2.475  0.2605 
##  het,W3 - het,W6 -0.09732 0.0135 73.3 -7.223  <.0001 
##  het,W3 - mut,W6 -0.02537 0.0161 76.2 -1.578  0.8133 
##  het,W3 - wt,W9  -0.05669 0.0184 77.9 -3.075  0.0678 
##  het,W3 - het,W9 -0.06792 0.0142 69.0 -4.772  0.0003 
##  het,W3 - mut,W9  0.02113 0.0170 77.6  1.243  0.9442 
##  mut,W3 - wt,W6  -0.10245 0.0201 76.5 -5.084  0.0001 
##  mut,W3 - het,W6 -0.15998 0.0186 77.0 -8.616  <.0001 
##  mut,W3 - mut,W6 -0.08803 0.0196 77.3 -4.483  0.0008 
##  mut,W3 - wt,W9  -0.11935 0.0221 78.0 -5.405  <.0001 
##  mut,W3 - het,W9 -0.13058 0.0193 77.7 -6.782  <.0001 
##  mut,W3 - mut,W9 -0.04153 0.0203 76.1 -2.046  0.5172 
##  wt,W6 - het,W6  -0.05753 0.0146 74.0 -3.941  0.0054 
##  wt,W6 - mut,W6   0.01442 0.0166 73.5  0.870  0.9939 
##  wt,W6 - wt,W9   -0.01690 0.0159 37.0 -1.061  0.9763 
##  wt,W6 - het,W9  -0.02813 0.0155 76.6 -1.820  0.6692 
##  wt,W6 - mut,W9   0.06092 0.0175 76.1  3.488  0.0218 
##  het,W6 - mut,W6  0.07195 0.0146 73.9  4.929  0.0002 
##  het,W6 - wt,W9   0.04063 0.0172 78.0  2.367  0.3166 
##  het,W6 - het,W9  0.02940 0.0110 34.4  2.667  0.1974 
##  het,W6 - mut,W9  0.11845 0.0156 76.8  7.587  <.0001 
##  mut,W6 - wt,W9  -0.03132 0.0189 77.9 -1.660  0.7681 
##  mut,W6 - het,W9 -0.04255 0.0155 76.6 -2.752  0.1474 
##  mut,W6 - mut,W9  0.04650 0.0142 33.0  3.271  0.0555 
##  wt,W9 - het,W9  -0.01123 0.0179 77.7 -0.627  0.9994 
##  wt,W9 - mut,W9   0.07782 0.0197 78.0  3.959  0.0049 
##  het,W9 - mut,W9  0.08905 0.0164 77.8  5.423  <.0001 
## 
## Degrees-of-freedom method: kenward-roger 
## P value adjustment: tukey method for comparing a family of 9 estimates
```

```
summary(Hypothalamuslm.Signal)
```

```
## Linear mixed model fit by REML. t-tests use Satterthwaite's method [
## lmerModLmerTest]
## Formula: Hypothalamus.Mean/WholeBrain.Mean ~ genotype * timepoint + (1 |  
##     mouse)
##    Data: .
## 
## REML criterion at convergence: -277.4
## 
## Scaled residuals: 
##      Min       1Q   Median       3Q      Max 
## -2.95894 -0.44501  0.06124  0.49483  1.50100 
## 
## Random effects:
##  Groups   Name        Variance  Std.Dev.
##  mouse    (Intercept) 0.0005238 0.02289 
##  Residual             0.0008545 0.02923 
## Number of obs: 87, groups:  mouse, 55
## 
## Fixed effects:
##                         Estimate Std. Error       df t value Pr(>|t|)    
## (Intercept)              1.00183    0.01488 76.87965  67.330  < 2e-16 ***
## genotypehet              0.02632    0.01844 77.34750   1.427 0.157586    
## genotypemut             -0.03634    0.02202 77.23926  -1.650 0.102945    
## timepointW6              0.06611    0.01835 77.84719   3.603 0.000552 ***
## timepointW9              0.08301    0.02012 75.00640   4.126 9.46e-05 ***
## genotypehet:timepointW6  0.03121    0.02263 77.06927   1.379 0.171841    
## genotypemut:timepointW6  0.02193    0.02667 77.64633   0.822 0.413609    
## genotypehet:timepointW9 -0.01509    0.02450 73.61149  -0.616 0.539977    
## genotypemut:timepointW9 -0.04148    0.02837 75.72493  -1.462 0.147841    
## ---
## Signif. codes:  0 '***' 0.001 '**' 0.01 '*' 0.05 '.' 0.1 ' ' 1
## 
## Correlation of Fixed Effects:
##             (Intr) gntyph gntypm tmpnW6 tmpnW9 gntyph:W6 gntypm:W6
## genotypehet -0.807                                                
## genotypemut -0.676  0.545                                         
## timepointW6 -0.773  0.624  0.522                                  
## timepointW9 -0.691  0.557  0.467  0.667                           
## gntypht:tW6  0.627 -0.768 -0.424 -0.811 -0.541                    
## gntypmt:tW6  0.532 -0.429 -0.787 -0.688 -0.459  0.558             
## gntypht:tW9  0.567 -0.695 -0.383 -0.547 -0.821  0.670     0.377   
## gntypmt:tW9  0.490 -0.395 -0.730 -0.473 -0.709  0.383     0.705   
##             gntyph:W9
## genotypehet          
## genotypemut          
## timepointW6          
## timepointW9          
## gntypht:tW6          
## gntypmt:tW6          
## gntypht:tW9          
## gntypmt:tW9  0.582
```

# Figure 1 - Quantitative mass, brain volume, and brain signal trends

## Mass

```
animalmass <- gf %>% ggplot() + aes(x = time, y = mass, color = genotype) + 
    stat_summary(fun.data = mean_cl_boot, geom = "line") + stat_summary(fun.data = mean_cl_boot) + 
    ylab(bquote(bold("Body Mass (g)"))) + xlab("Age (Weeks)") + scale_color_discrete("Genotype", 
    labels = labeller) + scale_x_continuous(breaks = c(3, 6, 9)) + scale_y_continuous(breaks = pretty_breaks(n = 6), 
    limits = c(0, 30)) + plottheme

animalmass
```

```
ggsave("animalmass.png", animalmass, scale = 3, dpi = "retina", width = 1.5, 
    height = 1)
```

```
AnimalMasslm <- gf %>% lmer(mass ~ genotype * timepoint + (1 | mouse), data = .)

AnimalMasslm.Pairwise <- emmeans(AnimalMasslm, ~genotype * timepoint)
pairs(AnimalMasslm.Pairwise)
```

```
##  contrast        estimate    SE   df t.ratio p.value
##  wt,W3 - het,W3     0.466 0.998 76.8   0.467 0.9999 
##  wt,W3 - mut,W3     2.202 1.193 76.7   1.846 0.6522 
##  wt,W3 - wt,W6     -9.659 0.987 77.6  -9.786 <.0001 
##  wt,W3 - het,W6    -9.454 0.935 75.4 -10.115 <.0001 
##  wt,W3 - mut,W6    -6.472 1.027 74.4  -6.300 <.0001 
##  wt,W3 - wt,W9    -12.190 1.069 73.2 -11.406 <.0001 
##  wt,W3 - het,W9    -9.864 0.968 77.0 -10.189 <.0001 
##  wt,W3 - mut,W9    -3.257 1.064 76.4  -3.062 0.0704 
##  het,W3 - mut,W3    1.735 1.057 77.4   1.642 0.7787 
##  het,W3 - wt,W6   -10.125 0.866 75.2 -11.698 <.0001 
##  het,W3 - het,W6   -9.920 0.703 70.8 -14.109 <.0001 
##  het,W3 - mut,W6   -6.939 0.866 75.1  -8.016 <.0001 
##  het,W3 - wt,W9   -12.657 0.975 77.9 -12.980 <.0001 
##  het,W3 - het,W9  -10.330 0.736 65.6 -14.041 <.0001 
##  het,W3 - mut,W9   -3.724 0.909 77.3  -4.099 0.0031 
##  mut,W3 - wt,W6   -11.860 1.084 75.7 -10.937 <.0001 
##  mut,W3 - het,W6  -11.656 0.997 76.5 -11.689 <.0001 
##  mut,W3 - mut,W6   -8.674 1.039 76.7  -8.352 <.0001 
##  mut,W3 - wt,W9   -14.392 1.174 78.0 -12.262 <.0001 
##  mut,W3 - het,W9  -12.066 1.029 77.5 -11.731 <.0001 
##  mut,W3 - mut,W9   -5.459 1.068 75.0  -5.114 0.0001 
##  wt,W6 - het,W6     0.205 0.791 71.8   0.259 1.0000 
##  wt,W6 - mut,W6     3.186 0.899 71.1   3.544 0.0190 
##  wt,W6 - wt,W9     -2.532 0.780 34.1  -3.248 0.0576 
##  wt,W6 - het,W9    -0.205 0.831 75.5  -0.247 1.0000 
##  wt,W6 - mut,W9     6.401 0.940 74.7   6.807 <.0001 
##  het,W6 - mut,W6    2.981 0.792 71.6   3.767 0.0096 
##  het,W6 - wt,W9    -2.737 0.910 78.0  -3.007 0.0804 
##  het,W6 - het,W9   -0.410 0.537 32.2  -0.763 0.9972 
##  het,W6 - mut,W9    6.197 0.838 75.7   7.392 <.0001 
##  mut,W6 - wt,W9    -5.718 1.005 77.6  -5.690 <.0001 
##  mut,W6 - het,W9   -3.391 0.831 75.4  -4.082 0.0034 
##  mut,W6 - mut,W9    3.215 0.691 31.1   4.655 0.0017 
##  wt,W9 - het,W9     2.327 0.944 77.7   2.464 0.2658 
##  wt,W9 - mut,W9     8.933 1.042 78.0   8.572 <.0001 
##  het,W9 - mut,W9    6.607 0.875 77.5   7.547 <.0001 
## 
## Degrees-of-freedom method: kenward-roger 
## P value adjustment: tukey method for comparing a family of 9 estimates
```

```
summary(AnimalMasslm)
```

```
## Linear mixed model fit by REML. t-tests use Satterthwaite's method [
## lmerModLmerTest]
## Formula: mass ~ genotype * timepoint + (1 | mouse)
##    Data: .
## 
## REML criterion at convergence: 342.3
## 
## Scaled residuals: 
##      Min       1Q   Median       3Q      Max 
## -1.72771 -0.31118 -0.05034  0.36755  2.62062 
## 
## Random effects:
##  Groups   Name        Variance Std.Dev.
##  mouse    (Intercept) 2.129    1.459   
##  Residual             1.990    1.411   
## Number of obs: 87, groups:  mouse, 55
## 
## Fixed effects:
##                         Estimate Std. Error      df t value Pr(>|t|)    
## (Intercept)              10.5746     0.7989 76.4878  13.236  < 2e-16 ***
## genotypehet              -0.4665     0.9877 77.0884  -0.472   0.6380    
## genotypemut              -2.2018     1.1801 76.9999  -1.866   0.0659 .  
## timepointW6               9.6586     0.9705 77.6929   9.952 1.63e-15 ***
## timepointW9              12.1905     1.0502 74.1689  11.607  < 2e-16 ***
## genotypehet:timepointW6   0.2618     1.1904 76.5737   0.220   0.8265    
## genotypemut:timepointW6  -0.9844     1.4080 77.3611  -0.699   0.4866    
## genotypehet:timepointW9  -1.8601     1.2742 72.4214  -1.460   0.1487    
## genotypemut:timepointW9  -6.7313     1.4840 74.9462  -4.536 2.14e-05 ***
## ---
## Signif. codes:  0 '***' 0.001 '**' 0.01 '*' 0.05 '.' 0.1 ' ' 1
## 
## Correlation of Fixed Effects:
##             (Intr) gntyph gntypm tmpnW6 tmpnW9 gntyph:W6 gntypm:W6
## genotypehet -0.809                                                
## genotypemut -0.677  0.548                                         
## timepointW6 -0.761  0.616  0.515                                  
## timepointW9 -0.685  0.554  0.464  0.710                           
## gntypht:tW6  0.621 -0.754 -0.420 -0.815 -0.579                    
## gntypmt:tW6  0.525 -0.425 -0.775 -0.689 -0.489  0.562             
## gntypht:tW9  0.565 -0.686 -0.382 -0.585 -0.824  0.711     0.403   
## gntypmt:tW9  0.485 -0.392 -0.723 -0.502 -0.708  0.410     0.745   
##             gntyph:W9
## genotypehet          
## genotypemut          
## timepointW6          
## timepointW9          
## gntypht:tW6          
## gntypmt:tW6          
## gntypht:tW9          
## gntypmt:tW9  0.583
```

## Brain Volume

```
wholebrainvolume <- gf %>% mutate(`Whole Brain` = rowSums(combvols.DSURQE)) %>% 
    gather(roi, volume, `Whole Brain`) %>% ggplot() + aes(x = time, y = volume, 
    color = genotype) + stat_summary(fun.data = mean_cl_boot, geom = "line") + 
    stat_summary(fun.data = mean_cl_boot) + ylab(bquote(bold("Brain Volume" ~ 
    (mm^3)))) + xlab("Age (Weeks)") + scale_color_discrete("Genotype", labels = labeller) + 
    scale_y_continuous(breaks = pretty_breaks(n = 5), limits = c(300, 450)) + 
    scale_x_continuous(breaks = c(3, 6, 9)) + plottheme


wholebrainvolume
```

## Signal

```
wholebrainsignal <- gf %>% mutate(`Whole Brain` = rowSums(combsums.DSURQE)) %>% 
    gather(roi, signal, `Whole Brain`) %>% ggplot() + aes(x = time, y = signal, 
    color = genotype) + stat_summary(fun.data = mean_cl_boot, geom = "line") + 
    stat_summary(fun.data = mean_cl_boot) + ylab(bquote(bold("Signal (a.u.)"))) + 
    xlab("Age (Weeks)") + scale_color_discrete("Genotype", labels = labeller) + 
    scale_x_continuous(breaks = c(3, 6, 9)) + plottheme

wholebrainsignal
```

```
wholebrainpervoxelsignal <- gf %>% mutate(`Whole Brain` = rowSums(combsums.DSURQE)/rowSums(combvols.DSURQE)) %>% 
    gather(roi, signal, `Whole Brain`) %>% ggplot() + aes(x = time, y = signal/1e+05, 
    color = genotype) + stat_summary(fun.data = mean_cl_boot, geom = "line") + 
    stat_summary(fun.data = mean_cl_boot) + ylab(bquote(bold("Signal per Voxel (a.u.)"))) + 
    xlab("Age (Weeks)") + scale_color_discrete("Genotype", labels = labeller) + 
    scale_y_continuous(breaks = pretty_breaks()) + scale_x_continuous(breaks = c(3, 
    6, 9)) + plottheme

wholebrainpervoxelsignal
```

```
ggsave("wholebrainsignal.png", wholebrainsignal, scale = 3, dpi = "retina", 
    width = 1.5, height = 1)
ggsave("wholebrainvolume.png", wholebrainvolume, scale = 3, dpi = "retina", 
    width = 1.5, height = 1)
ggsave("wholebrainpervoxelsignal.png", wholebrainpervoxelsignal, scale = 3, 
    dpi = "retina", width = 1.5, height = 1)
```

## Quantitative analyses - tabulated effect sizes and p-values

```
wholebrainpervoxelsignallm <- gf %>% mutate(`Whole Brain` = rowSums(combsums.DSURQE)/rowSums(combvols.DSURQE)) %>% 
    gather(roi, signal, `Whole Brain`) %>% lmer(signal ~ genotype * timepoint + 
    (1 | mouse), data = .)
```

```
## Warning in as_lmerModLT(model, devfun): Model may not have converged with 1
## eigenvalue close to zero: 6.0e-09
```

```
wholebrainpervoxelsignallm.Pairwise <- emmeans(wholebrainpervoxelsignallm, ~genotype * 
    timepoint)
pairs(wholebrainpervoxelsignallm.Pairwise)
```

```
##  contrast        estimate    SE   df t.ratio p.value
##  wt,W3 - het,W3    -30722 81699 78.0 -0.376  1.0000 
##  wt,W3 - mut,W3   -213765 97431 78.0 -2.194  0.4198 
##  wt,W3 - wt,W6     326073 82716 76.8  3.942  0.0053 
##  wt,W3 - het,W6    313832 75688 78.0  4.146  0.0026 
##  wt,W3 - mut,W6     91818 82826 78.0  1.109  0.9713 
##  wt,W3 - wt,W9     412050 92818 75.5  4.439  0.0010 
##  wt,W3 - het,W9    557697 79464 78.0  7.018  <.0001 
##  wt,W3 - mut,W9    101285 86843 78.0  1.166  0.9611 
##  het,W3 - mut,W3  -183043 86921 78.0 -2.106  0.4770 
##  het,W3 - wt,W6    356794 70165 78.0  5.085  0.0001 
##  het,W3 - het,W6   344553 61424 74.2  5.609  <.0001 
##  het,W3 - mut,W6   122539 70161 78.0  1.747  0.7161 
##  het,W3 - wt,W9    442772 82125 78.0  5.391  <.0001 
##  het,W3 - het,W9   588418 65827 72.4  8.939  <.0001 
##  het,W3 - mut,W9   132007 74861 78.0  1.763  0.7055 
##  mut,W3 - wt,W6    539837 87983 78.0  6.136  <.0001 
##  mut,W3 - het,W6   527597 81297 78.0  6.490  <.0001 
##  mut,W3 - mut,W6   305582 87852 76.5  3.478  0.0224 
##  mut,W3 - wt,W9    625815 97788 78.0  6.400  <.0001 
##  mut,W3 - het,W9   771461 84824 78.0  9.095  <.0001 
##  mut,W3 - mut,W9   315050 91531 75.9  3.442  0.0250 
##  wt,W6 - het,W6    -12241 63063 78.0 -0.194  1.0000 
##  wt,W6 - mut,W6   -234255 71473 78.0 -3.278  0.0395 
##  wt,W6 - wt,W9      85978 82072 45.5  1.048  0.9786 
##  wt,W6 - het,W9    231624 67549 78.0  3.429  0.0257 
##  wt,W6 - mut,W9   -224787 76092 78.0 -2.954  0.0918 
##  het,W6 - mut,W6  -222014 63059 78.0 -3.521  0.0196 
##  het,W6 - wt,W9     98219 76148 78.0  1.290  0.9314 
##  het,W6 - het,W9   243865 57598 41.3  4.234  0.0036 
##  het,W6 - mut,W9  -212547 68250 78.0 -3.114  0.0612 
##  mut,W6 - wt,W9    320233 83246 78.0  3.847  0.0071 
##  mut,W6 - het,W9   465879 67545 78.0  6.897  <.0001 
##  mut,W6 - mut,W9     9467 74876 39.1  0.126  1.0000 
##  wt,W9 - het,W9    145646 79902 78.0  1.823  0.6671 
##  wt,W9 - mut,W9   -310765 87244 78.0 -3.562  0.0173 
##  het,W9 - mut,W9  -456412 72415 78.0 -6.303  <.0001 
## 
## Degrees-of-freedom method: kenward-roger 
## P value adjustment: tukey method for comparing a family of 9 estimates
```

```
summary(wholebrainpervoxelsignallm)
```

```
## Linear mixed model fit by REML. t-tests use Satterthwaite's method [
## lmerModLmerTest]
## Formula: signal ~ genotype * timepoint + (1 | mouse)
##    Data: .
## 
## REML criterion at convergence: 2109.8
## 
## Scaled residuals: 
##     Min      1Q  Median      3Q     Max 
## -4.3709 -0.3664  0.0660  0.5636  1.6595 
## 
## Random effects:
##  Groups   Name        Variance  Std.Dev.
##  mouse    (Intercept) 7.578e+08  27528  
##  Residual             2.465e+10 157013  
## Number of obs: 87, groups:  mouse, 55
## 
## Fixed effects:
##                          Estimate Std. Error        df t value Pr(>|t|)
## (Intercept)             2267474.6    65069.8     145.8  34.847  < 2e-16
## genotypehet               30721.5    80890.5     146.6   0.380 0.704650
## genotypemut              213764.8    96512.6     146.4   2.215 0.028313
## timepointW6             -326072.6    82156.4     188.2  -3.969 0.000103
## timepointW9             -412050.5    91790.8     211.4  -4.489 1.18e-05
## genotypehet:timepointW6  -18480.8   102212.8     202.3  -0.181 0.856700
## genotypemut:timepointW6   20490.1   119753.2     190.8   0.171 0.864324
## genotypehet:timepointW9 -176367.6   112503.7     226.6  -1.568 0.118356
## genotypemut:timepointW9   97000.6   129011.4     207.5   0.752 0.452977
##                            
## (Intercept)             ***
## genotypehet                
## genotypemut             *  
## timepointW6             ***
## timepointW9             ***
## genotypehet:timepointW6    
## genotypemut:timepointW6    
## genotypehet:timepointW9    
## genotypemut:timepointW9    
## ---
## Signif. codes:  0 '***' 0.001 '**' 0.01 '*' 0.05 '.' 0.1 ' ' 1
## 
## Correlation of Fixed Effects:
##             (Intr) gntyph gntypm tmpnW6 tmpnW9 gntyph:W6 gntypm:W6
## genotypehet -0.804                                                
## genotypemut -0.674  0.542                                         
## timepointW6 -0.790  0.635  0.532                                  
## timepointW9 -0.705  0.567  0.476  0.567                           
## gntypht:tW6  0.635 -0.788 -0.428 -0.804 -0.456                    
## gntypmt:tW6  0.542 -0.436 -0.804 -0.686 -0.389  0.551             
## gntypht:tW9  0.576 -0.715 -0.388 -0.463 -0.816  0.574     0.317   
## gntypmt:tW9  0.502 -0.404 -0.745 -0.403 -0.711  0.324     0.608   
##             gntyph:W9
## genotypehet          
## genotypemut          
## timepointW6          
## timepointW9          
## gntypht:tW6          
## gntypmt:tW6          
## gntypht:tW9          
## gntypmt:tW9  0.581
```

# Figure 2 - Deformation-based morphometry

```
poscolours = colorRampPalette(c("blue", "red"))(255)


mincPlotSliceSeries(mincArray(anatVol),           # the anatomical volume
                    mincArray(vs_W3, "beta-genotypemut"), # pull out one column of the stats
                    anatLow=1000, anatHigh=1500,   # set anatomy thresholds
                    low=-0.1, high=-0.5,             # set stats thresholds
                    begin=72, end=-40,            # remove slices from both sides
                    mfrow = c(3,3),
                    legend="volume reduction (%)",
                    dimension = 1,
                    col = poscolours)
```

```
mincPlotSliceSeries(mincArray(anatVol),           # the anatomical volume
                    mincArray(vs_W6, "beta-genotypemut"), # pull out one column of the stats
                    anatLow=1000, anatHigh=1500,   # set anatomy thresholds
                    low=-0.1, high=-0.5,             # set stats thresholds
                    begin=72, end=-40,            # remove slices from both sides  
                    legend="volume reduction (%)",
                    mfrow = c(3,3),
                    dimension = 1,
                    col = poscolours)
```

```
mincPlotSliceSeries(mincArray(anatVol),           # the anatomical volume
                    mincArray(abs(vs_W9*100), "beta-genotypemut"), # pull out one column of the stats
                    anatLow=1000, anatHigh=1500,   # set anatomy thresholds
                    low=abs(-0.1*100), high=abs(-0.5*100),             # set stats thresholds
                    begin=72, end=-40,            # remove slices from both sides  
                    legend="volume reduction (%)",
                    mfrow = c(3,3),
                    dimension = 1,
                    col = poscolours)
```

```
poscolours = colorRampPalette(c("blue", "red"))(255)


mincPlotSliceSeries(mincArray(anatVol),           # the anatomical volume
                    mincArray(vs_W3, "beta-genotypemut"), # pull out one column of the stats
                    anatLow=1000, anatHigh=1500,   # set anatomy thresholds
                    low=-0.1, high=-0.5,             # set stats thresholds
                    begin=72, end=-40,            # remove slices from both sides
                    mfrow = c(3,3),
                    # legend="volume reduction (%)",
                    dimension = 1,
                    col = poscolours)
```

```
mincPlotSliceSeries(mincArray(anatVol),           # the anatomical volume
                    mincArray(vs_W6, "beta-genotypemut"), # pull out one column of the stats
                    anatLow=1000, anatHigh=1500,   # set anatomy thresholds
                    low=-0.1, high=-0.5,             # set stats thresholds
                    begin=72, end=-40,            # remove slices from both sides  
                    # legend="volume reduction (%)",
                    mfrow = c(3,3),
                    dimension = 1,
                    col = poscolours)
```

```
mincPlotSliceSeries(mincArray(anatVol),           # the anatomical volume
                    mincArray(abs(vs_W9*100), "beta-genotypemut"), # pull out one column of the stats
                    anatLow=1000, anatHigh=1500,   # set anatomy thresholds
                    low=abs(-0.1*100), high=abs(-0.5*100),             # set stats thresholds
                    begin=72, end=-40,            # remove slices from both sides  
                    # legend="volume reduction (%)",
                    mfrow = c(3,3),
                    dimension = 1,
                    col = poscolours)
```

```
poscolours = colorRampPalette(c("blue", "red"))(255)

thresholds(qvs_W3) %>% knitr::kable()
```

|  | tvalue-(Intercept) | tvalue-I(time - 3) | tvalue-genotypehet | tvalue-genotypemut | tvalue-I(time - 3):genotypehet | tvalue-I(time - 3):genotypemut |
| --- | --- | --- | --- | --- | --- | --- |
| 0.01 | 3.205248 | 2.812191 | NA | 3.616559 | NA | 2.915275 |
| 0.05 | 2.451407 | 2.119171 | NA | 2.651557 | NA | 2.162094 |
| 0.1 | 2.072632 | 1.773519 | NA | 2.170909 | NA | 1.791640 |
| 0.15 | 1.829017 | 1.550766 | NA | 1.871398 | NA | 1.557834 |
| 0.2 | 1.642190 | 1.381138 | NA | 1.653849 | NA | 1.380142 |

```
mincImage(mincArray(anatVol), slice = 72, axes = F, low = 1000, high = 1400, 
    dimension = 1)
mincImage(mincArray(vs_W3, "beta-genotypemut"), slice = 72, low = -0.1, high = -0.5, 
    add = T, col = poscolours, underTransparent = T, dimension = 1)
mincContour(abs(mincArray(vs_W3, "tvalue-genotypemut")), slice = 72, levels = c(3.62), 
    add = T, dimension = 1, col = "green")
```

```
thresholds(qvs_W6) %>% knitr::kable()
```

|  | tvalue-(Intercept) | tvalue-I(time - 6) | tvalue-genotypehet | tvalue-genotypemut | tvalue-I(time - 6):genotypehet | tvalue-I(time - 6):genotypemut |
| --- | --- | --- | --- | --- | --- | --- |
| 0.01 | 2.863091 | 2.815068 | NA | 2.702513 | NA | 2.920387 |
| 0.05 | 2.151420 | 2.120510 | NA | 2.026456 | NA | 2.164294 |
| 0.1 | 1.800092 | 1.774331 | NA | 1.691809 | NA | 1.792991 |
| 0.15 | 1.574584 | 1.551405 | NA | 1.476999 | NA | 1.558696 |
| 0.2 | 1.402674 | 1.381586 | NA | 1.313472 | NA | 1.380834 |

```
mincImage(mincArray(anatVol), slice = 72, axes = F, low = 1000, high = 1400, 
    dimension = 1)
mincImage(mincArray(vs_W6, "beta-genotypemut"), slice = 72, low = -0.1, high = -0.5, 
    add = T, col = poscolours, underTransparent = T, dimension = 1)
mincContour(abs(mincArray(vs_W6, "tvalue-genotypemut")), slice = 72, levels = c(2.7), 
    add = T, dimension = 1, col = "green")
```

```
thresholds(qvs_W9) %>% knitr::kable()
```

|  | tvalue-(Intercept) | tvalue-I(time - 9) | tvalue-genotypehet | tvalue-genotypemut | tvalue-I(time - 9):genotypehet | tvalue-I(time - 9):genotypemut |
| --- | --- | --- | --- | --- | --- | --- |
| 0.01 | 2.704670 | 2.813686 | NA | 2.667888 | NA | 2.918094 |
| 0.05 | 2.033333 | 2.119867 | NA | 2.007730 | NA | 2.163321 |
| 0.1 | 1.698054 | 1.773939 | NA | 1.677822 | NA | 1.792379 |
| 0.15 | 1.481858 | 1.551073 | NA | 1.465314 | NA | 1.558287 |
| 0.2 | 1.316836 | 1.381364 | NA | 1.302379 | NA | 1.380526 |

```
mincImage(mincArray(anatVol), slice = 72, axes = F, low = 1000, high = 1400, 
    dimension = 1)
mincImage(mincArray(vs_W9, "beta-genotypemut"), slice = 72, low = -0.1, high = -0.5, 
    add = T, col = poscolours, underTransparent = T, dimension = 1)
mincContour(abs(mincArray(vs_W9, "tvalue-genotypemut")), slice = 72, levels = c(2.668), 
    add = T, dimension = 1, col = "green")
```

# Supplementary Figure S1 - Growth Rate Trends

```
mincPlotSliceSeries(mincArray(anatVol),           # the anatomical volume
                    mincArray((vs_W3*100), "beta-I(time - 3):genotypemut"), # pull out one column of the stats
                    anatLow=1000, anatHigh=1500,   # set anatomy thresholds
                    begin=72, end=-40,            # remove slices from both sides  
                    # legend="growth rate differential (%)",
                    low = -1, high = -5,
                    mfrow = c(3,3),
                    dimension = 1,
                    col = poscolours,
                    symmetric = FALSE)
```

# Figure 3 - Genotype-wise subregion volume trends

```
wholebrainvolumetrics <- gf %>% mutate(Thalamus = Thalamus.Volume, Hippocampus = Hippocampus.Volume, 
    `Olfactory Bulb` = OlfactoryBulb.Volume, Midbrain = Midbrain.Volume, Hindbrain = Hindbrain.Volume, 
    Thalamus = Thalamus.Volume, Hypothalamus = Hypothalamus.Volume, Cortex = Cortex.Volume, 
    Cerebellum = CB.Volume) %>% gather(roi, volume, Thalamus, Hippocampus, `Olfactory Bulb`, 
    Hypothalamus, Midbrain, Hindbrain, Cortex, Cerebellum) %>% # mutate(roi=fct_relevel(roi, 'Whole Brain')) %>%
ggplot() + aes(x = time, y = volume, color = genotype) + stat_summary(fun.data = mean_cl_boot, 
    geom = "path") + stat_summary(fun.data = mean_cl_boot) + facet_wrap(~roi, 
    nrow = 2, scales = "free") + scale_x_continuous(breaks = c(3, 6, 9)) + ylab(bquote(bold("Volume" ~ 
    (mm^3)))) + xlab("Age (Weeks)") + scale_color_discrete("Genotype", labels = labeller) + 
    scale_y_continuous(breaks = pretty_breaks(n = 6), labels = number_format(accuracy = 0.1)) + 
    plottheme

wholebrainvolumetrics
```

```
ggsave("wholebrainvolumetrics.png", wholebrainvolumetrics, scale = 3, dpi = "retina", 
    width = 6, height = 3)
```

# Figure 4 - Genotype-wise subregion signal trends

```
wholebrainsignaltrics <- gf %>% mutate(Thalamus = Thalamus.Mean, Hippocampus = Hippocampus.Mean, 
    `Olfactory Bulb` = OlfactoryBulb.Mean, Midbrain = Midbrain.Mean, Hindbrain = Hindbrain.Mean, 
    Thalamus = Thalamus.Mean, Cortex = Cortex.Mean, Hypothalamus = Hypothalamus.Mean, 
    Cerebellum = CB.Mean) %>% gather(roi, Signal, Thalamus, Hippocampus, `Olfactory Bulb`, 
    Midbrain, Hindbrain, Cortex, Hypothalamus, Cerebellum) %>% ggplot() + aes(x = time, 
    y = Signal/WholeBrain.Mean, color = genotype) + stat_summary(fun.data = mean_cl_boot, 
    geom = "line") + stat_summary(fun.data = mean_cl_boot) + facet_wrap(~roi, 
    nrow = 2, scales = "free") + scale_x_continuous(breaks = c(3, 6, 9)) + scale_y_continuous(breaks = pretty_breaks(n = 4), 
    limits = c(0.6, 1.4)) + ylab(bquote(bold("Relative Signal Intensity"))) + 
    xlab("Age (Weeks)") + scale_color_discrete("Genotype", labels = labeller) + 
    plottheme + theme(legend.position = "right")

wholebrainsignaltrics
```

```
## Warning: Removed 2 rows containing non-finite values (stat_summary).

## Warning: Removed 2 rows containing non-finite values (stat_summary).
```

# Quantitative analyses of cerebellar layers

## Volume

```
CB.Cortexlm <- cbdf %>% lmer(CB.Cortex.Volume ~ genotype * timepoint + (1 | 
    mouse), data = .)

CB.Cortex.Volume.Pairwise <- emmeans(CB.Cortexlm, ~genotype * timepoint)
pairs(CB.Cortex.Volume.Pairwise)
```

```
##  contrast        estimate    SE   df t.ratio p.value
##  wt,W3 - het,W3     0.508 0.639 76.9   0.794 0.9967 
##  wt,W3 - mut,W3     3.746 0.764 76.8   4.905 0.0002 
##  wt,W3 - wt,W6     -3.792 0.633 77.7  -5.994 <.0001 
##  wt,W3 - het,W6    -4.196 0.598 75.4  -7.015 <.0001 
##  wt,W3 - mut,W6     0.793 0.657 74.5   1.206 0.9526 
##  wt,W3 - wt,W9     -4.636 0.686 73.3  -6.754 <.0001 
##  wt,W3 - het,W9    -4.997 0.620 77.1  -8.061 <.0001 
##  wt,W3 - mut,W9     4.443 0.681 76.4   6.524 <.0001 
##  het,W3 - mut,W3    3.238 0.677 77.4   4.785 0.0003 
##  het,W3 - wt,W6    -4.300 0.554 75.3  -7.763 <.0001 
##  het,W3 - het,W6   -4.703 0.452 71.1 -10.412 <.0001 
##  het,W3 - mut,W6    0.285 0.554 75.2   0.515 0.9999 
##  het,W3 - wt,W9    -5.143 0.625 77.9  -8.226 <.0001 
##  het,W3 - het,W9   -5.505 0.473 66.0 -11.635 <.0001 
##  het,W3 - mut,W9    3.935 0.582 77.3   6.762 <.0001 
##  mut,W3 - wt,W6    -7.538 0.694 75.8 -10.862 <.0001 
##  mut,W3 - het,W6   -7.942 0.638 76.5 -12.442 <.0001 
##  mut,W3 - mut,W6   -2.953 0.666 76.8  -4.433 0.0010 
##  mut,W3 - wt,W9    -8.382 0.752 78.0 -11.143 <.0001 
##  mut,W3 - het,W9   -8.743 0.659 77.6 -13.271 <.0001 
##  mut,W3 - mut,W9    0.697 0.685 75.2   1.017 0.9831 
##  wt,W6 - het,W6    -0.403 0.506 72.0  -0.797 0.9966 
##  wt,W6 - mut,W6     4.585 0.575 71.3   7.978 <.0001 
##  wt,W6 - wt,W9     -0.844 0.504 34.4  -1.673 0.7582 
##  wt,W6 - het,W9    -1.205 0.532 75.6  -2.267 0.3751 
##  wt,W6 - mut,W9     8.235 0.602 74.8  13.685 <.0001 
##  het,W6 - mut,W6    4.989 0.506 71.8   9.857 <.0001 
##  het,W6 - wt,W9    -0.440 0.583 78.0  -0.755 0.9977 
##  het,W6 - het,W9   -0.802 0.348 32.4  -2.306 0.3676 
##  het,W6 - mut,W9    8.638 0.537 75.8  16.099 <.0001 
##  mut,W6 - wt,W9    -5.429 0.644 77.6  -8.432 <.0001 
##  mut,W6 - het,W9   -5.790 0.532 75.5 -10.890 <.0001 
##  mut,W6 - mut,W9    3.650 0.447 31.3   8.161 <.0001 
##  wt,W9 - het,W9    -0.361 0.606 77.7  -0.597 0.9996 
##  wt,W9 - mut,W9     9.079 0.668 78.0  13.589 <.0001 
##  het,W9 - mut,W9    9.440 0.561 77.5  16.833 <.0001 
## 
## Degrees-of-freedom method: kenward-roger 
## P value adjustment: tukey method for comparing a family of 9 estimates
```

```
summary(CB.Cortexlm)
```

```
## Linear mixed model fit by REML. t-tests use Satterthwaite's method [
## lmerModLmerTest]
## Formula: CB.Cortex.Volume ~ genotype * timepoint + (1 | mouse)
##    Data: .
## 
## REML criterion at convergence: 273
## 
## Scaled residuals: 
##     Min      1Q  Median      3Q     Max 
## -2.5586 -0.4015  0.1013  0.4134  1.9141 
## 
## Random effects:
##  Groups   Name        Variance Std.Dev.
##  mouse    (Intercept) 0.8445   0.9189  
##  Residual             0.8354   0.9140  
## Number of obs: 87, groups:  mouse, 55
## 
## Fixed effects:
##                         Estimate Std. Error      df t value Pr(>|t|)    
## (Intercept)              34.3522     0.5114 76.3386  67.171  < 2e-16 ***
## genotypehet              -0.5075     0.6324 77.0004  -0.803    0.425    
## genotypemut              -3.7460     0.7556 76.8948  -4.958 4.15e-06 ***
## timepointW6               3.7923     0.6224 77.6986   6.093 3.99e-08 ***
## timepointW9               4.6359     0.6746 73.8793   6.872 1.71e-09 ***
## genotypehet:timepointW6   0.9109     0.7639 76.5142   1.192    0.237    
## genotypemut:timepointW6  -0.8392     0.9032 77.3539  -0.929    0.356    
## genotypehet:timepointW9   0.8690     0.8188 72.0021   1.061    0.292    
## genotypemut:timepointW9  -5.3326     0.9530 74.7393  -5.596 3.44e-07 ***
## ---
## Signif. codes:  0 '***' 0.001 '**' 0.01 '*' 0.05 '.' 0.1 ' ' 1
## 
## Correlation of Fixed Effects:
##             (Intr) gntyph gntypm tmpnW6 tmpnW9 gntyph:W6 gntypm:W6
## genotypehet -0.809                                                
## genotypemut -0.677  0.547                                         
## timepointW6 -0.763  0.617  0.516                                  
## timepointW9 -0.686  0.555  0.464  0.705                           
## gntypht:tW6  0.622 -0.756 -0.421 -0.815 -0.575                    
## gntypmt:tW6  0.526 -0.425 -0.777 -0.689 -0.486  0.561             
## gntypht:tW9  0.565 -0.687 -0.382 -0.581 -0.824  0.707     0.400   
## gntypmt:tW9  0.486 -0.393 -0.724 -0.499 -0.708  0.407     0.741   
##             gntyph:W9
## genotypehet          
## genotypemut          
## timepointW6          
## timepointW9          
## gntypht:tW6          
## gntypmt:tW6          
## gntypht:tW9          
## gntypmt:tW9  0.583
```

```
CB.WhiteMatterlm <- cbdf %>% lmer(CB.WhiteMatter.Volume ~ genotype * timepoint + 
    (1 | mouse), data = .)

CB.WhiteMatter.Volume.Pairwise <- emmeans(CB.WhiteMatterlm, ~genotype * timepoint)
pairs(CB.WhiteMatter.Volume.Pairwise)
```

```
##  contrast        estimate     SE   df t.ratio p.value
##  wt,W3 - het,W3    0.0536 0.1290 77.0   0.415 1.0000 
##  wt,W3 - mut,W3    0.5890 0.1541 76.9   3.822 0.0078 
##  wt,W3 - wt,W6    -0.9550 0.1283 77.8  -7.443 <.0001 
##  wt,W3 - het,W6   -1.0363 0.1205 75.8  -8.600 <.0001 
##  wt,W3 - mut,W6   -0.0452 0.1323 75.1  -0.341 1.0000 
##  wt,W3 - wt,W9    -1.2069 0.1402 74.0  -8.611 <.0001 
##  wt,W3 - het,W9   -1.2403 0.1252 77.3  -9.903 <.0001 
##  wt,W3 - mut,W9    0.7651 0.1374 76.7   5.567 <.0001 
##  het,W3 - mut,W3   0.5354 0.1368 77.5   3.914 0.0057 
##  het,W3 - wt,W6   -1.0086 0.1116 75.8  -9.038 <.0001 
##  het,W3 - het,W6  -1.0898 0.0923 72.4 -11.801 <.0001 
##  het,W3 - mut,W6  -0.0987 0.1116 75.7  -0.885 0.9932 
##  het,W3 - wt,W9   -1.2604 0.1270 77.9  -9.923 <.0001 
##  het,W3 - het,W9  -1.2939 0.0971 67.7 -13.319 <.0001 
##  het,W3 - mut,W9   0.7115 0.1177 77.5   6.047 <.0001 
##  mut,W3 - wt,W6   -1.5440 0.1399 76.1 -11.039 <.0001 
##  mut,W3 - het,W6  -1.6253 0.1288 76.7 -12.620 <.0001 
##  mut,W3 - mut,W6  -0.6342 0.1353 77.1  -4.685 0.0004 
##  mut,W3 - wt,W9   -1.7959 0.1525 78.0 -11.779 <.0001 
##  mut,W3 - het,W9  -1.8293 0.1332 77.6 -13.730 <.0001 
##  mut,W3 - mut,W9   0.1761 0.1396 75.8   1.262 0.9392 
##  wt,W6 - het,W6   -0.0812 0.1016 73.0  -0.799 0.9966 
##  wt,W6 - mut,W6    0.9099 0.1154 72.4   7.886 <.0001 
##  wt,W6 - wt,W9    -0.2518 0.1061 35.6  -2.373 0.3290 
##  wt,W6 - het,W9   -0.2853 0.1072 76.2  -2.661 0.1797 
##  wt,W6 - mut,W9    1.7201 0.1212 75.5  14.188 <.0001 
##  het,W6 - mut,W6   0.9911 0.1016 72.8   9.751 <.0001 
##  het,W6 - wt,W9   -0.1706 0.1184 78.0  -1.441 0.8780 
##  het,W6 - het,W9  -0.2041 0.0733 33.4  -2.785 0.1581 
##  het,W6 - mut,W9   1.8013 0.1083 76.3  16.640 <.0001 
##  mut,W6 - wt,W9   -1.1617 0.1304 77.8  -8.912 <.0001 
##  mut,W6 - het,W9  -1.1952 0.1072 76.1 -11.146 <.0001 
##  mut,W6 - mut,W9   0.8102 0.0944 32.1   8.585 <.0001 
##  wt,W9 - het,W9   -0.0335 0.1232 77.7  -0.272 1.0000 
##  wt,W9 - mut,W9    1.9719 0.1356 78.0  14.545 <.0001 
##  het,W9 - mut,W9   2.0054 0.1135 77.7  17.666 <.0001 
## 
## Degrees-of-freedom method: kenward-roger 
## P value adjustment: tukey method for comparing a family of 9 estimates
```

```
summary(CB.WhiteMatterlm)
```

```
## Linear mixed model fit by REML. t-tests use Satterthwaite's method [
## lmerModLmerTest]
## Formula: CB.WhiteMatter.Volume ~ genotype * timepoint + (1 | mouse)
##    Data: .
## 
## REML criterion at convergence: 24
## 
## Scaled residuals: 
##      Min       1Q   Median       3Q      Max 
## -2.31739 -0.42505  0.04597  0.44605  2.10087 
## 
## Random effects:
##  Groups   Name        Variance Std.Dev.
##  mouse    (Intercept) 0.02982  0.1727  
##  Residual             0.03742  0.1935  
## Number of obs: 87, groups:  mouse, 55
## 
## Fixed effects:
##                         Estimate Std. Error       df t value Pr(>|t|)    
## (Intercept)              6.29507    0.10318 76.51333  61.008  < 2e-16 ***
## genotypehet             -0.05358    0.12774 77.11724  -0.419 0.676083    
## genotypemut             -0.58899    0.15259 76.99624  -3.860 0.000234 ***
## timepointW6              0.95503    0.12646 77.80352   7.552 6.98e-11 ***
## timepointW9              1.20687    0.13788 74.37101   8.753 4.68e-13 ***
## genotypehet:timepointW6  0.13481    0.15561 76.82893   0.866 0.389008    
## genotypemut:timepointW6 -0.32088    0.18368 77.53956  -1.747 0.084614 .  
## genotypehet:timepointW9  0.08704    0.16765 72.68871   0.519 0.605196    
## genotypemut:timepointW9 -1.38295    0.19459 75.20539  -7.107 5.78e-10 ***
## ---
## Signif. codes:  0 '***' 0.001 '**' 0.01 '*' 0.05 '.' 0.1 ' ' 1
## 
## Correlation of Fixed Effects:
##             (Intr) gntyph gntypm tmpnW6 tmpnW9 gntyph:W6 gntypm:W6
## genotypehet -0.808                                                
## genotypemut -0.676  0.546                                         
## timepointW6 -0.768  0.621  0.520                                  
## timepointW9 -0.688  0.556  0.466  0.686                           
## gntypht:tW6  0.624 -0.763 -0.422 -0.813 -0.558                    
## gntypmt:tW6  0.529 -0.427 -0.782 -0.688 -0.473  0.559             
## gntypht:tW9  0.566 -0.692 -0.383 -0.564 -0.822  0.689     0.389   
## gntypmt:tW9  0.488 -0.394 -0.727 -0.486 -0.709  0.395     0.723   
##             gntyph:W9
## genotypehet          
## genotypemut          
## timepointW6          
## timepointW9          
## gntypht:tW6          
## gntypmt:tW6          
## gntypht:tW9          
## gntypmt:tW9  0.583
```

```
CB.Nucleilm <- cbdf %>% lmer(CB.Nuclei.Volume ~ genotype * timepoint + (1 | 
    mouse), data = .)

CB.Nuclei.Volume.Pairwise <- emmeans(CB.Nucleilm, ~genotype * timepoint)
pairs(CB.Nuclei.Volume.Pairwise)
```

```
##  contrast        estimate     SE   df t.ratio p.value
##  wt,W3 - het,W3   0.00853 0.0199 77.2   0.428 1.0000 
##  wt,W3 - mut,W3   0.06700 0.0238 77.1   2.813 0.1283 
##  wt,W3 - wt,W6   -0.17560 0.0199 77.8  -8.826 <.0001 
##  wt,W3 - het,W6  -0.17273 0.0186 76.2  -9.287 <.0001 
##  wt,W3 - mut,W6  -0.06195 0.0204 75.5  -3.036 0.0753 
##  wt,W3 - wt,W9   -0.22724 0.0218 74.5 -10.404 <.0001 
##  wt,W3 - het,W9  -0.22522 0.0194 77.4 -11.627 <.0001 
##  wt,W3 - mut,W9   0.03184 0.0212 77.0   1.499 0.8526 
##  het,W3 - mut,W3  0.05847 0.0212 77.6   2.763 0.1435 
##  het,W3 - wt,W6  -0.18413 0.0172 76.2 -10.688 <.0001 
##  het,W3 - het,W6 -0.18126 0.0144 73.1 -12.587 <.0001 
##  het,W3 - mut,W6 -0.07048 0.0172 76.1  -4.091 0.0032 
##  het,W3 - wt,W9  -0.23577 0.0197 77.8 -11.952 <.0001 
##  het,W3 - het,W9 -0.23375 0.0152 68.7 -15.381 <.0001 
##  het,W3 - mut,W9  0.02331 0.0182 77.6   1.280 0.9342 
##  mut,W3 - wt,W6  -0.24260 0.0216 76.4 -11.235 <.0001 
##  mut,W3 - het,W6 -0.23972 0.0199 76.9 -12.049 <.0001 
##  mut,W3 - mut,W6 -0.12895 0.0210 77.3  -6.137 <.0001 
##  mut,W3 - wt,W9  -0.29424 0.0236 78.0 -12.449 <.0001 
##  mut,W3 - het,W9 -0.29222 0.0206 77.7 -14.171 <.0001 
##  mut,W3 - mut,W9 -0.03516 0.0217 76.0  -1.619 0.7912 
##  wt,W6 - het,W6   0.00288 0.0157 73.8   0.184 1.0000 
##  wt,W6 - mut,W6   0.11365 0.0178 73.3   6.399 <.0001 
##  wt,W6 - wt,W9   -0.05164 0.0169 36.7  -3.052 0.0875 
##  wt,W6 - het,W9  -0.04961 0.0166 76.5  -2.995 0.0832 
##  wt,W6 - mut,W9   0.20744 0.0187 75.9  11.084 <.0001 
##  het,W6 - mut,W6  0.11077 0.0157 73.7   7.077 <.0001 
##  het,W6 - wt,W9  -0.05451 0.0184 78.0  -2.968 0.0887 
##  het,W6 - het,W9 -0.05249 0.0117 34.1  -4.486 0.0023 
##  het,W6 - mut,W9  0.20457 0.0167 76.7  12.230 <.0001 
##  mut,W6 - wt,W9  -0.16529 0.0202 77.8  -8.185 <.0001 
##  mut,W6 - het,W9 -0.16327 0.0166 76.5  -9.857 <.0001 
##  mut,W6 - mut,W9  0.09379 0.0151 32.7   6.218 <.0001 
##  wt,W9 - het,W9   0.00202 0.0191 77.7   0.106 1.0000 
##  wt,W9 - mut,W9   0.25908 0.0210 78.0  12.315 <.0001 
##  het,W9 - mut,W9  0.25706 0.0176 77.8  14.620 <.0001 
## 
## Degrees-of-freedom method: kenward-roger 
## P value adjustment: tukey method for comparing a family of 9 estimates
```

```
summary(CB.Nucleilm)
```

```
## Linear mixed model fit by REML. t-tests use Satterthwaite's method [
## lmerModLmerTest]
## Formula: CB.Nuclei.Volume ~ genotype * timepoint + (1 | mouse)
##    Data: .
## 
## REML criterion at convergence: -266.8
## 
## Scaled residuals: 
##      Min       1Q   Median       3Q      Max 
## -2.48230 -0.50282  0.04562  0.52595  2.00060 
## 
## Random effects:
##  Groups   Name        Variance  Std.Dev.
##  mouse    (Intercept) 0.0006264 0.02503 
##  Residual             0.0009608 0.03100 
## Number of obs: 87, groups:  mouse, 55
## 
## Fixed effects:
##                          Estimate Std. Error        df t value Pr(>|t|)
## (Intercept)              0.842992   0.015943 76.892508  52.877  < 2e-16
## genotypehet             -0.008530   0.019754 77.352089  -0.432  0.66706
## genotypemut             -0.066997   0.023591 77.248662  -2.840  0.00576
## timepointW6              0.175604   0.019633 77.855795   8.944 1.40e-13
## timepointW9              0.227240   0.021501 75.124883  10.569  < 2e-16
## genotypehet:timepointW6  0.005652   0.024205 77.108089   0.234  0.81598
## genotypemut:timepointW6 -0.046655   0.028537 77.661332  -1.635  0.10612
## genotypehet:timepointW9  0.006509   0.026178 73.778156   0.249  0.80432
## genotypemut:timepointW9 -0.192082   0.030323 75.813628  -6.335 1.54e-08
##                            
## (Intercept)             ***
## genotypehet                
## genotypemut             ** 
## timepointW6             ***
## timepointW9             ***
## genotypehet:timepointW6    
## genotypemut:timepointW6    
## genotypehet:timepointW9    
## genotypemut:timepointW9 ***
## ---
## Signif. codes:  0 '***' 0.001 '**' 0.01 '*' 0.05 '.' 0.1 ' ' 1
## 
## Correlation of Fixed Effects:
##             (Intr) gntyph gntypm tmpnW6 tmpnW9 gntyph:W6 gntypm:W6
## genotypehet -0.807                                                
## genotypemut -0.676  0.545                                         
## timepointW6 -0.772  0.623  0.522                                  
## timepointW9 -0.690  0.557  0.467  0.671                           
## gntypht:tW6  0.626 -0.767 -0.423 -0.811 -0.544                    
## gntypmt:tW6  0.531 -0.429 -0.786 -0.688 -0.462  0.558             
## gntypht:tW9  0.567 -0.695 -0.383 -0.551 -0.821  0.675     0.379   
## gntypmt:tW9  0.490 -0.395 -0.730 -0.476 -0.709  0.386     0.709   
##             gntyph:W9
## genotypehet          
## genotypemut          
## timepointW6          
## timepointW9          
## gntypht:tW6          
## gntypmt:tW6          
## gntypht:tW9          
## gntypmt:tW9  0.582
```

```
CB.Hemispherelm <- cbdf %>% lmer(CB.Hemisphere.Volume ~ genotype * timepoint + 
    (1 | mouse), data = .)

CB.Hemisphere.Volume.Pairwise <- emmeans(CB.Hemispherelm, ~genotype * timepoint)
pairs(CB.Hemisphere.Volume.Pairwise)
```

```
##  contrast        estimate    SE   df t.ratio p.value
##  wt,W3 - het,W3     0.365 0.383 76.8   0.952 0.9890 
##  wt,W3 - mut,W3     2.214 0.458 76.7   4.830 0.0002 
##  wt,W3 - wt,W6     -2.393 0.378 77.4  -6.333 <.0001 
##  wt,W3 - het,W6    -2.748 0.360 75.2  -7.642 <.0001 
##  wt,W3 - mut,W6     0.489 0.395 74.2   1.237 0.9455 
##  wt,W3 - wt,W9     -2.922 0.407 72.6  -7.172 <.0001 
##  wt,W3 - het,W9    -3.231 0.372 77.0  -8.691 <.0001 
##  wt,W3 - mut,W9     2.504 0.409 76.2   6.126 <.0001 
##  het,W3 - mut,W3    1.849 0.406 77.4   4.557 0.0006 
##  het,W3 - wt,W6    -2.758 0.333 75.0  -8.284 <.0001 
##  het,W3 - het,W6   -3.113 0.268 69.7 -11.625 <.0001 
##  het,W3 - mut,W6    0.124 0.333 74.8   0.373 1.0000 
##  het,W3 - wt,W9    -3.287 0.373 77.9  -8.811 <.0001 
##  het,W3 - het,W9   -3.596 0.279 64.4 -12.869 <.0001 
##  het,W3 - mut,W9    2.139 0.349 77.1   6.135 <.0001 
##  mut,W3 - wt,W6    -4.607 0.417 75.6 -11.047 <.0001 
##  mut,W3 - het,W6   -4.962 0.383 76.4 -12.948 <.0001 
##  mut,W3 - mut,W6   -1.725 0.397 76.3  -4.344 0.0013 
##  mut,W3 - wt,W9    -5.136 0.450 78.0 -11.420 <.0001 
##  mut,W3 - het,W9   -5.445 0.395 77.5 -13.796 <.0001 
##  mut,W3 - mut,W9    0.290 0.408 74.5   0.713 0.9985 
##  wt,W6 - het,W6    -0.355 0.305 71.3  -1.163 0.9616 
##  wt,W6 - mut,W6     2.882 0.347 70.5   8.313 <.0001 
##  wt,W6 - wt,W9     -0.529 0.292 33.5  -1.815 0.6725 
##  wt,W6 - het,W9    -0.838 0.319 75.2  -2.624 0.1944 
##  wt,W6 - mut,W9     4.897 0.362 74.3  13.535 <.0001 
##  het,W6 - mut,W6    3.237 0.305 71.1  10.608 <.0001 
##  het,W6 - wt,W9    -0.175 0.348 78.0  -0.501 0.9999 
##  het,W6 - het,W9   -0.483 0.201 31.7  -2.407 0.3146 
##  het,W6 - mut,W9    5.252 0.322 75.4  16.300 <.0001 
##  mut,W6 - wt,W9    -3.411 0.385 77.5  -8.851 <.0001 
##  mut,W6 - het,W9   -3.720 0.319 75.0 -11.645 <.0001 
##  mut,W6 - mut,W9    2.015 0.258 30.7   7.811 <.0001 
##  wt,W9 - het,W9    -0.309 0.361 77.8  -0.854 0.9946 
##  wt,W9 - mut,W9     5.427 0.399 78.0  13.597 <.0001 
##  het,W9 - mut,W9    5.735 0.336 77.3  17.079 <.0001 
## 
## Degrees-of-freedom method: kenward-roger 
## P value adjustment: tukey method for comparing a family of 9 estimates
```

```
summary(CB.Hemispherelm)
```

```
## Linear mixed model fit by REML. t-tests use Satterthwaite's method [
## lmerModLmerTest]
## Formula: CB.Hemisphere.Volume ~ genotype * timepoint + (1 | mouse)
##    Data: .
## 
## REML criterion at convergence: 192.6
## 
## Scaled residuals: 
##      Min       1Q   Median       3Q      Max 
## -2.43851 -0.42369  0.03292  0.38879  1.98541 
## 
## Random effects:
##  Groups   Name        Variance Std.Dev.
##  mouse    (Intercept) 0.3386   0.5819  
##  Residual             0.2768   0.5261  
## Number of obs: 87, groups:  mouse, 55
## 
## Fixed effects:
##                         Estimate Std. Error      df t value Pr(>|t|)    
## (Intercept)              19.3562     0.3070 76.1762  63.044  < 2e-16 ***
## genotypehet              -0.3650     0.3793 76.8918  -0.962    0.339    
## genotypemut              -2.2139     0.4532 76.8094  -4.885 5.52e-06 ***
## timepointW6               2.3930     0.3712 77.4929   6.447 8.89e-09 ***
## timepointW9               2.9224     0.4002 73.1077   7.303 2.82e-10 ***
## genotypehet:timepointW6   0.7197     0.4545 75.9882   1.584    0.117    
## genotypemut:timepointW6  -0.6681     0.5381 77.0239  -1.242    0.218    
## genotypehet:timepointW9   0.6735     0.4849 70.9548   1.389    0.169    
## genotypemut:timepointW9  -3.2129     0.5657 74.0049  -5.679 2.51e-07 ***
## ---
## Signif. codes:  0 '***' 0.001 '**' 0.01 '*' 0.05 '.' 0.1 ' ' 1
## 
## Correlation of Fixed Effects:
##             (Intr) gntyph gntypm tmpnW6 tmpnW9 gntyph:W6 gntypm:W6
## genotypehet -0.810                                                
## genotypemut -0.677  0.548                                         
## timepointW6 -0.758  0.613  0.513                                  
## timepointW9 -0.683  0.553  0.463  0.721                           
## gntypht:tW6  0.619 -0.750 -0.419 -0.817 -0.589                    
## gntypmt:tW6  0.523 -0.423 -0.771 -0.690 -0.497  0.563             
## gntypht:tW9  0.564 -0.684 -0.382 -0.595 -0.825  0.722     0.410   
## gntypmt:tW9  0.483 -0.391 -0.720 -0.510 -0.707  0.416     0.755   
##             gntyph:W9
## genotypehet          
## genotypemut          
## timepointW6          
## timepointW9          
## gntypht:tW6          
## gntypmt:tW6          
## gntypht:tW9          
## gntypmt:tW9  0.584
```

```
CB.Vermislm <- cbdf %>% lmer(CB.Vermis.Volume ~ genotype * timepoint + (1 | 
    mouse), data = .)

CB.Vermis.Volume.Pairwise <- emmeans(CB.Vermislm, ~genotype * timepoint)
pairs(CB.Vermis.Volume.Pairwise)
```

```
##  contrast        estimate    SE   df t.ratio p.value
##  wt,W3 - het,W3    0.1436 0.282 77.1   0.509 0.9999 
##  wt,W3 - mut,W3    1.5012 0.337 77.0   4.454 0.0009 
##  wt,W3 - wt,W6    -1.4007 0.281 77.8  -4.979 0.0001 
##  wt,W3 - het,W6   -1.4513 0.263 76.0  -5.511 <.0001 
##  wt,W3 - mut,W6    0.3210 0.289 75.4   1.111 0.9709 
##  wt,W3 - wt,W9    -1.7255 0.308 74.3  -5.597 <.0001 
##  wt,W3 - het,W9   -1.7696 0.274 77.4  -6.456 <.0001 
##  wt,W3 - mut,W9    1.9519 0.301 76.9   6.493 <.0001 
##  het,W3 - mut,W3   1.3576 0.299 77.5   4.535 0.0007 
##  het,W3 - wt,W6   -1.5442 0.244 76.0  -6.331 <.0001 
##  het,W3 - het,W6  -1.5948 0.203 72.9  -7.847 <.0001 
##  het,W3 - mut,W6   0.1774 0.244 76.0   0.727 0.9982 
##  het,W3 - wt,W9   -1.8691 0.279 77.8  -6.705 <.0001 
##  het,W3 - het,W9  -1.9131 0.214 68.4  -8.929 <.0001 
##  het,W3 - mut,W9   1.8083 0.258 77.6   7.020 <.0001 
##  mut,W3 - wt,W6   -2.9019 0.306 76.3  -9.492 <.0001 
##  mut,W3 - het,W6  -2.9525 0.282 76.8 -10.484 <.0001 
##  mut,W3 - mut,W6  -1.1802 0.297 77.2  -3.974 0.0047 
##  mut,W3 - wt,W9   -3.2267 0.334 78.0  -9.656 <.0001 
##  mut,W3 - het,W9  -3.2708 0.292 77.7 -11.213 <.0001 
##  mut,W3 - mut,W9   0.4507 0.307 76.0   1.470 0.8658 
##  wt,W6 - het,W6   -0.0506 0.222 73.5  -0.228 1.0000 
##  wt,W6 - mut,W6    1.7217 0.252 73.0   6.840 <.0001 
##  wt,W6 - wt,W9    -0.3249 0.237 36.3  -1.370 0.9016 
##  wt,W6 - het,W9   -0.3689 0.234 76.4  -1.573 0.8160 
##  wt,W6 - mut,W9    3.3526 0.265 75.8  12.653 <.0001 
##  het,W6 - mut,W6   1.7722 0.222 73.4   7.991 <.0001 
##  het,W6 - wt,W9   -0.2743 0.260 78.0  -1.056 0.9786 
##  het,W6 - het,W9  -0.3183 0.164 33.9  -1.942 0.5911 
##  het,W6 - mut,W9   3.4032 0.237 76.6  14.375 <.0001 
##  mut,W6 - wt,W9   -2.0465 0.286 77.8  -7.166 <.0001 
##  mut,W6 - het,W9  -2.0905 0.234 76.3  -8.916 <.0001 
##  mut,W6 - mut,W9   1.6309 0.211 32.5   7.722 <.0001 
##  wt,W9 - het,W9   -0.0440 0.271 77.7  -0.163 1.0000 
##  wt,W9 - mut,W9    3.6774 0.297 78.0  12.367 <.0001 
##  het,W9 - mut,W9   3.7215 0.249 77.8  14.966 <.0001 
## 
## Degrees-of-freedom method: kenward-roger 
## P value adjustment: tukey method for comparing a family of 9 estimates
```

```
summary(CB.Vermislm)
```

```
## Linear mixed model fit by REML. t-tests use Satterthwaite's method [
## lmerModLmerTest]
## Formula: CB.Vermis.Volume ~ genotype * timepoint + (1 | mouse)
##    Data: .
## 
## REML criterion at convergence: 146.5
## 
## Scaled residuals: 
##     Min      1Q  Median      3Q     Max 
## -2.4337 -0.4032  0.1662  0.4928  1.8416 
## 
## Random effects:
##  Groups   Name        Variance Std.Dev.
##  mouse    (Intercept) 0.1311   0.3620  
##  Residual             0.1880   0.4336  
## Number of obs: 87, groups:  mouse, 55
## 
## Fixed effects:
##                         Estimate Std. Error      df t value Pr(>|t|)    
## (Intercept)              14.9945     0.2257 76.7465  66.450  < 2e-16 ***
## genotypehet              -0.1436     0.2795 77.2627  -0.514    0.609    
## genotypemut              -1.5012     0.3338 77.1504  -4.497 2.40e-05 ***
## timepointW6               1.4007     0.2775 77.8404   5.048 2.86e-06 ***
## timepointW9               1.7255     0.3034 74.8358   5.687 2.37e-07 ***
## genotypehet:timepointW6   0.1942     0.3419 77.0112   0.568    0.572    
## genotypemut:timepointW6  -0.2204     0.4032 77.6223  -0.547    0.586    
## genotypehet:timepointW9   0.1876     0.3693 73.3585   0.508    0.613    
## genotypemut:timepointW9  -2.1762     0.4280 75.5863  -5.084 2.60e-06 ***
## ---
## Signif. codes:  0 '***' 0.001 '**' 0.01 '*' 0.05 '.' 0.1 ' ' 1
## 
## Correlation of Fixed Effects:
##             (Intr) gntyph gntypm tmpnW6 tmpnW9 gntyph:W6 gntypm:W6
## genotypehet -0.807                                                
## genotypemut -0.676  0.546                                         
## timepointW6 -0.771  0.622  0.521                                  
## timepointW9 -0.690  0.557  0.466  0.676                           
## gntypht:tW6  0.626 -0.766 -0.423 -0.812 -0.549                    
## gntypmt:tW6  0.531 -0.428 -0.785 -0.688 -0.465  0.558             
## gntypht:tW9  0.567 -0.694 -0.383 -0.556 -0.822  0.679     0.382   
## gntypmt:tW9  0.489 -0.395 -0.729 -0.479 -0.709  0.389     0.714   
##             gntyph:W9
## genotypehet          
## genotypemut          
## timepointW6          
## timepointW9          
## gntypht:tW6          
## gntypmt:tW6          
## gntypht:tW9          
## gntypmt:tW9  0.583
```

## Signal

```
CB.Cortexlm.Signal <- cbdf %>% lmer(CB.Cortex.Mean/WholeBrain.Mean ~ genotype * 
    timepoint + (1 | mouse), data = .)

CB.Cortex.SignalPairwise <- emmeans(CB.Cortexlm.Signal, ~genotype * timepoint)
pairs(CB.Cortex.SignalPairwise)
```

```
##  contrast        estimate     SE   df t.ratio p.value
##  wt,W3 - het,W3   0.01759 0.0223 78.0  0.787  0.9969 
##  wt,W3 - mut,W3  -0.04482 0.0267 78.0 -1.682  0.7557 
##  wt,W3 - wt,W6    0.01137 0.0226 76.9  0.503  0.9999 
##  wt,W3 - het,W6   0.04805 0.0207 78.0  2.320  0.3432 
##  wt,W3 - mut,W6   0.04201 0.0227 78.0  1.854  0.6467 
##  wt,W3 - wt,W9    0.01290 0.0254 75.5  0.509  0.9999 
##  wt,W3 - het,W9   0.04671 0.0217 78.0  2.149  0.4490 
##  wt,W3 - mut,W9  -0.10239 0.0238 78.0 -4.309  0.0015 
##  het,W3 - mut,W3 -0.06241 0.0238 78.0 -2.625  0.1933 
##  het,W3 - wt,W6  -0.00622 0.0192 78.0 -0.324  1.0000 
##  het,W3 - het,W6  0.03046 0.0168 74.2  1.816  0.6718 
##  het,W3 - mut,W6  0.02442 0.0192 78.0  1.272  0.9364 
##  het,W3 - wt,W9  -0.00469 0.0225 78.0 -0.209  1.0000 
##  het,W3 - het,W9  0.02912 0.0180 72.3  1.621  0.7904 
##  het,W3 - mut,W9 -0.11998 0.0205 78.0 -5.858  <.0001 
##  mut,W3 - wt,W6   0.05619 0.0241 78.0  2.334  0.3352 
##  mut,W3 - het,W6  0.09287 0.0222 78.0  4.176  0.0024 
##  mut,W3 - mut,W6  0.08684 0.0240 76.6  3.616  0.0148 
##  mut,W3 - wt,W9   0.05772 0.0268 78.0  2.158  0.4431 
##  mut,W3 - het,W9  0.09154 0.0232 78.0  3.945  0.0052 
##  mut,W3 - mut,W9 -0.05757 0.0250 76.0 -2.302  0.3542 
##  wt,W6 - het,W6   0.03668 0.0173 77.9  2.125  0.4645 
##  wt,W6 - mut,W6   0.03065 0.0196 77.9  1.566  0.8197 
##  wt,W6 - wt,W9    0.00153 0.0223 44.9  0.069  1.0000 
##  wt,W6 - het,W9   0.03534 0.0185 78.0  1.912  0.6079 
##  wt,W6 - mut,W9  -0.11376 0.0208 78.0 -5.462  <.0001 
##  het,W6 - mut,W6 -0.00604 0.0173 77.9 -0.350  1.0000 
##  het,W6 - wt,W9  -0.03515 0.0208 78.0 -1.687  0.7526 
##  het,W6 - het,W9 -0.00134 0.0156 40.8 -0.086  1.0000 
##  het,W6 - mut,W9 -0.15044 0.0187 78.0 -8.054  <.0001 
##  mut,W6 - wt,W9  -0.02911 0.0228 78.0 -1.278  0.9348 
##  mut,W6 - het,W9  0.00470 0.0185 78.0  0.254  1.0000 
##  mut,W6 - mut,W9 -0.14441 0.0203 38.6 -7.117  <.0001 
##  wt,W9 - het,W9   0.03381 0.0219 78.0  1.547  0.8298 
##  wt,W9 - mut,W9  -0.11529 0.0239 78.0 -4.829  0.0002 
##  het,W9 - mut,W9 -0.14911 0.0198 78.0 -7.524  <.0001 
## 
## Degrees-of-freedom method: kenward-roger 
## P value adjustment: tukey method for comparing a family of 9 estimates
```

```
summary(CB.Cortexlm.Signal)
```

```
## Linear mixed model fit by REML. t-tests use Satterthwaite's method [
## lmerModLmerTest]
## Formula: CB.Cortex.Mean/WholeBrain.Mean ~ genotype * timepoint + (1 |  
##     mouse)
##    Data: .
## 
## REML criterion at convergence: -247.6
## 
## Scaled residuals: 
##     Min      1Q  Median      3Q     Max 
## -2.7395 -0.6107  0.1454  0.5436  2.7299 
## 
## Random effects:
##  Groups   Name        Variance  Std.Dev.
##  mouse    (Intercept) 9.837e-05 0.009918
##  Residual             1.806e-03 0.042494
## Number of obs: 87, groups:  mouse, 55
## 
## Fixed effects:
##                         Estimate Std. Error       df t value Pr(>|t|)    
## (Intercept)              1.00637    0.01781 77.98251  56.513   <2e-16 ***
## genotypehet             -0.01759    0.02214 77.99297  -0.795   0.4292    
## genotypemut              0.04482    0.02641 77.99018   1.697   0.0937 .  
## timepointW6             -0.01137    0.02245 76.97482  -0.506   0.6141    
## timepointW9             -0.01290    0.02507 75.62945  -0.515   0.6084    
## genotypehet:timepointW6 -0.01909    0.02793 76.24739  -0.684   0.4963    
## genotypemut:timepointW6 -0.07547    0.03273 76.83465  -2.306   0.0238 *  
## genotypehet:timepointW9 -0.01622    0.03072 74.73959  -0.528   0.5991    
## genotypemut:timepointW9  0.07047    0.03524 75.87488   2.000   0.0491 *  
## ---
## Signif. codes:  0 '***' 0.001 '**' 0.01 '*' 0.05 '.' 0.1 ' ' 1
## 
## Correlation of Fixed Effects:
##             (Intr) gntyph gntypm tmpnW6 tmpnW9 gntyph:W6 gntypm:W6
## genotypehet -0.804                                                
## genotypemut -0.674  0.542                                         
## timepointW6 -0.789  0.635  0.532                                  
## timepointW9 -0.704  0.567  0.475  0.573                           
## gntypht:tW6  0.634 -0.788 -0.428 -0.804 -0.461                    
## gntypmt:tW6  0.541 -0.435 -0.803 -0.686 -0.393  0.552             
## gntypht:tW9  0.575 -0.714 -0.388 -0.468 -0.816  0.580     0.321   
## gntypmt:tW9  0.501 -0.403 -0.744 -0.408 -0.711  0.328     0.614   
##             gntyph:W9
## genotypehet          
## genotypemut          
## timepointW6          
## timepointW9          
## gntypht:tW6          
## gntypmt:tW6          
## gntypht:tW9          
## gntypmt:tW9  0.581
```

```
CB.WhiteMatterlm.Signal <- cbdf %>% lmer(CB.WhiteMatter.Mean/WholeBrain.Mean ~ 
    genotype * timepoint + (1 | mouse), data = .)
```

```
## boundary (singular) fit: see ?isSingular
```

```
CB.WhiteMatter.SignalPairwise <- emmeans(CB.WhiteMatterlm.Signal, ~genotype * 
    timepoint)
pairs(CB.WhiteMatter.SignalPairwise)
```

```
##  contrast        estimate     SE   df t.ratio p.value
##  wt,W3 - het,W3   0.02703 0.0249 78.0   1.086 0.9747 
##  wt,W3 - mut,W3  -0.07988 0.0297 78.0  -2.691 0.1683 
##  wt,W3 - wt,W6    0.13910 0.0252 76.5   5.513 <.0001 
##  wt,W3 - het,W6   0.17112 0.0231 78.0   7.422 <.0001 
##  wt,W3 - mut,W6   0.05156 0.0252 78.0   2.044 0.5186 
##  wt,W3 - wt,W9    0.14130 0.0283 75.5   4.988 0.0001 
##  wt,W3 - het,W9   0.16561 0.0242 78.0   6.841 <.0001 
##  wt,W3 - mut,W9  -0.10261 0.0265 78.0  -3.879 0.0064 
##  het,W3 - mut,W3 -0.10690 0.0265 78.0  -4.036 0.0038 
##  het,W3 - wt,W6   0.11207 0.0214 78.0   5.243 <.0001 
##  het,W3 - het,W6  0.14410 0.0188 74.1   7.682 <.0001 
##  het,W3 - mut,W6  0.02453 0.0214 78.0   1.148 0.9646 
##  het,W3 - wt,W9   0.11428 0.0250 78.0   4.568 0.0006 
##  het,W3 - het,W9  0.13859 0.0201 72.5   6.890 <.0001 
##  het,W3 - mut,W9 -0.12963 0.0228 78.0  -5.685 <.0001 
##  mut,W3 - wt,W6   0.21898 0.0268 78.0   8.170 <.0001 
##  mut,W3 - het,W6  0.25100 0.0248 78.0  10.134 <.0001 
##  mut,W3 - mut,W6  0.13144 0.0268 76.3   4.904 0.0002 
##  mut,W3 - wt,W9   0.22118 0.0298 78.0   7.424 <.0001 
##  mut,W3 - het,W9  0.24549 0.0258 78.0   9.499 <.0001 
##  mut,W3 - mut,W9 -0.02273 0.0279 75.8  -0.814 0.9961 
##  wt,W6 - het,W6   0.03202 0.0192 78.0   1.668 0.7638 
##  wt,W6 - mut,W6  -0.08754 0.0218 78.0  -4.023 0.0040 
##  wt,W6 - wt,W9    0.00220 0.0253 46.3   0.087 1.0000 
##  wt,W6 - het,W9   0.02652 0.0206 78.0   1.289 0.9316 
##  wt,W6 - mut,W9  -0.24170 0.0232 78.0 -10.432 <.0001 
##  het,W6 - mut,W6 -0.11956 0.0192 78.0  -6.227 <.0001 
##  het,W6 - wt,W9  -0.02982 0.0232 78.0  -1.286 0.9325 
##  het,W6 - het,W9 -0.00551 0.0178 42.0  -0.310 1.0000 
##  het,W6 - mut,W9 -0.27373 0.0208 78.0 -13.172 <.0001 
##  mut,W6 - wt,W9   0.08974 0.0254 78.0   3.540 0.0185 
##  mut,W6 - het,W9  0.11405 0.0206 78.0   5.545 <.0001 
##  mut,W6 - mut,W9 -0.15416 0.0231 39.8  -6.672 <.0001 
##  wt,W9 - het,W9   0.02431 0.0243 78.0   0.999 0.9849 
##  wt,W9 - mut,W9  -0.24391 0.0266 78.0  -9.180 <.0001 
##  het,W9 - mut,W9 -0.26822 0.0221 78.0 -12.163 <.0001 
## 
## Degrees-of-freedom method: kenward-roger 
## P value adjustment: tukey method for comparing a family of 9 estimates
```

```
summary(CB.WhiteMatterlm.Signal)
```

```
## Linear mixed model fit by REML. t-tests use Satterthwaite's method [
## lmerModLmerTest]
## Formula: CB.WhiteMatter.Mean/WholeBrain.Mean ~ genotype * timepoint +  
##     (1 | mouse)
##    Data: .
## 
## REML criterion at convergence: -230.9
## 
## Scaled residuals: 
##      Min       1Q   Median       3Q      Max 
## -2.08728 -0.59133 -0.07682  0.64184  2.60036 
## 
## Random effects:
##  Groups   Name        Variance Std.Dev.
##  mouse    (Intercept) 0.000000 0.00000 
##  Residual             0.002356 0.04854 
## Number of obs: 87, groups:  mouse, 55
## 
## Fixed effects:
##                          Estimate Std. Error        df t value Pr(>|t|)
## (Intercept)              1.099533   0.019815 78.000000  55.489  < 2e-16
## genotypehet             -0.027025   0.024634 78.000000  -1.097  0.27599
## genotypemut              0.079879   0.029391 78.000000   2.718  0.00809
## timepointW6             -0.139097   0.025065 78.000000  -5.549 3.80e-07
## timepointW9             -0.141301   0.028023 78.000000  -5.042 2.92e-06
## genotypehet:timepointW6 -0.005000   0.031198 78.000000  -0.160  0.87309
## genotypemut:timepointW6  0.007659   0.036538 78.000000   0.210  0.83450
## genotypehet:timepointW9  0.002713   0.034361 78.000000   0.079  0.93727
## genotypemut:timepointW9  0.164028   0.039382 78.000000   4.165 7.97e-05
##                            
## (Intercept)             ***
## genotypehet                
## genotypemut             ** 
## timepointW6             ***
## timepointW9             ***
## genotypehet:timepointW6    
## genotypemut:timepointW6    
## genotypehet:timepointW9    
## genotypemut:timepointW9 ***
## ---
## Signif. codes:  0 '***' 0.001 '**' 0.01 '*' 0.05 '.' 0.1 ' ' 1
## 
## Correlation of Fixed Effects:
##             (Intr) gntyph gntypm tmpnW6 tmpnW9 gntyph:W6 gntypm:W6
## genotypehet -0.804                                                
## genotypemut -0.674  0.542                                         
## timepointW6 -0.791  0.636  0.533                                  
## timepointW9 -0.707  0.569  0.477  0.559                           
## gntypht:tW6  0.635 -0.790 -0.428 -0.803 -0.449                    
## gntypmt:tW6  0.542 -0.436 -0.804 -0.686 -0.383  0.551             
## gntypht:tW9  0.577 -0.717 -0.389 -0.456 -0.816  0.566     0.313   
## gntypmt:tW9  0.503 -0.405 -0.746 -0.398 -0.712  0.320     0.600   
##             gntyph:W9
## genotypehet          
## genotypemut          
## timepointW6          
## timepointW9          
## gntypht:tW6          
## gntypmt:tW6          
## gntypht:tW9          
## gntypmt:tW9  0.580   
## convergence code: 0
## boundary (singular) fit: see ?isSingular
```

```
CB.Nucleilm.Signal <- cbdf %>% lmer(CB.Nuclei.Mean/WholeBrain.Mean ~ genotype * 
    timepoint + (1 | mouse), data = .)
```

```
## boundary (singular) fit: see ?isSingular
```

```
CB.Nuclei.SignalPairwise <- emmeans(CB.Nucleilm.Signal, ~genotype * timepoint)
pairs(CB.Nuclei.SignalPairwise)
```

```
##  contrast        estimate     SE   df t.ratio p.value
##  wt,W3 - het,W3   0.03195 0.0287 78.0  1.115  0.9703 
##  wt,W3 - mut,W3  -0.05818 0.0342 78.0 -1.703  0.7431 
##  wt,W3 - wt,W6    0.21016 0.0290 76.5  7.236  <.0001 
##  wt,W3 - het,W6   0.23951 0.0265 78.0  9.024  <.0001 
##  wt,W3 - mut,W6   0.13653 0.0290 78.0  4.701  0.0004 
##  wt,W3 - wt,W9    0.26073 0.0326 75.5  7.997  <.0001 
##  wt,W3 - het,W9   0.27831 0.0279 78.0  9.988  <.0001 
##  wt,W3 - mut,W9   0.04134 0.0304 78.0  1.358  0.9099 
##  het,W3 - mut,W3 -0.09013 0.0305 78.0 -2.956  0.0914 
##  het,W3 - wt,W6   0.17821 0.0246 78.0  7.243  <.0001 
##  het,W3 - het,W6  0.20756 0.0216 74.1  9.612  <.0001 
##  het,W3 - mut,W6  0.10458 0.0246 78.0  4.251  0.0018 
##  het,W3 - wt,W9   0.22878 0.0288 78.0  7.945  <.0001 
##  het,W3 - het,W9  0.24637 0.0232 72.5 10.641  <.0001 
##  het,W3 - mut,W9  0.00939 0.0262 78.0  0.358  1.0000 
##  mut,W3 - wt,W6   0.26834 0.0309 78.0  8.697  <.0001 
##  mut,W3 - het,W6  0.29769 0.0285 78.0 10.441  <.0001 
##  mut,W3 - mut,W6  0.19471 0.0309 76.3  6.311  <.0001 
##  mut,W3 - wt,W9   0.31891 0.0343 78.0  9.300  <.0001 
##  mut,W3 - het,W9  0.33649 0.0297 78.0 11.311  <.0001 
##  mut,W3 - mut,W9  0.09952 0.0322 75.8  3.095  0.0647 
##  wt,W6 - het,W6   0.02935 0.0221 78.0  1.328  0.9198 
##  wt,W6 - mut,W6  -0.07363 0.0250 78.0 -2.939  0.0951 
##  wt,W6 - wt,W9    0.05057 0.0291 46.3  1.739  0.7201 
##  wt,W6 - het,W9   0.06816 0.0237 78.0  2.879  0.1100 
##  wt,W6 - mut,W9  -0.16882 0.0267 78.0 -6.330  <.0001 
##  het,W6 - mut,W6 -0.10298 0.0221 78.0 -4.659  0.0004 
##  het,W6 - wt,W9   0.02122 0.0267 78.0  0.795  0.9967 
##  het,W6 - het,W9  0.03881 0.0204 42.0  1.898  0.6186 
##  het,W6 - mut,W9 -0.19817 0.0239 78.0 -8.284  <.0001 
##  mut,W6 - wt,W9   0.12420 0.0292 78.0  4.256  0.0018 
##  mut,W6 - het,W9  0.14178 0.0237 78.0  5.989  <.0001 
##  mut,W6 - mut,W9 -0.09519 0.0266 39.8 -3.579  0.0233 
##  wt,W9 - het,W9   0.01758 0.0280 78.0  0.628  0.9994 
##  wt,W9 - mut,W9  -0.21939 0.0306 78.0 -7.174  <.0001 
##  het,W9 - mut,W9 -0.23698 0.0254 78.0 -9.336  <.0001 
## 
## Degrees-of-freedom method: kenward-roger 
## P value adjustment: tukey method for comparing a family of 9 estimates
```

```
summary(CB.Nucleilm.Signal)
```

```
## Linear mixed model fit by REML. t-tests use Satterthwaite's method [
## lmerModLmerTest]
## Formula: CB.Nuclei.Mean/WholeBrain.Mean ~ genotype * timepoint + (1 |  
##     mouse)
##    Data: .
## 
## REML criterion at convergence: -208.9
## 
## Scaled residuals: 
##      Min       1Q   Median       3Q      Max 
## -2.52915 -0.59720  0.05802  0.68232  2.80552 
## 
## Random effects:
##  Groups   Name        Variance Std.Dev.
##  mouse    (Intercept) 0.000000 0.00000 
##  Residual             0.003122 0.05587 
## Number of obs: 87, groups:  mouse, 55
## 
## Fixed effects:
##                          Estimate Std. Error        df t value Pr(>|t|)
## (Intercept)              1.208803   0.022809 78.000000  52.996  < 2e-16
## genotypehet             -0.031945   0.028355 78.000000  -1.127 0.263369
## genotypemut              0.058180   0.033831 78.000000   1.720 0.089450
## timepointW6             -0.210156   0.028852 78.000000  -7.284 2.26e-10
## timepointW9             -0.260729   0.032257 78.000000  -8.083 6.50e-12
## genotypehet:timepointW6  0.002595   0.035911 78.000000   0.072 0.942580
## genotypemut:timepointW6  0.015447   0.042058 78.000000   0.367 0.714413
## genotypehet:timepointW9  0.014363   0.039553 78.000000   0.363 0.717494
## genotypemut:timepointW9  0.161213   0.045332 78.000000   3.556 0.000643
##                            
## (Intercept)             ***
## genotypehet                
## genotypemut             .  
## timepointW6             ***
## timepointW9             ***
## genotypehet:timepointW6    
## genotypemut:timepointW6    
## genotypehet:timepointW9    
## genotypemut:timepointW9 ***
## ---
## Signif. codes:  0 '***' 0.001 '**' 0.01 '*' 0.05 '.' 0.1 ' ' 1
## 
## Correlation of Fixed Effects:
##             (Intr) gntyph gntypm tmpnW6 tmpnW9 gntyph:W6 gntypm:W6
## genotypehet -0.804                                                
## genotypemut -0.674  0.542                                         
## timepointW6 -0.791  0.636  0.533                                  
## timepointW9 -0.707  0.569  0.477  0.559                           
## gntypht:tW6  0.635 -0.790 -0.428 -0.803 -0.449                    
## gntypmt:tW6  0.542 -0.436 -0.804 -0.686 -0.383  0.551             
## gntypht:tW9  0.577 -0.717 -0.389 -0.456 -0.816  0.566     0.313   
## gntypmt:tW9  0.503 -0.405 -0.746 -0.398 -0.712  0.320     0.600   
##             gntyph:W9
## genotypehet          
## genotypemut          
## timepointW6          
## timepointW9          
## gntypht:tW6          
## gntypmt:tW6          
## gntypht:tW9          
## gntypmt:tW9  0.580   
## convergence code: 0
## boundary (singular) fit: see ?isSingular
```

```
CB.Hemispherelm.Signal <- cbdf %>% lmer(CB.Hemisphere.Mean/WholeBrain.Mean ~ 
    genotype * timepoint + (1 | mouse), data = .)
```

```
## boundary (singular) fit: see ?isSingular
```

```
CB.Hemisphere.SignalPairwise <- emmeans(CB.Hemispherelm.Signal, ~genotype * 
    timepoint)
pairs(CB.Hemisphere.SignalPairwise)
```

```
##  contrast         estimate     SE   df t.ratio p.value
##  wt,W3 - het,W3   0.008009 0.0219 78.0  0.366  1.0000 
##  wt,W3 - mut,W3  -0.040504 0.0261 78.0 -1.554  0.8262 
##  wt,W3 - wt,W6    0.007839 0.0222 76.5  0.354  1.0000 
##  wt,W3 - het,W6   0.047311 0.0202 78.0  2.337  0.3337 
##  wt,W3 - mut,W6   0.043459 0.0222 78.0  1.962  0.5741 
##  wt,W3 - wt,W9    0.021914 0.0249 75.5  0.881  0.9934 
##  wt,W3 - het,W9   0.048808 0.0213 78.0  2.296  0.3573 
##  wt,W3 - mut,W9  -0.081079 0.0232 78.0 -3.490  0.0215 
##  het,W3 - mut,W3 -0.048514 0.0233 78.0 -2.086  0.4904 
##  het,W3 - wt,W6  -0.000171 0.0188 78.0 -0.009  1.0000 
##  het,W3 - het,W6  0.039301 0.0165 74.1  2.386  0.3071 
##  het,W3 - mut,W6  0.035449 0.0188 78.0  1.889  0.6233 
##  het,W3 - wt,W9   0.013905 0.0220 78.0  0.633  0.9994 
##  het,W3 - het,W9  0.040798 0.0177 72.5  2.310  0.3501 
##  het,W3 - mut,W9 -0.089089 0.0200 78.0 -4.449  0.0009 
##  mut,W3 - wt,W6   0.048343 0.0235 78.0  2.054  0.5117 
##  mut,W3 - het,W6  0.087815 0.0218 78.0  4.037  0.0038 
##  mut,W3 - mut,W6  0.083963 0.0235 76.3  3.567  0.0172 
##  mut,W3 - wt,W9   0.062418 0.0262 78.0  2.386  0.3063 
##  mut,W3 - het,W9  0.089312 0.0227 78.0  3.935  0.0053 
##  mut,W3 - mut,W9 -0.040575 0.0245 75.8 -1.654  0.7716 
##  wt,W6 - het,W6   0.039472 0.0169 78.0  2.341  0.3313 
##  wt,W6 - mut,W6   0.035620 0.0191 78.0  1.864  0.6400 
##  wt,W6 - wt,W9    0.014075 0.0222 46.3  0.634  0.9993 
##  wt,W6 - het,W9   0.040969 0.0181 78.0  2.268  0.3739 
##  wt,W6 - mut,W9  -0.088918 0.0203 78.0 -4.370  0.0012 
##  het,W6 - mut,W6 -0.003852 0.0169 78.0 -0.228  1.0000 
##  het,W6 - wt,W9  -0.025397 0.0204 78.0 -1.247  0.9430 
##  het,W6 - het,W9  0.001497 0.0156 42.0  0.096  1.0000 
##  het,W6 - mut,W9 -0.128390 0.0182 78.0 -7.035  <.0001 
##  mut,W6 - wt,W9  -0.021545 0.0223 78.0 -0.968  0.9877 
##  mut,W6 - het,W9  0.005349 0.0181 78.0  0.296  1.0000 
##  mut,W6 - mut,W9 -0.124538 0.0203 39.8 -6.138  <.0001 
##  wt,W9 - het,W9   0.026894 0.0214 78.0  1.259  0.9401 
##  wt,W9 - mut,W9  -0.102993 0.0233 78.0 -4.415  0.0010 
##  het,W9 - mut,W9 -0.129887 0.0194 78.0 -6.708  <.0001 
## 
## Degrees-of-freedom method: kenward-roger 
## P value adjustment: tukey method for comparing a family of 9 estimates
```

```
summary(CB.Hemispherelm.Signal)
```

```
## Linear mixed model fit by REML. t-tests use Satterthwaite's method [
## lmerModLmerTest]
## Formula: CB.Hemisphere.Mean/WholeBrain.Mean ~ genotype * timepoint + (1 |  
##     mouse)
##    Data: .
## 
## REML criterion at convergence: -251.2
## 
## Scaled residuals: 
##     Min      1Q  Median      3Q     Max 
## -2.9063 -0.6584  0.1218  0.4488  2.9475 
## 
## Random effects:
##  Groups   Name        Variance  Std.Dev. 
##  mouse    (Intercept) 1.963e-18 1.401e-09
##  Residual             1.817e-03 4.262e-02
## Number of obs: 87, groups:  mouse, 55
## 
## Fixed effects:
##                          Estimate Std. Error        df t value Pr(>|t|)
## (Intercept)              0.981223   0.017401 78.000000  56.390   <2e-16
## genotypehet             -0.008010   0.021632 78.000000  -0.370   0.7122
## genotypemut              0.040504   0.025809 78.000000   1.569   0.1206
## timepointW6             -0.007839   0.022010 78.000000  -0.356   0.7227
## timepointW9             -0.021914   0.024608 78.000000  -0.891   0.3759
## genotypehet:timepointW6 -0.031463   0.027396 78.000000  -1.148   0.2543
## genotypemut:timepointW6 -0.076124   0.032085 78.000000  -2.373   0.0201
## genotypehet:timepointW9 -0.018884   0.030174 78.000000  -0.626   0.5332
## genotypemut:timepointW9  0.062489   0.034583 78.000000   1.807   0.0746
##                            
## (Intercept)             ***
## genotypehet                
## genotypemut                
## timepointW6                
## timepointW9                
## genotypehet:timepointW6    
## genotypemut:timepointW6 *  
## genotypehet:timepointW9    
## genotypemut:timepointW9 .  
## ---
## Signif. codes:  0 '***' 0.001 '**' 0.01 '*' 0.05 '.' 0.1 ' ' 1
## 
## Correlation of Fixed Effects:
##             (Intr) gntyph gntypm tmpnW6 tmpnW9 gntyph:W6 gntypm:W6
## genotypehet -0.804                                                
## genotypemut -0.674  0.542                                         
## timepointW6 -0.791  0.636  0.533                                  
## timepointW9 -0.707  0.569  0.477  0.559                           
## gntypht:tW6  0.635 -0.790 -0.428 -0.803 -0.449                    
## gntypmt:tW6  0.542 -0.436 -0.804 -0.686 -0.383  0.551             
## gntypht:tW9  0.577 -0.717 -0.389 -0.456 -0.816  0.566     0.313   
## gntypmt:tW9  0.503 -0.405 -0.746 -0.398 -0.712  0.320     0.600   
##             gntyph:W9
## genotypehet          
## genotypemut          
## timepointW6          
## timepointW9          
## gntypht:tW6          
## gntypmt:tW6          
## gntypht:tW9          
## gntypmt:tW9  0.580   
## convergence code: 0
## boundary (singular) fit: see ?isSingular
```

```
CB.Vermislm.Signal <- cbdf %>% lmer(CB.Vermis.Mean/WholeBrain.Mean ~ genotype * 
    timepoint + (1 | mouse), data = .)

CB.Vermis.SignalPairwise <- emmeans(CB.Vermislm.Signal, ~genotype * timepoint)
pairs(CB.Vermis.SignalPairwise)
```

```
##  contrast         estimate     SE   df t.ratio p.value
##  wt,W3 - het,W3   0.030588 0.0262 78.0  1.170  0.9605 
##  wt,W3 - mut,W3  -0.051845 0.0312 77.9 -1.662  0.7671 
##  wt,W3 - wt,W6    0.014471 0.0264 77.3  0.548  0.9998 
##  wt,W3 - het,W6   0.046766 0.0243 77.8  1.928  0.5967 
##  wt,W3 - mut,W6   0.040292 0.0265 77.8  1.518  0.8439 
##  wt,W3 - wt,W9   -0.000635 0.0296 75.5 -0.021  1.0000 
##  wt,W3 - het,W9   0.041753 0.0254 78.0  1.641  0.7793 
##  wt,W3 - mut,W9  -0.129863 0.0278 77.9 -4.668  0.0004 
##  het,W3 - mut,W3 -0.082433 0.0278 78.0 -2.964  0.0896 
##  het,W3 - wt,W6  -0.016117 0.0225 77.8 -0.717  0.9984 
##  het,W3 - het,W6  0.016178 0.0195 74.3  0.829  0.9956 
##  het,W3 - mut,W6  0.009703 0.0225 77.8  0.432  1.0000 
##  het,W3 - wt,W9  -0.031223 0.0263 78.0 -1.188  0.9567 
##  het,W3 - het,W9  0.011165 0.0209 72.0  0.535  0.9998 
##  het,W3 - mut,W9 -0.160451 0.0240 78.0 -6.693  <.0001 
##  mut,W3 - wt,W6   0.066316 0.0282 77.9  2.353  0.3247 
##  mut,W3 - het,W6  0.098611 0.0260 77.9  3.788  0.0086 
##  mut,W3 - mut,W6  0.092136 0.0280 76.9  3.288  0.0384 
##  mut,W3 - wt,W9   0.051210 0.0313 78.0  1.636  0.7818 
##  mut,W3 - het,W9  0.093598 0.0272 78.0  3.447  0.0244 
##  mut,W3 - mut,W9 -0.078018 0.0292 76.2 -2.675  0.1743 
##  wt,W6 - het,W6   0.032295 0.0202 77.5  1.596  0.8043 
##  wt,W6 - mut,W6   0.025820 0.0229 77.5  1.126  0.9685 
##  wt,W6 - wt,W9   -0.015106 0.0254 43.3 -0.594  0.9996 
##  wt,W6 - het,W9   0.027282 0.0217 77.9  1.260  0.9398 
##  wt,W6 - mut,W9  -0.144334 0.0244 77.8 -5.914  <.0001 
##  het,W6 - mut,W6 -0.006475 0.0202 77.5 -0.320  1.0000 
##  het,W6 - wt,W9  -0.047402 0.0244 78.0 -1.944  0.5860 
##  het,W6 - het,W9 -0.005013 0.0178 39.4 -0.282  1.0000 
##  het,W6 - mut,W9 -0.176629 0.0219 77.9 -8.071  <.0001 
##  mut,W6 - wt,W9  -0.040927 0.0267 78.0 -1.535  0.8356 
##  mut,W6 - het,W9  0.001461 0.0217 77.9  0.067  1.0000 
##  mut,W6 - mut,W9 -0.170154 0.0231 37.3 -7.373  <.0001 
##  wt,W9 - het,W9   0.042388 0.0256 78.0  1.658  0.7697 
##  wt,W9 - mut,W9  -0.129228 0.0279 78.0 -4.626  0.0005 
##  het,W9 - mut,W9 -0.171616 0.0232 78.0 -7.397  <.0001 
## 
## Degrees-of-freedom method: kenward-roger 
## P value adjustment: tukey method for comparing a family of 9 estimates
```

```
summary(CB.Vermislm.Signal)
```

```
## Linear mixed model fit by REML. t-tests use Satterthwaite's method [
## lmerModLmerTest]
## Formula: CB.Vermis.Mean/WholeBrain.Mean ~ genotype * timepoint + (1 |  
##     mouse)
##    Data: .
## 
## REML criterion at convergence: -223
## 
## Scaled residuals: 
##     Min      1Q  Median      3Q     Max 
## -2.3205 -0.5722  0.0282  0.4419  2.3764 
## 
## Random effects:
##  Groups   Name        Variance  Std.Dev.
##  mouse    (Intercept) 0.0003005 0.01733 
##  Residual             0.0023186 0.04815 
## Number of obs: 87, groups:  mouse, 55
## 
## Fixed effects:
##                           Estimate Std. Error         df t value Pr(>|t|)
## (Intercept)              1.0383770  0.0208564 77.9013371  49.787   <2e-16
## genotypehet             -0.0305883  0.0259197 77.9540027  -1.180   0.2415
## genotypemut              0.0518447  0.0309284 77.9395079   1.676   0.0977
## timepointW6             -0.0144713  0.0261995 77.3327396  -0.552   0.5823
## timepointW9              0.0006351  0.0291934 75.6840758   0.022   0.9827
## genotypehet:timepointW6 -0.0017069  0.0325468 76.6104693  -0.052   0.9583
## genotypemut:timepointW6 -0.0776650  0.0381758 77.1672680  -2.034   0.0453
## genotypehet:timepointW9 -0.0117999  0.0357356 74.7486432  -0.330   0.7422
## genotypemut:timepointW9  0.0773831  0.0410488 76.0184678   1.885   0.0632
##                            
## (Intercept)             ***
## genotypehet                
## genotypemut             .  
## timepointW6                
## timepointW9                
## genotypehet:timepointW6    
## genotypemut:timepointW6 *  
## genotypehet:timepointW9    
## genotypemut:timepointW9 .  
## ---
## Signif. codes:  0 '***' 0.001 '**' 0.01 '*' 0.05 '.' 0.1 ' ' 1
## 
## Correlation of Fixed Effects:
##             (Intr) gntyph gntypm tmpnW6 tmpnW9 gntyph:W6 gntypm:W6
## genotypehet -0.805                                                
## genotypemut -0.674  0.543                                         
## timepointW6 -0.787  0.633  0.531                                  
## timepointW9 -0.701  0.564  0.473  0.590                           
## gntypht:tW6  0.633 -0.785 -0.427 -0.805 -0.475                    
## gntypmt:tW6  0.540 -0.434 -0.801 -0.686 -0.405  0.552             
## gntypht:tW9  0.573 -0.710 -0.386 -0.482 -0.817  0.597     0.331   
## gntypmt:tW9  0.499 -0.401 -0.741 -0.420 -0.711  0.338     0.631   
##             gntyph:W9
## genotypehet          
## genotypemut          
## timepointW6          
## timepointW9          
## gntypht:tW6          
## gntypmt:tW6          
## gntypht:tW9          
## gntypmt:tW9  0.581
```

# Figure 6 - Genotype-wise cerebellar layer volume trends

## Main 3 layers

```
ggsave("wholebrainsignaltrics.png", wholebrainsignaltrics, scale = 3, dpi = "retina", 
    width = 6, height = 3)
```

```
## Warning: Removed 2 rows containing non-finite values (stat_summary).

## Warning: Removed 2 rows containing non-finite values (stat_summary).
```

```
CBsubstructureplot <- gf %>% mutate(`Cb Cortex` = CB.Cortex.Volume, `Cb White Matter` = CB.WhiteMatter.Volume, 
    `Cb Nuclei` = CB.Nuclei.Volume) %>% gather(roi, volume, `Cb Cortex`, `Cb White Matter`, 
    `Cb Nuclei`) %>% mutate(roi = fct_relevel(roi, "Cb Cortex", "Cb White Matter")) %>% 
    ggplot() + aes(x = time, y = volume, color = genotype) + stat_summary(fun.data = mean_cl_boot, 
    geom = "line") + stat_summary(fun.data = mean_cl_boot) + facet_wrap(~roi, 
    nrow = 1, scales = "free", shrink = TRUE) + ylab(bquote(bold("Volume" ~ 
    (mm^3)))) + xlab("Age (Weeks)") + scale_color_discrete("Genotype", labels = labeller) + 
    scale_x_continuous(breaks = c(3, 6, 9)) + scale_y_continuous(breaks = pretty_breaks(n = 8)) + 
    plottheme

CBsubstructureplot
```

```
ggsave("CBsubstructureplot.png", CBsubstructureplot, scale = 3, dpi = "retina", 
    width = 4, height = 2)
```

## Hemisphere vs Vermis

```
CBsubregionvolumeplot <- gf %>% mutate(`Cb Hemisphere` = CB.Hemisphere.Volume, 
    `Cb Vermis` = CB.Vermis.Volume) %>% gather(roi, volume, `Cb Hemisphere`, 
    `Cb Vermis`) %>% ggplot() + aes(x = time, y = volume, color = genotype) + 
    stat_summary(fun.data = mean_cl_boot, geom = "line") + stat_summary(fun.data = mean_cl_boot) + 
    facet_wrap(~roi, scales = "free") + xlab("Age (Weeks)") + ylab(bquote(bold("Volume" ~ 
    (mm^3)))) + scale_color_discrete("Genotype", labels = labeller) + scale_x_continuous(breaks = c(3, 
    6, 9)) + scale_y_continuous(breaks = pretty_breaks(n = 7)) + plottheme + 
    theme(legend.position = "none")

CBsubregionvolumeplot
```

```
ggsave("CBsubregionvolumeplot.png", CBsubregionvolumeplot, scale = 3, dpi = "retina", 
    width = 4, height = 2)
```

# Figure 7 - Genotype-wise cerebellar layer signal trends

```
CBsignalplot <- gf %>% mutate(`Cb Cortex` = CB.Cortex.Mean, `Cb White Matter` = CB.WhiteMatter.Mean, 
    `Cb Nuclei` = CB.Nuclei.Mean) %>% gather(roi, signal, `Cb Cortex`, `Cb White Matter`, 
    `Cb Nuclei`) %>% mutate(roi = fct_relevel(roi, "Cb Cortex", "Cb White Matter")) %>% 
    ggplot() + aes(x = time, y = signal/WholeBrain.Mean, color = genotype) + 
    stat_summary(fun.data = mean_cl_boot, geom = "line") + stat_summary(fun.data = mean_cl_boot) + 
    facet_wrap(~roi, nrow = 1, scales = "free") + ylab(bquote(bold("Signal (% of Whole Brain)"))) + 
    xlab("Age (Weeks)") + scale_y_continuous(breaks = pretty_breaks(n = 4), 
    limits = c(0.8, 1.4)) + ylab(bquote(bold("Relative Signal Intensity"))) + 
    scale_x_continuous(breaks = c(3, 6, 9)) + scale_color_discrete("Genotype", 
    labels = labeller) + plottheme

CBsignalplot
```

```
ggsave("CBsignalplot.png", CBsignalplot, scale = 3, dpi = "retina", width = 4, 
    height = 2)
```

# Registration accuracy testing

```
plotf <- function(ssm = NULL, nrow = 9, ncol = 1, begin = 40, end = 75, anatName, 
    overName, name, labels) {
    # anatVol <- anatName %>% mincGetVolume() %>% mincArray()
    overVol <- overName %>% mincGetVolume() %>% mincArray() %>% round()
    overVol[overVol %in% labels] <- -1
    overVol[!(overVol %in% labels) & overVol != -1] <- 0
    
    ssm %>% sliceSeries(nrow = nrow, ncol = ncol, begin = begin, end = end, 
        dimension = 1) %>% anatomy(volume = anatVol, low = 1000, high = 1500) %>% 
        contours(overVol, levels = -0.5, col = "red") %>% addtitle(name)
}


labels <- list()
for (name in c("Cerebellar cortex", "arbor vitae", "Cerebellar nuclei")) {
    labels[[name]] <- FindNode(hdefs, name)$Get("label") %>% .[[1]]
    
    name %>% str_replace_all(" ", "-") %>% str_c("figures2/", ., ".pdf") %>% 
        pdf(width = 333, height = 10)
    
    reduce(seq(nrow(gf)), function(acc, i) {
        acc %>% plotf(anatName = anatVol, overName = gf[[i, "label_file_DSURQE"]], 
            labels = labels[[name]], begin = 40, end = 75, nrow = 9, ncol = 1, 
            name = gf[[i, "mouse"]])
    }, .init = NULL) %>% legend(name) %>% draw()
    
    dev.off()
}
```

# Cerebellar lobule-specific volume and signal trends

```
lobuleCalc = FALSE

if (lobuleCalc) {
    Lobule2x3.Signal <- FindNode(hsums, "Central lobule")$volumes
    Lobule4x5.Signal <- FindNode(hsums, "Culmen")$volumes
    Lobule6.Signal <- FindNode(hsums, "Declive (VI)")$volumes
    Lobule7.Signal <- FindNode(hsums, "Folium-tuber vermis (VII)")$volumes
    Lobule8.Signal <- FindNode(hsums, "Pyramus (VIII)")$volumes
    Lobule9.Signal <- FindNode(hsums, "Uvula (IX)")$volumes
    Lobule10.Signal <- FindNode(hsums, "Nodulus (X)")$volumes
    Lobule2x3.Volume <- FindNode(hanat, "Central lobule")$volumes
    Lobule4x5.Volume <- FindNode(hanat, "Culmen")$volumes
    Lobule6.Volume <- FindNode(hanat, "Declive (VI)")$volumes
    Lobule7.Volume <- FindNode(hanat, "Folium-tuber vermis (VII)")$volumes
    Lobule8.Volume <- FindNode(hanat, "Pyramus (VIII)")$volumes
    Lobule9.Volume <- FindNode(hanat, "Uvula (IX)")$volumes
    Lobule10.Volume <- FindNode(hanat, "Nodulus (X)")$volumes
}
```

```
if (lobuleCalc) {
    CBlobulevolumeplot <- gf %>% mutate(Lobule2x3.Volume, Lobule4x5.Volume, 
        Lobule6.Volume, Lobule7.Volume, Lobule8.Volume, Lobule9.Volume, Lobule10.Volume) %>% 
        gather(roi, volume, Lobule2x3.Volume, Lobule4x5.Volume, Lobule6.Volume, 
            Lobule7.Volume, Lobule8.Volume, Lobule9.Volume, Lobule10.Volume) %>% 
        ggplot() + aes(x = time, y = volume, color = genotype) + stat_summary(fun.data = mean_cl_boot, 
        geom = "line") + stat_summary(fun.data = mean_cl_boot) + facet_wrap(~roi, 
        scales = "free") + xlab("Age (Weeks)") + ylab(bquote(bold("Volume" ~ 
        (mm^3)))) + scale_color_discrete("Genotype", labels = labeller) + scale_x_continuous(breaks = c(3, 
        6, 9)) + scale_y_continuous(breaks = pretty_breaks(n = 7)) + plottheme + 
        theme(legend.position = "none")
    
    CBlobulevolumeplot
}
```
