# Supplementary figures and images for: Longitudinal MEMRI analysis of brain phenotypes in a mouse model of Niemann-Pick Type C disease
Source: Neuroimage. Author manuscript; Available in PMC 2020 Aug 24. (PMC7443857; doi:10.1016/j.neuroimage.2020.116894)

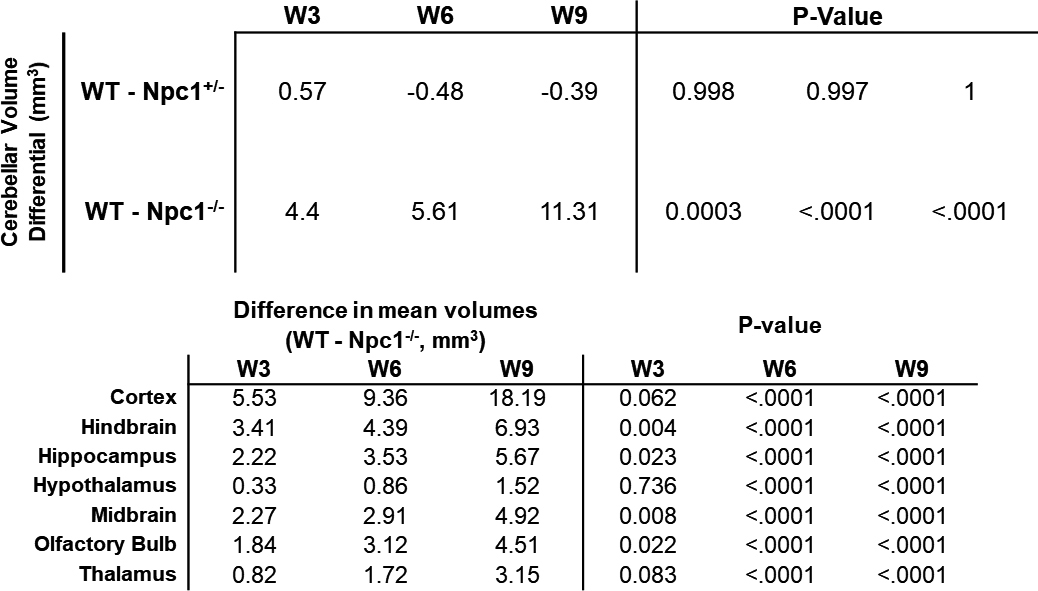

Supplement: Supplementary Table 1 [file NIHMS1609340-supplement-Supplementary_Table_1.jpg]

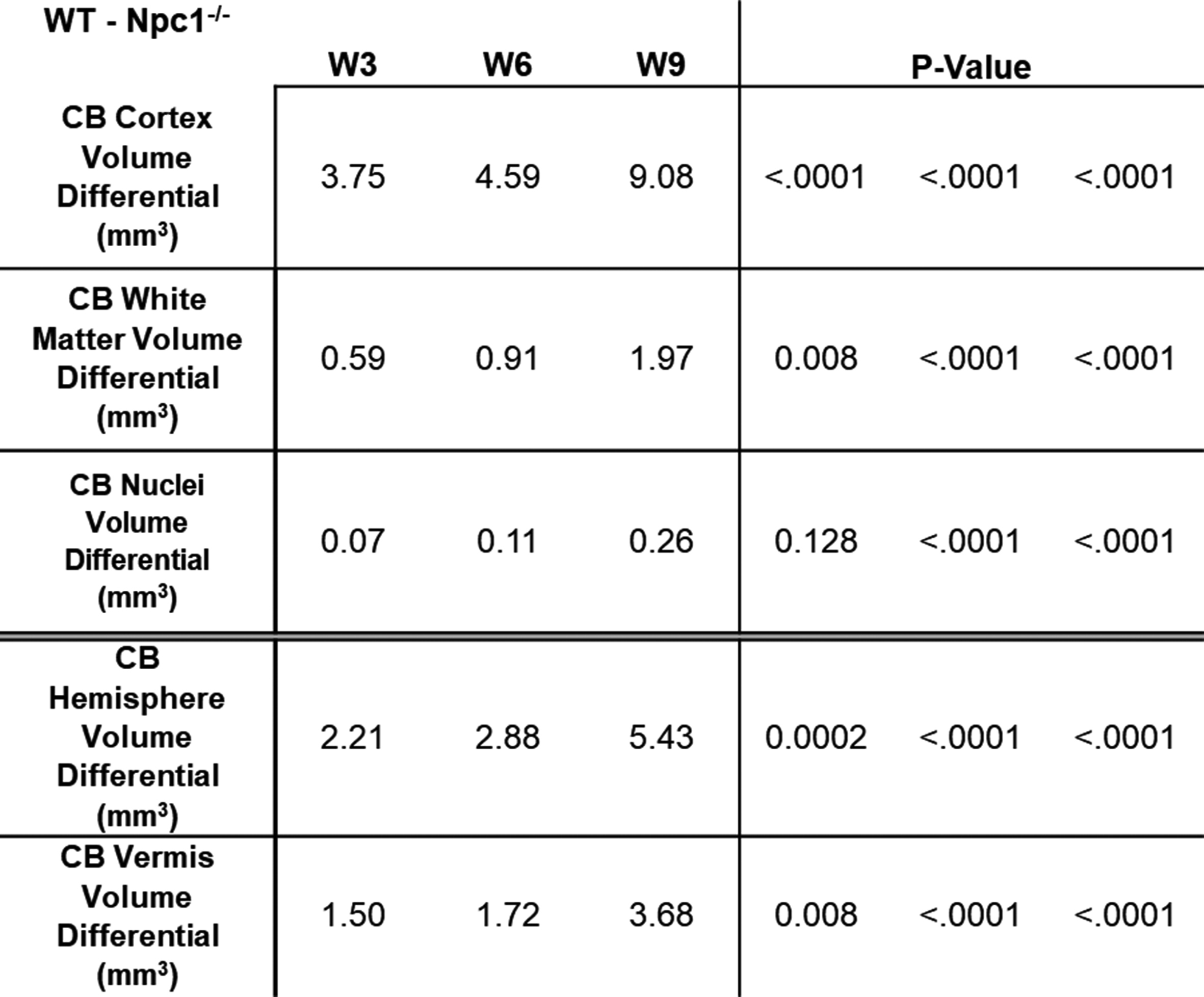

Supplement: Supplementary Table 3 [file NIHMS1609340-supplement-Supplementary_Table_3.jpg]

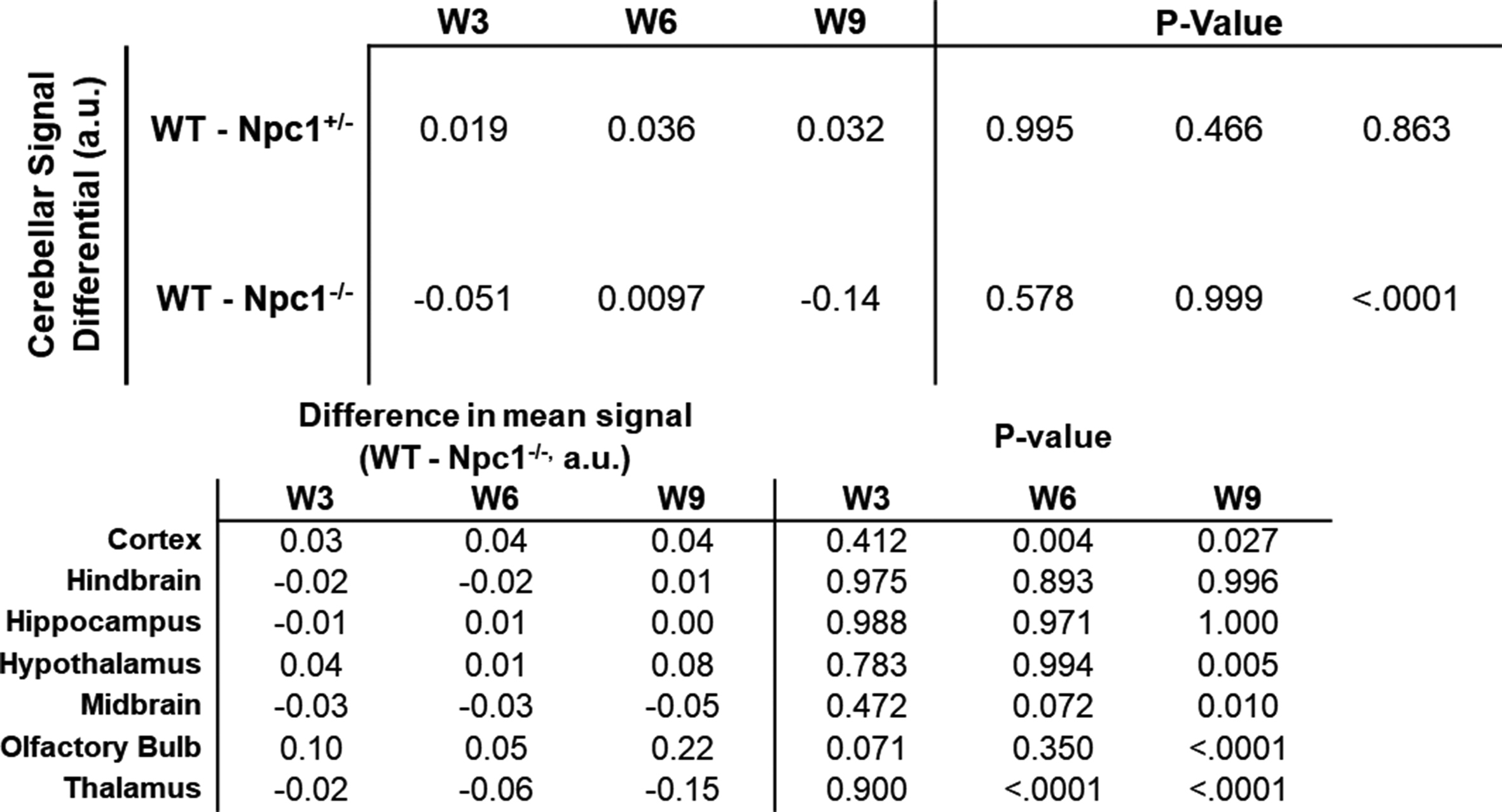

Supplement: Supplementary Table 2 [file NIHMS1609340-supplement-Supplementary_Table_2.jpg]

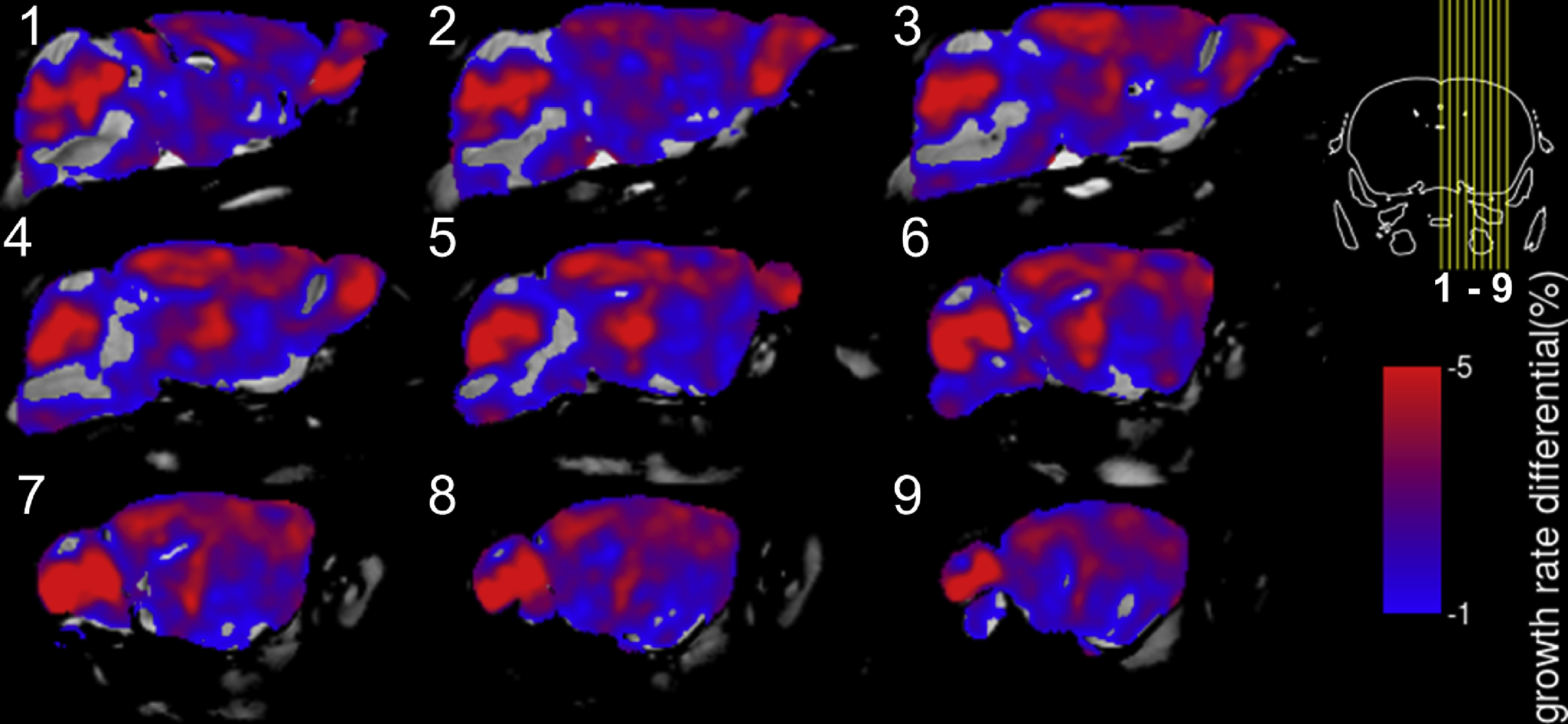

Supplement: Supplementary Figure 1 [file NIHMS1609340-supplement-Supplementary_Figure_1.jpg]

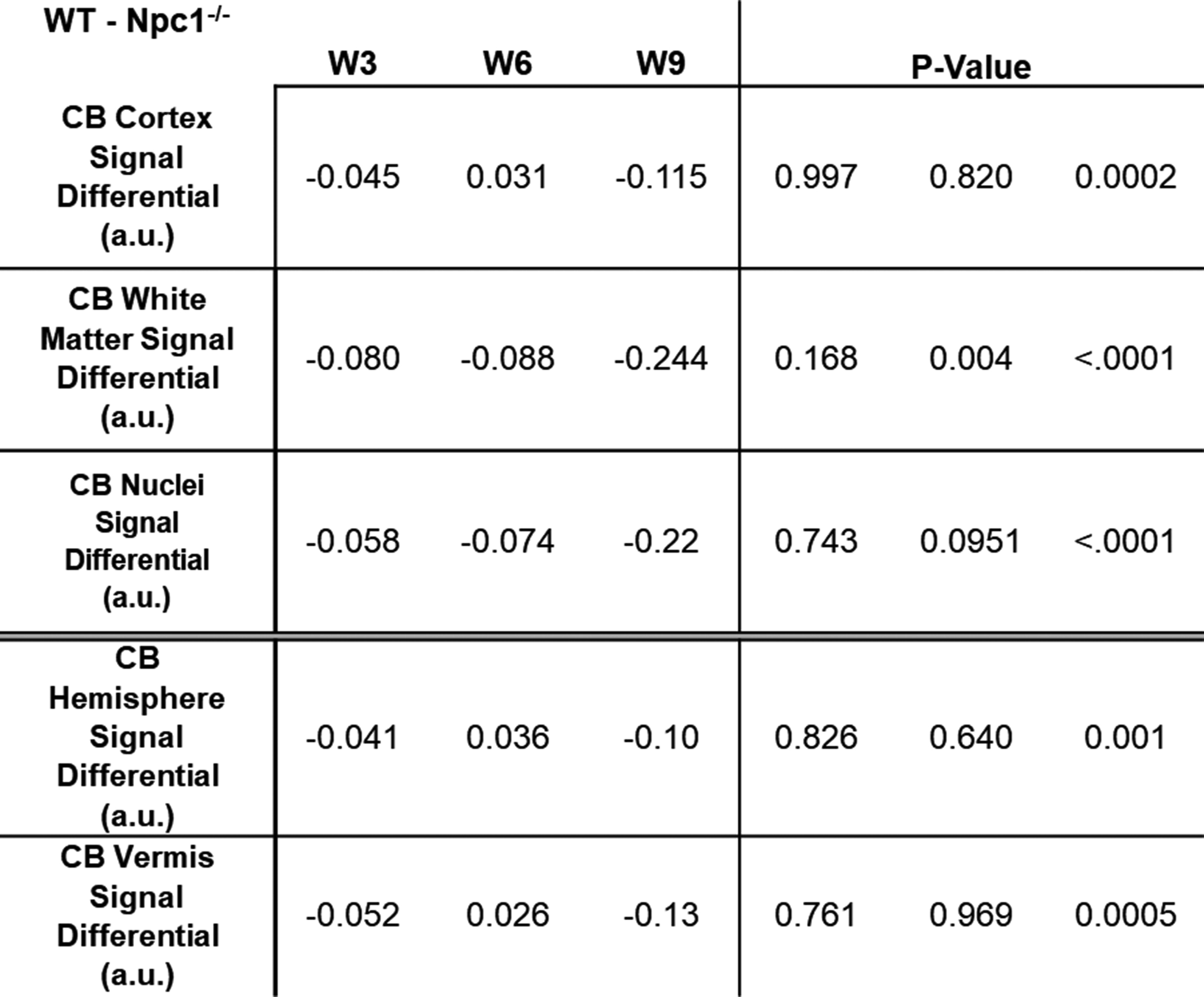

Supplement: Supplementary Table 4 [file NIHMS1609340-supplement-Supplementary_Table_4.jpg]
